# Alignment-free method for functional annotation of amino acid substitutions: Application on epigenetic factors involved in hematologic malignancies

**DOI:** 10.1371/journal.pone.0244948

**Published:** 2021-01-04

**Authors:** Branislava Gemović, Vladimir Perović, Radoslav Davidović, Tamara Drljača, Nevena Veljkovic

**Affiliations:** 1 Laboratory for Bioinformatics and Computational Chemistry, Vinča Institute of Nuclear Sciences, National Institute of the Republic of Serbia, University of Belgrade, Belgrade, Serbia; 2 Heliant d.o.o., Belgrade, Serbia; CNR, ITALY

## Abstract

For the last couple of decades, there has been a significant growth in sequencing data, leading to an extraordinary increase in the number of gene variants. This places a challenge on the bioinformatics research community to develop and improve computational tools for functional annotation of new variants. Genes coding for epigenetic regulators have important roles in cancer pathogenesis and mutations in these genes show great potential as clinical biomarkers, especially in hematologic malignancies. Therefore, we developed a model that specifically focuses on these genes, with an assumption that it would outperform general models in predicting the functional effects of amino acid substitutions. EpiMut is a standalone software that implements a sequence based alignment-free method. We applied a two-step approach for generating sequence based features, relying on the biophysical and biochemical indices of amino acids and the Fourier Transform as a sequence transformation method. For each gene in the dataset, the machine learning algorithm–Naïve Bayes was used for building a model for prediction of the neutral or disease-related status of variants. EpiMut outperformed state-of-the-art tools used for comparison, PolyPhen-2, SIFT and SNAP2. Additionally, EpiMut showed the highest performance on the subset of variants positioned outside conserved functional domains of analysed proteins, which represents an important group of cancer-related variants. These results imply that EpiMut can be applied as a first choice tool in research of the impact of gene variants in epigenetic regulators, especially in the light of the biomarker role in hematologic malignancies. EpiMut is freely available at https://www.vin.bg.ac.rs/180/tools/epimut.php.

## Introduction

Epigenetic modifiers are unique players in cancer pathogenesis. Mutations in these genes can alter the epigenetic landscape of many genes along with their expression, which affects key disease related pathways, including metabolic and apoptotic [1]. Cancers that are most connected with mutations in epigenetic factors are hematologic malignancies, which seem to represent “epigenetic diseases”–diseases driven by mutations in regulators of DNA modifications and post-translational modifications of histones [2]. Hematologic malignancies include lymphoid malignancies, such as plasma cell neoplasms, various lymphomas and lymphoid leukemias, and myeloid malignancies, such as acute myeloid leukemia (AML), myeloproliferative neoplasms and myelodysplastic syndrome. These diseases affect approximately 32 individuals per 100 thousand [3], with an average 5-year survival rate of 57% for lymphoid and 37% for myeloid neoplasms [4, 5]. Hematologic cancers are associated with age [3, 6, 7], which, considering the remarkable increase in global life expectancy in humans over the past decades [8], puts them in focus as an important and growing health issue.

Epigenetic factors include chromatin remodelling proteins, their cofactors, histones, histone chaperones and proteins that affect gene expression as a reaction to the DNA or RNA modifications. Epigenetic factors are comprehensively catalogued in the EpiFactors database [9]. Somatic mutations in these genes contribute to the onset and progression of hematologic malignancies and in many cases they represent markers associated with prognosis and response to therapies [10]. Mutations in DNMT3A, IDH1/2 and ASXL1 are promising candidates for the risk stratification parameters in AML patients [11], whereas mutations in four epigenetic factors, EZH2, ARID1A, EP300 and CREBBP, were annotated as risk stratification markers in follicular lymphoma [12]. Additionally, mutations in DNMT3A and TET2 can contribute to prediction of the response to therapy in myeloid malignancies [13, 14]. Mutations in DNMT3A, ASXL1, RUNX1, TP53, EZH2, CREBBP and EP300 are associated with the survival of patients with various hematologic malignancies [13, 15–17].

Cancer related somatic mutations are archived in the COSMIC (Catalogue of Somatic Mutations in Cancer) database [18]. Numerous epigenetic factors are catalogued in the COSMIC Gene Census, a list of genes with mutations that are causally implicated in cancer. However, there are several variations in these particular genes that do not represent somatic disease related mutations, yet they are neutral and frequently present in healthy individuals. Nevertheless, the human genome has on average approximately 10,000–11,000 non-synonymous variations in the coding regions [19]. Thus far, gene variations that are most frequently linked to human diseases are single nucleotide variations that lead to amino acid substitutions (AAS), and therefore the major focus in the field is placed on the computational tools that can automatically assess the potential impact of AAS on protein functions and their association with human diseases [20–22]. Most computational tools for functional annotation of AAS rely on the evolutionary concepts that deem amino acid positions conserved across multiple species as functionally important. Therefore, the majority of these tools use multiple sequence alignments (MSA) as a starting point for determining AAS at the conserved positions which can lead to annotations of these AAS as deleterious. SIFT [23] is a tool that bases its predictions solely on MSA, while many others, including PolyPhen-2 [24], PROVEAN [25], MutationTaster2 [26], PON-P2 [27], SNAP2 [28], etc., combine evolutionary information with sequence and structure data. PolyPhen-2, which is the most widely used tool, adopts the Naïve Bayes classifier with eight sequence-based and three structure-based features.

MSA-based methods do not scale well with the large amount of data gathered with the new sequencing methodologies [29, 30] and, additionally, there is increasing evidence that conservation-based inference does not correlate highly with protein sequence positions related to functional tuning [31], which puts a focus on alternative approaches, like alignment-free methods. These methods are primarily used for DNA and protein sequence comparison, consequently leading to development of many tools for genome-wide phylogeny, detection of regulatory elements in DNA, detection of horizontal gene transfer and protein sequence classification [32]. Computational efficacy of alignment-free methods can be illustrated with a Protein Map, a method for protein sequence comparison based on the vector representation of protein sequences using amino acid physicochemical characteristics, which is 13 times faster than comparable MSA-based methods [33]. Alignment-free methodology is not commonly used for this purpose and, according to the best of our knowledge, the only tool based on this approach is SNAP2_*noali*_ [28]. In our study, we developed an alignment-free method for estimating the effects of AAS–EpiMut.

## Methods

### Dataset

**Fig 1:**
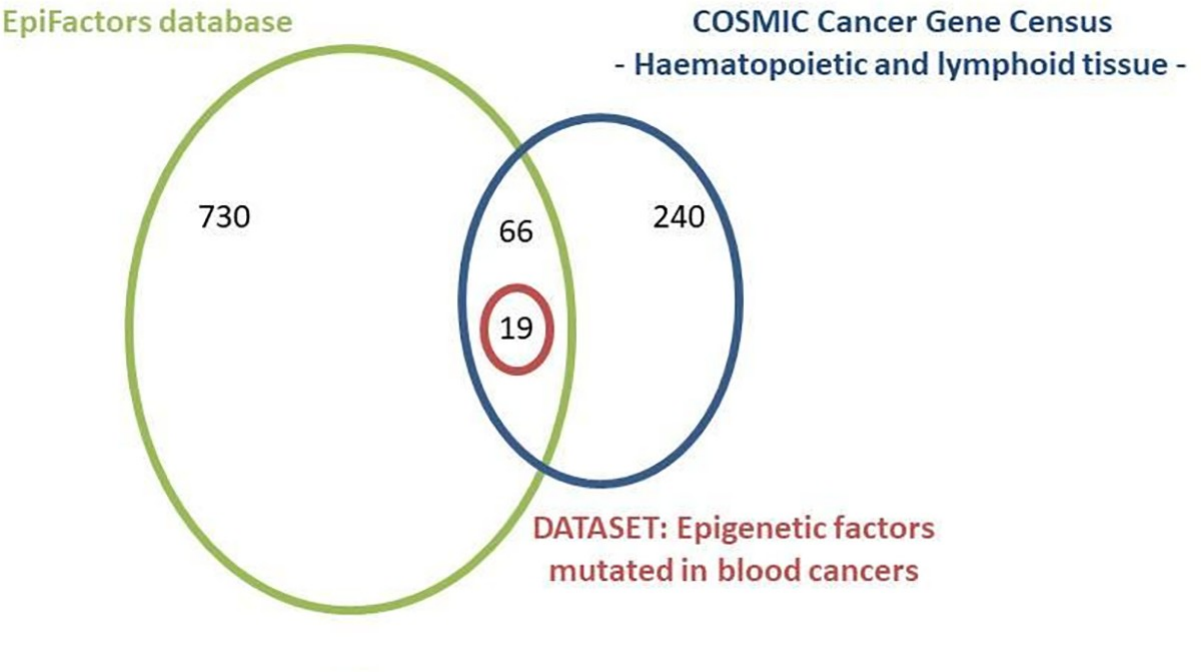
Selection of genes, using the COSMIC Cancer Gene Census—Haematopoietic and Lymphoid Tissue and EpiFactors database. There were at least 50 AAS in dbSNP and COSMIC for each of these 19 selected genes (in the red circle) that further constituted our dataset.

The dataset encompassed the epigenetic modifier genes that fulfil the following criteria: 1) are included in the COSMIC list of Cancer Gene Census for Haematopoietic and Lymphoid Tissue 2) are included in the EpiFactors database and 3) have more than 50 AAS in dbSNP [34] and COSMIC—Haematopoietic and Lymphoid Tissue, in total (Fig 1). Thus, sequences of 19 epigenetic regulators were obtained from the UniProt database [35], in the FASTA format. Conserved functional domains in these genes were retrieved from the Pfam database (version 31.0) [36].

Cancer associated mutation data were collected from the COSMIC database (v81). In the dataset, only SNPs from the reference transcripts of genes were included and, also, we included only SNPs satisfying criteria: “Chrom. Sample Cnt.” > = 100 AND “Variant allele frequency” > = 0.001 in the dbSNP (b151). We excluded ambiguous variants. The data collected from the databases didn’t contain any personal information.

### EpiMut features and scores

**Fig 2:**
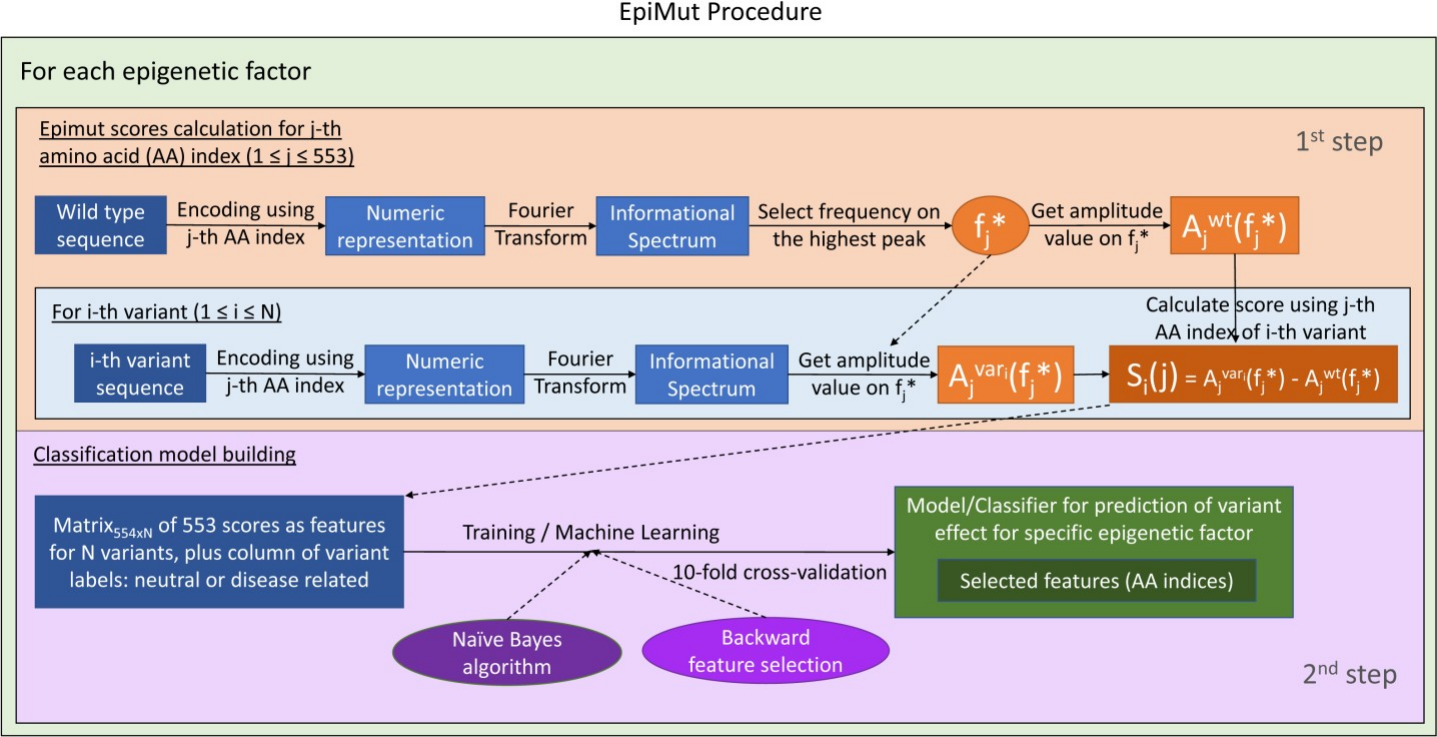
EpiMut procedure that was applied to each of the 19 proteins in the dataset.

We used a two-step approach for generating sequence based features (Fig 2). First, we conducted amino acid encoding of protein sequences. Encoding was done using indexes stored in the AAIndex, a comprehensive archive of various biochemical and biophysical amino acid indices [37]. We employed each of the 553 indices (out of 566) that had values for all amino acids. In the second step, we performed a Fourier Transform on each numerical representation of protein sequences. The Fourier Transform decomposes a numerical sequence into periodical functions, with series of frequencies and their amplitudes, represented by the informational spectrum [38]. Frequencies in the informational spectrum correspond to the distribution of structural motifs and we used this property to predict the effects of sequence variation on protein function.

Therefore, each wild type protein sequence was firstly transformed into 553 numerical sequences that were subsequently converted into 553 informational spectra by the Fourier Transform. An informational spectrum frequency with the highest amplitude value was selected for generation of EpiMut scores. The EpiMut score is defined as the difference between the amplitude value on the selected frequency in the sequence with the variant and the amplitude value on that particular frequency of the wild type. Therefore, each variant was represented with the vector encompassing 553 scores, as follows:

$$V_{i}=[S_{i}(1),\ldots ,S_{i}(M)]$$

$$S_{i}(j)=A_{j}^{{var}_{i}}(f_{j}^{*})-A_{j}^{wt}(f_{j}^{*}),i=1,2,\ldots ,N;j=1,2,\ldots ,M$$

where *f*_j_* is the frequency of the peak with the highest value of the amplitude in the informational spectrum of the wild type obtained using the *j*-th AA index as an encoder; *A*_*j*_^*vari*^*(f*_j_^*^) and *A*_*j*_^*wt*^*(f*_j_^*^) are the amplitudes on the frequency *f*_j_^*^ of the *i*-th variant and wild type informational spectra accordingly generated using the *j*-th AA index; *S*_*i*_*(j)* is the *j*-th EpiMut score (defined by the *j*-th AA index encoder) between the *i*-th variant and wild type; *V*_*i*_ is the 553 dimensional vector that represents the *i*-th variant; *N* is the number of variants in one protein from our dataset and it varies depending on the protein; and *M* is the number of used indices for amino acid encoding and it equals 553.

### EpiMut models and predictions

EpiMut relies on the Naïve Bayes classifier, built for each protein in the dataset, to generate predictions (Fig 2). We used the H2O platform for machine learning [39]. The Naïve Bayes algorithm implementation in H2O [40] was used for training and building the classification models. The dimensionality of training and test data was reduced through the use of the backward elimination process, the GreedyStepwise method, which performed the attribute selection, whereas for the evaluation of the attributes, the WrapperSubsetEval algorithm, from the Weka 3.8.1 environment [41], was applied. EpiMut was trained and validated using 10-fold cross-validation.

### Performance evaluation

We estimated the performance of the tested tools using various measures, which are based on: true positives (TP)–correctly predicted disease related variants, false positives (FP)–neutral substitutions incorrectly predicted to be disease-related, true negatives (TN)–correctly predicted neutrals and false negatives (FN)–disease-related variants incorrectly predicted to be neutral. We calculated the sensitivity, specificity and accuracy as follows:

Sensitivity=TP/(TP+FN)

Specificity=TN/(TN+FP)

Accuracy=(TP+TN)/(TP+TN+FP+FN)

Performance was additionally measured with the Matthews Correlation Coefficient (MCC):

$$MCC=\frac{\left(TP*TN-FP*FN\right)}{\sqrt{\left(TP+FP)*(TP+FN)*(TN+FP)*(TN+FN\right)}}$$

Finally, we generated the Receiver Operating Characteristic (ROC) curves and calculated areas under the ROC curves (AUC). A ROC curve shows the relative trade-off between the true positive rate and false positive rate when different thresholds are set to distinguish between the two classes and it is widely used as a measure of performance of binary classifiers.

For each statistic, the standard deviation (SD) was calculated as the difference between the value of the statistic for each gene (g_i_) and the overall performance (g_all_). The standard error (SE) was calculated by dividing SD by the number of analysed genes (n), which equals 19 in all cases.

$$SD=\sqrt{\frac{\sum {\left(g_{i}-g_{all}\right)}^{2}}{n}}$$

$$SE=\frac{SD}{\sqrt{n}}$$

### Comparison with other prediction tools

For the comparison of EpiMut with other tools for functional annotation of AAS, we used SIFT, PolyPhen-2 and SNAP2. SIFT uses sequence homology to predict the effect of an AAS on the protein function, considering the position at which the substitution occurred and the type of amino acid change. It calculates the probability score that indicates if the amino acid change is tolerated. In this study, we had to transform SIFT scores so they could be compared with other tools and we calculated the SIFTscore = 1 –SIFTscore*, where the SIFTscore* is the score originally retrieved from the SIFT tool. This transformation resulted in higher SIFT scores for disease-associated variants, and vice versa for neutral variants, which is in accordance with scores of the other three tools and could be applied to the calculation of comparable ROC curves. We used the single protein tool SIFT Sequence, with default values of median conservation of sequences (3.0). The PSI-BLAST search was applied to the UniRef90 database and sequences with a similarity level of 90% or more to the query sequence were removed from the alignment. Binary classification was done by annotating AAS with the SIFTscore > 0.95 as disease-related and AAS with a SIFTscore < 0.95 as neutral. Variants with a SIFTscore = 0.95 were classified as in output provided by the SIFT tool. The PolyPhen-2 bases its predictions of the damaging effects of missense mutations on eight sequence-based features (PSIC score of the wild-type amino acid, difference between the PSIC scores of the wild type and the mutant amino acids, the sequence identity to the closest homologue, congruency of the mutant allele to the multiple alignment, CpG context, alignment depth, change in the amino acid volume, whether the site of the mutation resides within an annotated Pfam domain) and three structure-based features (the accessible surface area, the change in the hydrophobic propensity, crystallographic B-factor. The functional effect of an AAS is predicted based on the calculated Naïve Bayes probabilistic score. A variant is automatically classified as “probably damaging”, “possibly damaging” or “benign”. For this study we adopted a binary classification, with a cut-off for a probabilistic score of 0.5, leading to annotating AAS with the higher scores as disease-related and those with lower scores as neutral. We used default values for query options and the HumVar-trained version of PolyPhen-2. SNAP2 is a neural networks based classifier. Its feature selection and training was done using various features, like biophysical amino acid properties, amino acid properties as provided by the AAindex database, explicit sequence, PSIC profiles, secondary structure and solvent accessibility, residue flexibility, SWISS-PROT annotations, residue annotations from Pfam and PROSITE, predicted binding residues, predicted disordered regions, proximity to the N- and C-terminus, statistical contact potentials, co-evolving positions and low-complexity regions. SNAP2 predicts the effect of a single AAS on protein function and it gives a binary prediction “effect”/“neutral” and a score ranging from -100 (strong neutral prediction) to +100 (strong effect prediction), which reflects the likelihood of this specific variation altering the native protein function.

## Results

### Gene specific models versus multiple genes model

We collected variants from COSMIC and dbSNP for 19 epigenetic factors mutated in hematologic malignancies. Our dataset contained 1303 disease-related and 1578 neutral variants (Table 1). The entire variants dataset is provided in the S1 Table in S1 File.

**Table 1:** The variants dataset consisted of 2881 variants in 19 epigenetic factors.

Gene	Disease-related variants	Neutral variants	Total number of variants
ARID1A	23	68	91
ASXL1	21	70	91
ATM	95	163	258
ATRX	48	56	104
BCOR	23	29	52
CREBBP	73	70	143
DNMT3A	111	14	125
EP300	68	88	156
EZH2	69	9	78
JAK2	40	41	81
KMT2A	34	90	124
KMT2C	96	251	347
KMT2D	84	208	292
NSD1	48	85	133
SETD2	29	104	133
SF3B1	49	5	54
SPEN	23	141	164
TET2	202	67	269
TP53	167	19	186
	1303	1578	2881

Two types of prediction models were built: (i) gene specific models (GSM) that comprise the feature selection and training process separately for each gene in the dataset, and (ii) the multiple genes model (MGM), one general model built for all variants in the dataset. In both approaches, the features were generated based on amino acid indices listed in the AAindex and using the Fourier Transform, as described in the Methods section, while Naive Bayes was used as the machine learning algorithm.

**Fig 3:**
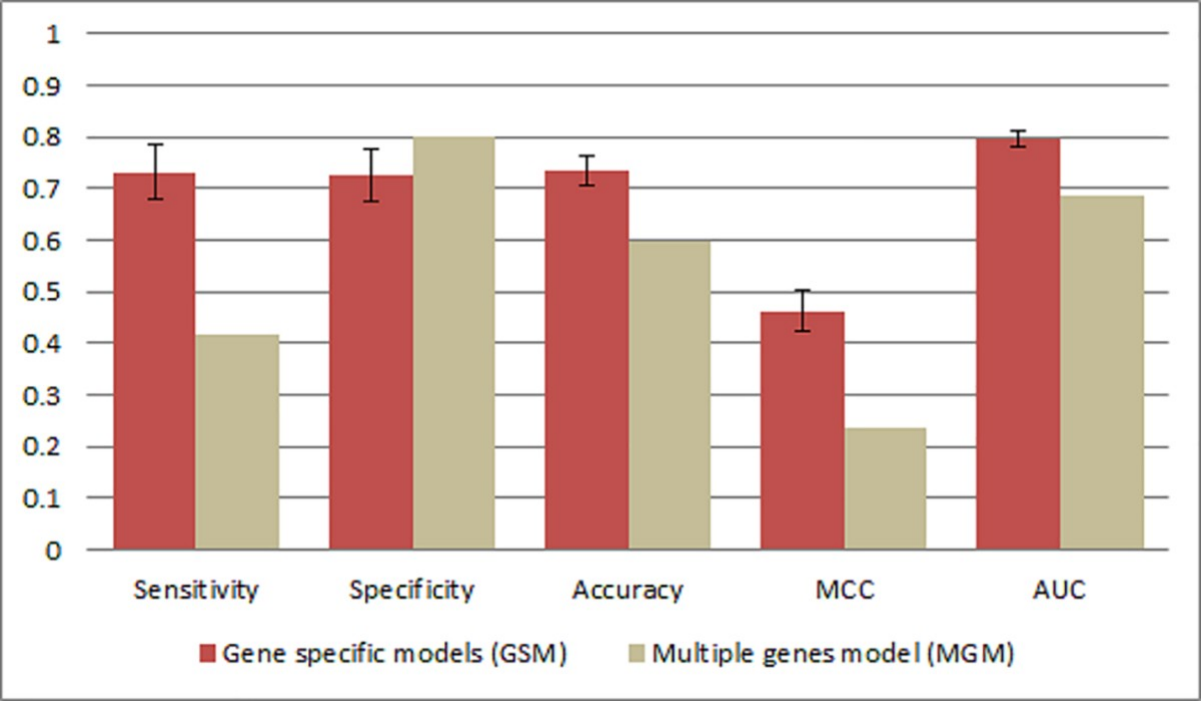
Comparison between the variant effect predictor based on Gene Specific Models (GSM) and the Multiple Genes Model (MGM).

The comparison of prediction capacities of these two approaches showed that the GSM method outperformed the MGM (Fig 3) and thus, the GSM was selected for further use in creating the EpiMut tool. Selected features in the GSM procedure for each of the 19 proteins are shown in the S2 Table in S1 File.

### Performance of EpiMut and comparison with state-of-the-art tools

**Fig 4:**
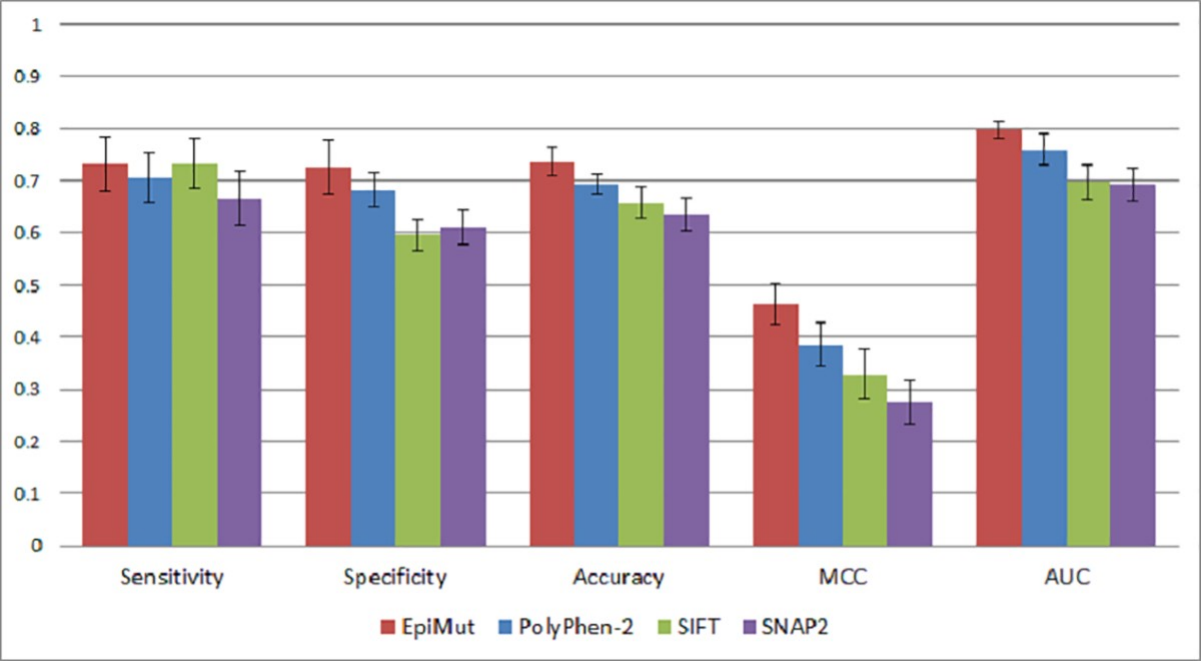
Performance of EpiMut, PolyPhen-2, SIFT and SNAP2 on a dataset consisting of variants in epigenetic factors mutated in hematologic malignancies.

Gene-specific and alignment-free methodology was used for the development of EpiMut, a tool for functional annotation of AAS in 19 analysed epigenetic factors. It provides probabilities for the predictions and the cut-off value of 0.5 was applied for binary classification, denoting AAS with the value ≥0.5 as “MUT” in the case of a disease-related prediction and AAS with the probability value <0.5 as “SNP” in the case of neutral predictions. We compared the performance of EpiMut with three state-of-the-art tools for functional annotation of AAS, PolyPhen-2, SIFT and SNAP2. Performance was measured using Sensitivity, Specificity, Accuracy and MCC. EpiMut showed a better performance compared to PolyPhen-2, SIFT and SNAP2 for each of these measures and, additionally, outperformed these tools in regards to AUC (Fig 4).

**Fig 5:**
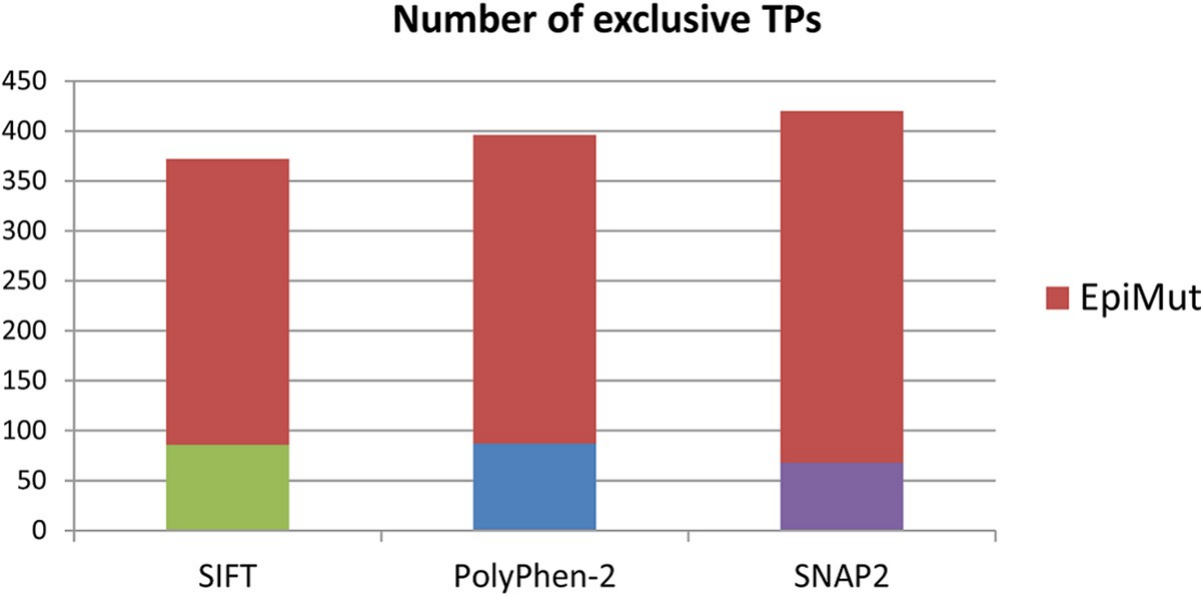
EpiMut is contrasted to SIFT, PolyPhen-2 and SNAP2 in the search for unshared correctly classified mutations. Stacked bars represent the numbers of exclusive TPs in each of the three comparisons.

Detailed comparison of methods performance was focused on the correctly classified mutations. In this step, we contrasted EpiMut to other methods and identified mutations that within each comparison were exclusively recognized by only one method and denoted those exclusive TPs. Noticeably, each of the methods reveals some of the mutations that were unobserved by the other one, but EpiMut is significantly dominant over its competitors (Fig 5). This analysis demonstrates that EpiMut improves our capacity to acquire new knowledge and accelerates experimental investigations in this complex field.

**Fig 6:**
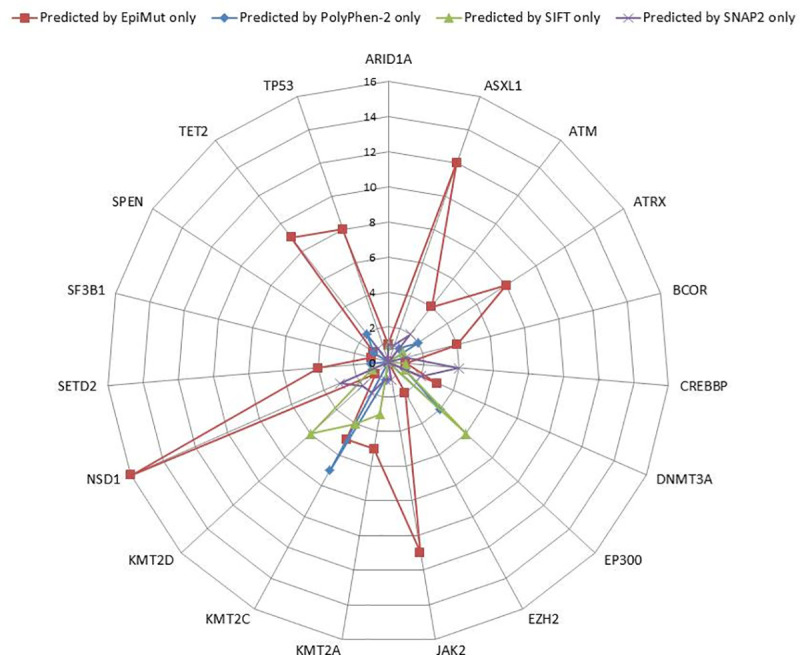
Distribution per gene of 162 “difficult to predict mutations” (DTP) predicted by EpiMut, PolyPhen-2, SIFT and SNAP2.

Further on, we inspected closely a set of variants miss-predicted by three out of four tools, which were labelled “difficult to predict mutations” (DTP). In the original dataset 12.4% (162 variants) were DTP disease-related variants. PolyPhen-2 correctly predicted 21, SIFT 24, SNAP2 21 and EpiMut 96 of these cases (Fig 6). To further investigate these 162 mutations, we searched available literature for additional information and the experimental evidence about their effects in obstructing the proteins’ normal functions and their involvement in human diseases. For the majority of these mutations, besides the information that they are associated with the disease, there was no detailed data about their effects on the protein function. This led us to focus further investigation on the three variations for which there was some detailed information regarding their effects in the available literature.

Further, we focused on two genes and the following variants: A1505T in TET2 and S46F and D48N in TP53 that were predicted as disease-related by EpiMut only. According to the literature, the TET2 mutation A1505T severely reduces the TET2 ability to bind protein WT1 [42]. WT1, a transcription factor involved in normal embryonic development of urogenital and hematopoietic systems, plays an important role in pathogenesis of hematologic malignancies, especially acute leukemias [43]. WT1 acts either as a tumour suppressor or oncogene depending on the cellular context and PPIs [44, 45]. Wang et al. showed that WT1 physically interacts with wild type TET2 and recruits it to its target genes, which affects expression of these genes and leads to inhibition of leukemia cell proliferation [42]. The A1505T mutation in TET2 disrupts this interaction, consequently leading to an effect on WT1 target genes expression and increased leukemia cell proliferation [42]. TET2 is frequently mutated in the majority of hematologic cancers, with a frequency of 17–37% in myeloid and 15–33% in T-cell lymphoid malignancies [46], and the disruption of its PPIs by mutations can be an important mechanism of its pathological role in these cases.

The second DTP we focused on is within TP53, a well-described tumour suppressor with roles in cell cycle regulation and apoptosis, which is mutated in many cancer types. In hematologic malignancies, the frequency of TP53 mutations ranges from 3–8% in AML to 10–20% in chronic lymphocytic leukemia [47]. More importantly, mutations in TP53 in hematologic cancers are associated with a more aggressive disease, worse overall survival and resistance to therapies [47]. Enari et al. showed that the S46F mutation in TP53 increases its binding to clathrin, a protein involved in vesicle transport [48]. This interaction is involved in apoptosis, although the mechanism remains elusive [48, 49]. Nevertheless, it was previously shown that impairment of clathrin’s normal functions, through gene fusions, leads toward various lymphoid malignancies [49].

Finally, aspartic acid at position 48 resides in the TAD2 domain of protein TP53 and is involved in the interaction with the Taz2 domain of histone acetyltransferase EP300 [50], another protein in our dataset. EP300 is an important player in pathogenesis of various lymphoid malignancies and its mutations are valuable biomedical markers in these diseases [12, 16]. TP53-EP300 interaction results in stabilization of TP53, its decreased degradation and increased gene transcription. Mutation D48N in TP53 reduces this interaction [50] and can consequently affect all of the mentioned functions and underlie pathogenic phenotypes [51]. According to the aforementioned findings, EpiMut efficiently identifies variants that disrupt protein interactions and support biological processes that underlie the disease mechanisms.

### Performance on the subset of variants positioned outside conserved functional domains

Although evolutionary based methodologies are almost ubiquitously used in tools for functional annotation of AAS, our previous research showed that SIFT and PolyPhen-2 have low sensitivity (51% and 39%, respectively) in predicting the functional effects of variants in protein regions of epigenetic modifiers that are outside of the conserved functional domains (CFD) [52]. It is important to address this issue since 50% of AAS associated with cancers were shown to be positioned in these non-CFDs (nCFD) [53]. Therefore, we tested the performance of EpiMut, PolyPhen-2, SIFT, and SNAP2 on the subset of 2108 nCFD variants (Table 2).

**Table 2:** nCFD dataset consisting of 2108 variants in non-conserved regions of epigenetic factors.

Gene	Disease-related variants	Neutral variants	Total number of variants
ARID1A	18	62	80
ASXL1	20	64	84
ATM	53	125	178
ATRX	39	52	91
BCOR	12	24	36
CREBBP	22	59	81
DNMT3A	51	10	61
EP300	36	77	113
EZH2	31	8	39
JAK2	14	22	36
KMT2A	29	83	112
KMT2C	86	232	318
KMT2D	64	199	263
NSD1	41	84	125
SETD2	22	104	126
SF3B1	48	5	53
SPEN	20	129	149
TET2	105	45	150
TP53	6	7	13
	717	1391	2108

**Fig 7:**
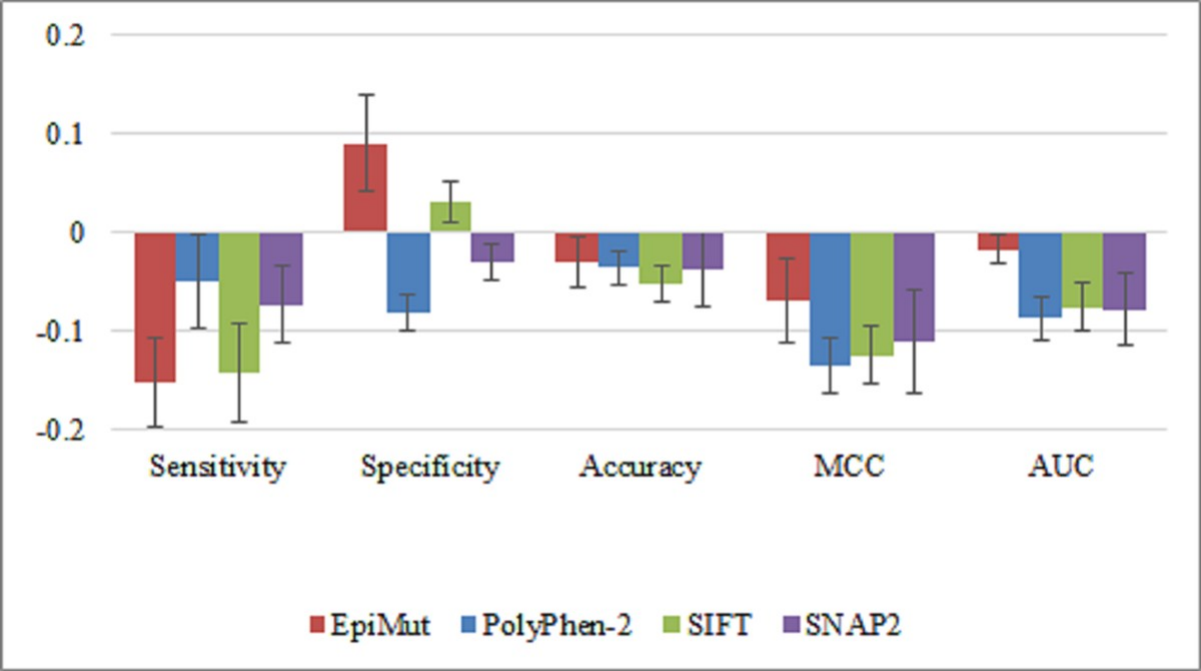
Differences in performance of EpiMut, PolyPhen-2, SIFT and SNAP2 between the entire variants dataset and nCFD data subset. Each column in this histogram represents the difference among the values of a particular performance measure obtained, for each tool, on the entire variants dataset and the values obtained for the nCFD data subset.

The decrease in performance on the nCFD dataset was observed for all prediction tools (Fig 7), whereas EpiMut shows the smallest decrease in accuracy, MCC and it retains a similar value of AUC, which is a consequence of the high increase in specificity (Fig 7). The performance of all tools on the nCFD dataset is shown in S1 Fig in S1 File, with EpiMut showing the best performance for all measures except sensitivity.

### EpiMut standalone tool

EpiMut software is implemented in the JAVA language, using the H2O library for generating machine learning classifiers, and is available as a standalone application, which can be executed on any operating system containing the Java Virtual Machine.

EpiMut supports a batch mode query for separate genes. The input file has to contain a list of AAS in the form of:

*original_amino_acid position_in_protein substitute_amino_acid* (example: G187V)

and the gene name of the selected gene for the query in the input file name. The generated output file contains the name of the query gene in the file name and the list of AAS with predicted class–SNP for neutral and MUT for disease-related variants, as well as probability associated with the prediction.

EpiMut is a free software released under the Apache License, Version 2.0. The EpiMut application with documentation is available at https://www.vin.bg.ac.rs/180/tools/epimut.php.

## Discussion

Machine learning (ML) methods are widely used for solving various biological classification problems, including inferring about the disease-related/neutral effects of AAS. The most commonly used approaches are support vector machines, neural networks, Bayesian classifiers, random forests and decision trees [54]. Two out of three tools that we used for performance comparison are ML-based–PolyPhen-2 employed Naïve Bayes and SNAP2 employed the neural networks approach. EpiMut encompasses a selection of different features and the Naive Bayes method as part of the GSM approach and thus it differs from the other tools. Comparison of the GSM strategy and commonly used approach of one ubiquitous model for all genes–the MGM model, showed that GSM-based EpiMut significantly outperforms MGM. This finding is in accordance with previous research showing that applying gene-specific thresholds to the results of standard tools, like PolyPhen-2, SIFT, Mutation Taster, Mutation Assessor [55], CADD [56], may improve their prediction performance [57, 58]. On the other hand, gene- or disease-specific models that are trained on relatively restricted but specific datasets satisfactorily associate gene variants with hyperthrophic cardiomyopathy [59], haemophilia [60] and various cardiac diseases [61]. The GSM approach that we developed employs variants collection of similar sizes as various previously developed methods for prediction of variant functional significance in: RET [62, 63], GLA [64] and DPYD [65]. It is important to notice that many previous studies also showed that the Bayesian approach is the method of choice for the prediction of effects of AAS when only small datasets are available [61, 62, 66]. Superiority of the GSM over the MGM points to a gene specific approach as the strategy for improving tools for functional annotation of gene variants and directions for the future development of this research field.

Performances of the PolyPhen-2 and SIFT on the variant dataset used in this study are similar to the results obtained in the study by Thusberg et al. [20], in which the authors evaluated various tools on the dataset containing approximately 40 thousand human AAS. The SNAP2 performance on our variant dataset was similar to the reported performance by the authors of the tool [28]. EpiMut showed a significant advantage in performance compared to these state-of-the-art tools for functional annotation of AAS. Accuracy of EpiMut was higher by 7.4% on average compared to other tools, while the AUC was higher by 8.0% on average. Nevertheless, one should bear in mind that different approaches underlying PolyPhen-2, SIFT, SNAP2 and EpiMut and especially various datasets that were used for the training of ML-based tools, make these comparisons difficult [54] given that the presence of a protein in a training dataset improves its performance for predicting the effects of different variants in the same protein [67]. In case of PolyPhen-2, 16 out of 19 proteins from our dataset were in its training set. Additionally, 36 variants in our dataset were already present in the PolyPhen-2 training set and it performed significantly better in predicting their effects compared to its average performance.

An important advantage of EpiMut which underlies its better performance on the nCFD dataset is that it doesn’t rely on the evolutionary information. Compared to other tools EpiMut has 8.5% higher accuracy and 14.4% higher AUC, on average. Lower accuracy of PolyPhen-2, SIFT and SNAP2 on the nCFD compared to the CFD set (14%, 13% and 20%, respectively) is in line with our previous results showing that MSA-based tools are not efficient for the prediction of functional effects of AAS outside CFDs [52]. The importance of this result is reflected in the fact that 55% of hematologic malignancy-related variants in analysed epigenetic factors, as well as 73% of all variants in the dataset are in nCFD and they are, therefore, predicted with lower efficacy by MSA-based tools.

Finally, approximately 12% of disease-related variants in our dataset were correctly predicted solely by one tool. Of these DTP variants, 60% were correctly predicted solely by EpiMut, which emphasizes the importance of the EpiMut workbench, combining the alignment-free and gene-specific approach. Almost none of the 162 DTP variants were previously functionally annotated in detail. Nevertheless, there was previous experimental verification of functional effects of three variants, in TET2 and TP53, showing their role in the PPI of these proteins [42, 48, 50]. Our recent research has shown that features generated on the basis of physicochemical characteristics of amino acids are important for understanding and predicting PPI [68]. This implies that characteristics of certain amino acids and their surrounding subsequences, captured through the use of the Fourier Transform for processing numerically encoded sequences, play crucial roles in protein interactions. EpiMut captures the effects of variants in these positions with high power. PPI interaction sites in the case of transient interactions are not under high evolutionary pressure and they vary to a great extent, which enables higher flexibility of these interactions [69]. This can have consequences in lower performance of MSA-based methods in predicting the functional effects of mutations. Other cases of mutations in epigenetic factors that alter PPI, playing an important role in pathogenesis of hematologic malignancies, are mutations in the SET domain of EZH2. This domain is crucial for EZH2 binding abilities [70] and mutations positioned in it contribute to the onset of lymphomas [71]. The result showing that EpiMut can correctly predict the majority of cases that were wrongly annotated by other tools, indicates the complementarity of EpiMut and the studied MSA-based tools, which can be used in the future for building new assembly methods.

It is worth noting that the methodology applied in this research, based on the gene-specific and alignment-free approach, can be used for the analysis of any gene mutated in human diseases. The only consideration that should be taken into account is the number of variants associated with a gene, which must satisfy requirements of the machine learning algorithms. Epigenetic factors in hematologic cancers provide a proof of concept and demonstrate the usefulness and effectiveness of the proposed approach. We provide EpiMut to serve the scientific community in predicting the functional effects of AAS and in future studies, we plan to further extend the scope of this methodology to additional genes involved in human cancers.

## Conclusions

Epigenetic factors are frequently mutated in hematologic malignancies and new variants are being discovered at an unprecedented pace. Numerous variants in genes coding for epigenetic regulators have already been identified as biomarkers for prognosis and therapy response, and computational models that effectively distinguish neutral from disease variants are in great demand. In this paper, we described a fast and computationally efficient EpiMut method that significantly improves variants effect predictions. EpiMut, especially, exceeds state-of-the-art tools in predicting the effects of difficult variants and functionally important variants positioned outside the conserved domains of proteins. The standalone EpiMut software that we contribute to the community has the potential to advance whole genome sequencing analysis pipelines for hematologic patients and to accelerate biomarker discovery.

## Supporting information

S1 File (PDF)
