# Supplementary material for: Alignment-free method for functional annotation of amino acid substitutions: Application on epigenetic factors involved in hematologic malignancies
Source: PLoS One. 2021 Jan 4;16(1):e0244948. doi: 10.1371/journal.pone.0244948 (PMC7781373; doi:10.1371/journal.pone.0244948)
Supplement: S1 File — (PDF) [file pone.0244948.s001.pdf]

**Alignment-free method for functional annotation of amino acid substitutions: application on epigenetic factors involved in hematologic malignancies**

*Gemović B, Perović V, Davidović R, Drljača T, Veljkovic N*

# SUPPLEMENTARY MATERIAL

S1 File. Performance of EpiMut, PolyPhen-2, SIFT and SNAP2 on the nCFD dataset.

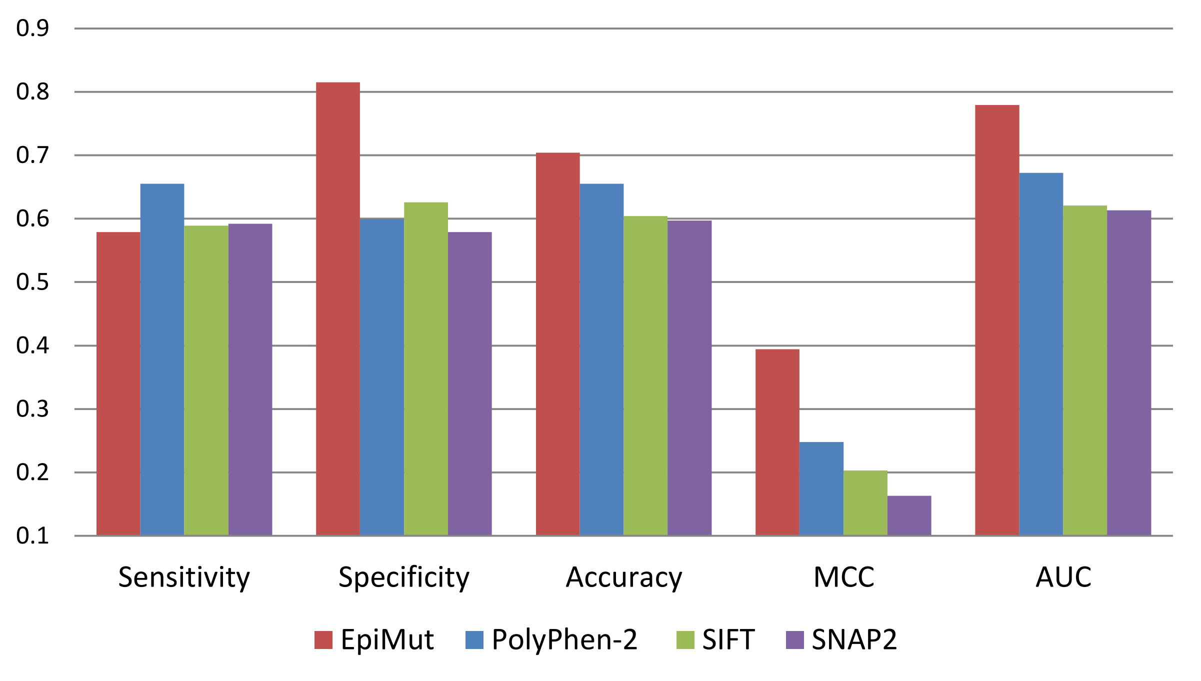


Supplementary Table 1. EpiMut dataset, containing 1303 somatic mutations and 1578 neutral SNPs in 19 epigenetic factors mutated in blood malignancies. Somatic mutations were obtained from COSMIC database, while neutral SNPs were obtained from dbSNP.

| Gene | AAS | Pathogenic status | CFD or nCFD | Gene | AAS | Pathogenic status | CFD or nCFD |
|---|---|---|---|---|---|---|---|
| ARID1A | A1077G | MUT | CFD | KMT2C | G2487D | SNP | nCFD |
| ARID1A | A873V | MUT | nCFD | KMT2C | G2888D | SNP | nCFD |
| ARID1A | C1981R | MUT | CFD | KMT2C | G315C | SNP | CFD |
| ARID1A | M618V | MUT | nCFD | KMT2C | G4135A | SNP | nCFD |
| ARID1A | N629K | MUT | nCFD | KMT2C | G838S | SNP | nCFD |
| ARID1A | P1147L | MUT | nCFD | KMT2C | G908C | SNP | nCFD |
| ARID1A | P1163L | MUT | nCFD | KMT2C | H1574R | SNP | nCFD |
| ARID1A | P1447S | MUT | nCFD | KMT2C | H2320R | SNP | nCFD |
| ARID1A | P1456L | MUT | nCFD | KMT2C | H365Q | SNP | nCFD |
| ARID1A | P1601S | MUT | nCFD | KMT2C | H3936L | SNP | nCFD |
| ARID1A | P1627A | MUT | nCFD | KMT2C | H4130D | SNP | nCFD |
| ARID1A | P1739R | MUT | nCFD | KMT2C | H4339P | SNP | nCFD |

| | | | | | | | |
|---|---|---|---|---|---|---|---|
| ARID1A | P2102H | MUT | CFD | KMT2C | H4466Q | SNP | CFD |
| ARID1A | P251S | MUT | nCFD | KMT2C | H4900Y | SNP | nCFD |
| ARID1A | P408L | MUT | nCFD | KMT2C | I1434N | SNP | nCFD |
| ARID1A | P870T | MUT | nCFD | KMT2C | I1862V | SNP | nCFD |
| ARID1A | R1463H | MUT | nCFD | KMT2C | I2781V | SNP | nCFD |
| ARID1A | R1879W | MUT | nCFD | KMT2C | I3087T | SNP | nCFD |
| ARID1A | S1570C | MUT | nCFD | KMT2C | I323V | SNP | CFD |
| ARID1A | S1707G | MUT | nCFD | KMT2C | I3493V | SNP | nCFD |
| ARID1A | S2002F | MUT | CFD | KMT2C | I3576V | SNP | nCFD |
| ARID1A | S506P | MUT | nCFD | KMT2C | I3596T | SNP | nCFD |
| ARID1A | V1982I | MUT | CFD | KMT2C | I4080T | SNP | nCFD |
| ARID1A | A1872V | SNP | nCFD | KMT2C | I4084L | SNP | nCFD |
| ARID1A | A1877P | SNP | nCFD | KMT2C | I4084V | SNP | nCFD |
| ARID1A | A1927P | SNP | nCFD | KMT2C | I4379V | SNP | nCFD |
| ARID1A | A221V | SNP | nCFD | KMT2C | I455M | SNP | nCFD |
| ARID1A | A226V | SNP | nCFD | KMT2C | I455V | SNP | nCFD |
| ARID1A | A900S | SNP | nCFD | KMT2C | I811V | SNP | nCFD |
| ARID1A | A900T | SNP | nCFD | KMT2C | I823N | SNP | nCFD |
| ARID1A | D1893E | SNP | nCFD | KMT2C | I823T | SNP | nCFD |
| ARID1A | E1291D | SNP | nCFD | KMT2C | K170E | SNP | nCFD |
| ARID1A | E1779G | SNP | nCFD | KMT2C | K1717R | SNP | nCFD |
| ARID1A | G1016S | SNP | nCFD | KMT2C | K2398Q | SNP | nCFD |
| ARID1A | G1254S | SNP | nCFD | KMT2C | K3221E | SNP | nCFD |
| ARID1A | G1255R | SNP | nCFD | KMT2C | K339N | SNP | nCFD |
| ARID1A | G125S | SNP | nCFD | KMT2C | K3940R | SNP | nCFD |
| ARID1A | G1293A | SNP | nCFD | KMT2C | L1452I | SNP | nCFD |
| ARID1A | G180A | SNP | nCFD | KMT2C | L2420R | SNP | nCFD |
| ARID1A | G275A | SNP | nCFD | KMT2C | L2653P | SNP | nCFD |
| ARID1A | G275V | SNP | nCFD | KMT2C | L2653V | SNP | nCFD |
| ARID1A | G397R | SNP | nCFD | KMT2C | L291F | SNP | CFD |
| ARID1A | G444S | SNP | nCFD | KMT2C | L3311F | SNP | nCFD |
| ARID1A | G864S | SNP | nCFD | KMT2C | L3589F | SNP | nCFD |
| ARID1A | I1173F | SNP | nCFD | KMT2C | L3589V | SNP | nCFD |
| ARID1A | I692V | SNP | nCFD | KMT2C | L4143F | SNP | nCFD |
| ARID1A | K1795N | SNP | nCFD | KMT2C | L732F | SNP | nCFD |
| ARID1A | K1795R | SNP | nCFD | KMT2C | M1140I | SNP | nCFD |
| ARID1A | L1831V | SNP | nCFD | KMT2C | M1819T | SNP | nCFD |
| ARID1A | M1036I | SNP | CFD | KMT2C | M1895V | SNP | nCFD |
| ARID1A | M872T | SNP | nCFD | KMT2C | M3032T | SNP | nCFD |
| ARID1A | M961L | SNP | nCFD | KMT2C | M3275V | SNP | nCFD |
| ARID1A | N1313S | SNP | nCFD | KMT2C | M3329I | SNP | nCFD |
| ARID1A | N1705S | SNP | nCFD | KMT2C | M555I | SNP | nCFD |
| ARID1A | N1986S | SNP | CFD | KMT2C | M689V | SNP | nCFD |
| ARID1A | N2160D | SNP | CFD | KMT2C | M741T | SNP | nCFD |
| ARID1A | N2220S | SNP | CFD | KMT2C | N1118S | SNP | nCFD |
| ARID1A | P1175L | SNP | nCFD | KMT2C | N1385S | SNP | nCFD |
| ARID1A | P120S | SNP | nCFD | KMT2C | N1601S | SNP | nCFD |
| ARID1A | P1244S | SNP | nCFD | KMT2C | N2532S | SNP | nCFD |
| ARID1A | P1467R | SNP | nCFD | KMT2C | N2830D | SNP | nCFD |
| ARID1A | P158S | SNP | nCFD | KMT2C | N2924D | SNP | nCFD |
| ARID1A | P1771S | SNP | nCFD | KMT2C | N3338S | SNP | nCFD |
| ARID1A | P1771T | SNP | nCFD | KMT2C | N3505D | SNP | nCFD |
| ARID1A | P1897A | SNP | nCFD | KMT2C | N3505K | SNP | nCFD |
| ARID1A | P431R | SNP | nCFD | KMT2C | N3808D | SNP | nCFD |
| ARID1A | P580T | SNP | nCFD | KMT2C | N3808T | SNP | nCFD |
| ARID1A | Q1342P | SNP | nCFD | KMT2C | N3808Y | SNP | nCFD |
| ARID1A | Q1399L | SNP | nCFD | KMT2C | N452S | SNP | nCFD |
| ARID1A | Q1399P | SNP | nCFD | KMT2C | N4593S | SNP | CFD |

| | | | | | | | |
|---|---|---|---|---|---|---|---|
| ARID1A | Q200H | SNP | nCFD | KMT2C | N4686S | SNP | CFD |
| ARID1A | Q2219H | SNP | CFD | KMT2C | N567S | SNP | nCFD |
| ARID1A | Q583R | SNP | nCFD | KMT2C | N567T | SNP | nCFD |
| ARID1A | Q708P | SNP | nCFD | KMT2C | N621K | SNP | nCFD |
| ARID1A | R1749S | SNP | nCFD | KMT2C | N729D | SNP | nCFD |
| ARID1A | S1123C | SNP | nCFD | KMT2C | P1138S | SNP | nCFD |
| ARID1A | S1839C | SNP | nCFD | KMT2C | P1468T | SNP | nCFD |
| ARID1A | S1839G | SNP | nCFD | KMT2C | P157S | SNP | nCFD |
| ARID1A | S1944G | SNP | nCFD | KMT2C | P1669R | SNP | nCFD |
| ARID1A | S2211G | SNP | CFD | KMT2C | P1863A | SNP | nCFD |
| ARID1A | T1302A | SNP | nCFD | KMT2C | P189L | SNP | nCFD |
| ARID1A | T1302S | SNP | nCFD | KMT2C | P2093L | SNP | nCFD |
| ARID1A | T1743K | SNP | nCFD | KMT2C | P2093Q | SNP | nCFD |
| ARID1A | T1743M | SNP | nCFD | KMT2C | P2193L | SNP | nCFD |
| ARID1A | T1908P | SNP | nCFD | KMT2C | P2276S | SNP | nCFD |
| ARID1A | T290P | SNP | nCFD | KMT2C | P22S | SNP | nCFD |
| ARID1A | V1391M | SNP | nCFD | KMT2C | P2412T | SNP | nCFD |
| ARID1A | V1672G | SNP | nCFD | KMT2C | P2468S | SNP | nCFD |
| ARID1A | Y1226C | SNP | nCFD | KMT2C | P2600A | SNP | nCFD |
| ARID1A | Y1435C | SNP | nCFD | KMT2C | P2602R | SNP | nCFD |
| ARID1A | Y592C | SNP | nCFD | KMT2C | P2681L | SNP | nCFD |
| ASXL1 | A1041T | MUT | nCFD | KMT2C | P2780S | SNP | nCFD |
| ASXL1 | A1311T | MUT | nCFD | KMT2C | P2947S | SNP | nCFD |
| ASXL1 | A1357T | MUT | nCFD | KMT2C | P2960T | SNP | nCFD |
| ASXL1 | A621S | MUT | nCFD | KMT2C | P3367S | SNP | nCFD |
| ASXL1 | A761T | MUT | nCFD | KMT2C | P3468T | SNP | nCFD |
| ASXL1 | D1127N | MUT | nCFD | KMT2C | P3633S | SNP | nCFD |
| ASXL1 | E1102D | MUT | nCFD | KMT2C | P4059S | SNP | nCFD |
| ASXL1 | G1397S | MUT | nCFD | KMT2C | P4273S | SNP | nCFD |
| ASXL1 | G652S | MUT | nCFD | KMT2C | P4302A | SNP | nCFD |
| ASXL1 | H1126R | MUT | nCFD | KMT2C | P4302T | SNP | nCFD |
| ASXL1 | I919V | MUT | nCFD | KMT2C | P4374S | SNP | nCFD |
| ASXL1 | L1173S | MUT | nCFD | KMT2C | P4416S | SNP | CFD |
| ASXL1 | N1047Y | MUT | nCFD | KMT2C | P468A | SNP | nCFD |
| ASXL1 | N496K | MUT | nCFD | KMT2C | P468T | SNP | nCFD |
| ASXL1 | P1330S | MUT | nCFD | KMT2C | P837A | SNP | nCFD |
| ASXL1 | P808H | MUT | nCFD | KMT2C | Q2147H | SNP | nCFD |
| ASXL1 | R1190K | MUT | nCFD | KMT2C | Q240E | SNP | nCFD |
| ASXL1 | S51F | MUT | CFD | KMT2C | Q3407P | SNP | nCFD |
| ASXL1 | S846N | MUT | nCFD | KMT2C | Q3478E | SNP | nCFD |
| ASXL1 | T683A | MUT | nCFD | KMT2C | Q3481H | SNP | nCFD |
| ASXL1 | V375M | MUT | nCFD | KMT2C | Q3486R | SNP | nCFD |
| ASXL1 | A1071V | SNP | nCFD | KMT2C | Q3587E | SNP | nCFD |
| ASXL1 | A1312V | SNP | nCFD | KMT2C | Q3591H | SNP | nCFD |
| ASXL1 | C1204W | SNP | nCFD | KMT2C | Q3654E | SNP | nCFD |
| ASXL1 | C687G | SNP | nCFD | KMT2C | Q3696E | SNP | nCFD |
| ASXL1 | C687R | SNP | nCFD | KMT2C | Q3836K | SNP | nCFD |
| ASXL1 | D1017A | SNP | nCFD | KMT2C | Q448H | SNP | nCFD |
| ASXL1 | D1127E | SNP | nCFD | KMT2C | R1906Q | SNP | nCFD |
| ASXL1 | D1163N | SNP | nCFD | KMT2C | R1916G | SNP | nCFD |
| ASXL1 | D1252G | SNP | nCFD | KMT2C | R196T | SNP | nCFD |
| ASXL1 | D1252N | SNP | nCFD | KMT2C | R1986Q | SNP | nCFD |
| ASXL1 | D741V | SNP | nCFD | KMT2C | R2013K | SNP | nCFD |
| ASXL1 | D799Y | SNP | nCFD | KMT2C | R2463H | SNP | nCFD |
| ASXL1 | E1033V | SNP | nCFD | KMT2C | R2497H | SNP | nCFD |
| ASXL1 | E1383K | SNP | nCFD | KMT2C | R2497L | SNP | nCFD |
| ASXL1 | E477Q | SNP | nCFD | KMT2C | R2596Q | SNP | nCFD |
| ASXL1 | E865K | SNP | nCFD | KMT2C | R284Q | SNP | CFD |

| | | | | | | | |
|---|---|---|---|---|---|---|---|
| ASXL1 | F81S | SNP | CFD | KMT2C | R2963C | SNP | nCFD |
| ASXL1 | G1026E | SNP | nCFD | KMT2C | R3077H | SNP | nCFD |
| ASXL1 | G1154R | SNP | nCFD | KMT2C | R3350K | SNP | nCFD |
| ASXL1 | G1375W | SNP | nCFD | KMT2C | R3403C | SNP | nCFD |
| ASXL1 | G643A | SNP | nCFD | KMT2C | R4017S | SNP | nCFD |
| ASXL1 | G643E | SNP | nCFD | KMT2C | R4145C | SNP | nCFD |
| ASXL1 | G643V | SNP | nCFD | KMT2C | R4145H | SNP | nCFD |
| ASXL1 | G653R | SNP | nCFD | KMT2C | R4145L | SNP | nCFD |
| ASXL1 | G987R | SNP | nCFD | KMT2C | R4162L | SNP | nCFD |
| ASXL1 | H1524Y | SNP | CFD | KMT2C | R4162Q | SNP | nCFD |
| ASXL1 | H633R | SNP | nCFD | KMT2C | R4162W | SNP | nCFD |
| ASXL1 | I268F | SNP | CFD | KMT2C | R4334Q | SNP | nCFD |
| ASXL1 | I268V | SNP | CFD | KMT2C | R4597C | SNP | CFD |
| ASXL1 | I507N | SNP | nCFD | KMT2C | R4608H | SNP | CFD |
| ASXL1 | I552V | SNP | nCFD | KMT2C | R4673C | SNP | CFD |
| ASXL1 | K1157E | SNP | nCFD | KMT2C | R526H | SNP | nCFD |
| ASXL1 | K838R | SNP | nCFD | KMT2C | R526P | SNP | nCFD |
| ASXL1 | L1325F | SNP | nCFD | KMT2C | R841L | SNP | nCFD |
| ASXL1 | L1490F | SNP | CFD | KMT2C | R841Q | SNP | nCFD |
| ASXL1 | L386F | SNP | nCFD | KMT2C | R841W | SNP | nCFD |
| ASXL1 | M1249V | SNP | nCFD | KMT2C | R886H | SNP | nCFD |
| ASXL1 | M168L | SNP | nCFD | KMT2C | R894Q | SNP | nCFD |
| ASXL1 | N986S | SNP | nCFD | KMT2C | R909G | SNP | nCFD |
| ASXL1 | P1259L | SNP | nCFD | KMT2C | R909K | SNP | nCFD |
| ASXL1 | P1330L | SNP | nCFD | KMT2C | S1148L | SNP | nCFD |
| ASXL1 | P1340T | SNP | nCFD | KMT2C | S1449A | SNP | nCFD |
| ASXL1 | P1358A | SNP | nCFD | KMT2C | S1724I | SNP | nCFD |
| ASXL1 | P570L | SNP | nCFD | KMT2C | S1733L | SNP | nCFD |
| ASXL1 | P779L | SNP | nCFD | KMT2C | S1836N | SNP | nCFD |
| ASXL1 | Q372K | SNP | nCFD | KMT2C | S1860C | SNP | nCFD |
| ASXL1 | Q89R | SNP | nCFD | KMT2C | S1931L | SNP | nCFD |
| ASXL1 | R1224T | SNP | nCFD | KMT2C | S2025T | SNP | nCFD |
| ASXL1 | R1247C | SNP | nCFD | KMT2C | S210N | SNP | nCFD |
| ASXL1 | R271P | SNP | CFD | KMT2C | S2308C | SNP | nCFD |
| ASXL1 | R394C | SNP | nCFD | KMT2C | S2897F | SNP | nCFD |
| ASXL1 | R394H | SNP | nCFD | KMT2C | S2984F | SNP | nCFD |
| ASXL1 | R499H | SNP | nCFD | KMT2C | S2L | SNP | nCFD |
| ASXL1 | R620C | SNP | nCFD | KMT2C | S338L | SNP | nCFD |
| ASXL1 | R625Q | SNP | nCFD | KMT2C | S3547P | SNP | nCFD |
| ASXL1 | S1166R | SNP | nCFD | KMT2C | S3660L | SNP | nCFD |
| ASXL1 | S1212F | SNP | nCFD | KMT2C | S4300A | SNP | nCFD |
| ASXL1 | S1231F | SNP | nCFD | KMT2C | S4300P | SNP | nCFD |
| ASXL1 | S1428P | SNP | nCFD | KMT2C | S730C | SNP | nCFD |
| ASXL1 | S370T | SNP | nCFD | KMT2C | S764F | SNP | nCFD |
| ASXL1 | S412F | SNP | nCFD | KMT2C | S772L | SNP | nCFD |
| ASXL1 | T1010M | SNP | nCFD | KMT2C | S793L | SNP | nCFD |
| ASXL1 | T1010R | SNP | nCFD | KMT2C | S888T | SNP | nCFD |
| ASXL1 | T1221K | SNP | nCFD | KMT2C | T1358A | SNP | nCFD |
| ASXL1 | T1372S | SNP | nCFD | KMT2C | T1621I | SNP | nCFD |
| ASXL1 | V1069I | SNP | nCFD | KMT2C | T1636P | SNP | nCFD |
| ASXL1 | V1216L | SNP | nCFD | KMT2C | T2008A | SNP | nCFD |
| ASXL1 | V1297I | SNP | nCFD | KMT2C | T3017K | SNP | nCFD |
| ASXL1 | V807I | SNP | nCFD | KMT2C | T3017S | SNP | nCFD |
| ASXL1 | V907I | SNP | nCFD | KMT2C | T316S | SNP | CFD |
| ATM | A2062V | MUT | nCFD | KMT2C | T3317I | SNP | nCFD |
| ATM | A2308T | MUT | CFD | KMT2C | T3317K | SNP | nCFD |
| ATM | A2524S | MUT | nCFD | KMT2C | T3317R | SNP | nCFD |
| ATM | A2626V | MUT | nCFD | KMT2C | T3586I | SNP | nCFD |

| | | | | | | | |
|---|---|---|---|---|---|---|---|
| ATM | A3006T | MUT | nCFD | KMT2C | T3857M | SNP | nCFD |
| ATM | A350T | MUT | nCFD | KMT2C | T3884M | SNP | nCFD |
| ATM | C107F | MUT | CFD | KMT2C | T4688A | SNP | CFD |
| ATM | C977S | MUT | nCFD | KMT2C | T4688P | SNP | CFD |
| ATM | D1682H | MUT | nCFD | KMT2C | T61A | SNP | nCFD |
| ATM | D1781H | MUT | nCFD | KMT2C | V1163L | SNP | nCFD |
| ATM | D1930V | MUT | nCFD | KMT2C | V2171I | SNP | nCFD |
| ATM | D2395V | MUT | CFD | KMT2C | V2428A | SNP | nCFD |
| ATM | D2448A | MUT | CFD | KMT2C | V262A | SNP | CFD |
| ATM | D2725E | MUT | CFD | KMT2C | V3102L | SNP | nCFD |
| ATM | D2725G | MUT | CFD | KMT2C | V3811I | SNP | nCFD |
| ATM | D479Y | MUT | nCFD | KMT2C | V4644G | SNP | CFD |
| ATM | E1959K | MUT | nCFD | KMT2C | V4668I | SNP | CFD |
| ATM | E2039G | MUT | nCFD | KMT2C | V634L | SNP | nCFD |
| ATM | E2164Q | MUT | CFD | KMT2C | V700L | SNP | nCFD |
| ATM | E28K | MUT | CFD | KMT2C | V919L | SNP | nCFD |
| ATM | F1025L | MUT | nCFD | KMT2C | V920A | SNP | nCFD |
| ATM | F2393I | MUT | CFD | KMT2C | W4352L | SNP | nCFD |
| ATM | F2732V | MUT | CFD | KMT2C | Y2218S | SNP | nCFD |
| ATM | F2827L | MUT | CFD | KMT2C | Y2466C | SNP | nCFD |
| ATM | F858S | MUT | nCFD | KMT2C | Y3699C | SNP | nCFD |
| ATM | G1522R | MUT | nCFD | KMT2C | Y4774H | SNP | nCFD |
| ATM | G2063E | MUT | nCFD | KMT2C | Y987H | SNP | nCFD |
| ATM | G2072R | MUT | nCFD | KMT2D | A1788T | MUT | nCFD |
| ATM | G2694R | MUT | nCFD | KMT2D | A1841V | MUT | nCFD |
| ATM | G2695A | MUT | nCFD | KMT2D | A3552G | MUT | nCFD |
| ATM | G2695V | MUT | nCFD | KMT2D | A3593E | MUT | nCFD |
| ATM | H2038R | MUT | nCFD | KMT2D | A3678V | MUT | nCFD |
| ATM | H2125R | MUT | CFD | KMT2D | A4760V | MUT | nCFD |
| ATM | I190K | MUT | nCFD | KMT2D | A4862G | MUT | nCFD |
| ATM | I2311F | MUT | CFD | KMT2D | A4908V | MUT | nCFD |
| ATM | I2888T | MUT | CFD | KMT2D | A4926T | MUT | nCFD |
| ATM | I352T | MUT | nCFD | KMT2D | A5121V | MUT | CFD |
| ATM | K2657N | MUT | nCFD | KMT2D | A5187P | MUT | CFD |
| ATM | K2687E | MUT | nCFD | KMT2D | A5272P | MUT | CFD |
| ATM | K2717M | MUT | CFD | KMT2D | C1383Y | MUT | nCFD |
| ATM | K3018N | MUT | nCFD | KMT2D | C1430F | MUT | CFD |
| ATM | L1206V | MUT | nCFD | KMT2D | C1456Y | MUT | CFD |
| ATM | L120R | MUT | CFD | KMT2D | C214Y | MUT | CFD |
| ATM | L2033V | MUT | nCFD | KMT2D | C5338R | MUT | nCFD |
| ATM | L2293P | MUT | CFD | KMT2D | D1343V | MUT | nCFD |
| ATM | L2416P | MUT | CFD | KMT2D | D2769N | MUT | nCFD |
| ATM | L2427P | MUT | CFD | KMT2D | D5257V | MUT | CFD |
| ATM | L2427R | MUT | CFD | KMT2D | E226G | MUT | nCFD |
| ATM | L2445P | MUT | CFD | KMT2D | E436K | MUT | nCFD |
| ATM | L2447W | MUT | CFD | KMT2D | F1790L | MUT | nCFD |
| ATM | L2490F | MUT | nCFD | KMT2D | F301C | MUT | CFD |
| ATM | L2780H | MUT | CFD | KMT2D | G1234E | MUT | nCFD |
| ATM | L2890V | MUT | CFD | KMT2D | G1255D | MUT | nCFD |
| ATM | L2945M | MUT | CFD | KMT2D | G1434D | MUT | CFD |
| ATM | L2952V | MUT | CFD | KMT2D | G1808E | MUT | nCFD |
| ATM | M2405L | MUT | CFD | KMT2D | G3095D | MUT | nCFD |
| ATM | M2616I | MUT | nCFD | KMT2D | G3095S | MUT | nCFD |
| ATM | M3011K | MUT | nCFD | KMT2D | G4120D | MUT | nCFD |
| ATM | N1081S | MUT | nCFD | KMT2D | G4593E | MUT | nCFD |
| ATM | N1855S | MUT | nCFD | KMT2D | G4716R | MUT | nCFD |
| ATM | N2875S | MUT | CFD | KMT2D | G5410E | MUT | CFD |
| ATM | P1069S | MUT | nCFD | KMT2D | H1405R | MUT | nCFD |

| | | | | | | | |
|---|---|---|---|---|---|---|---|
| ATM | P2353S | MUT | CFD | KMT2D | H1453R | MUT | CFD |
| ATM | P2699S | MUT | nCFD | KMT2D | K1412R | MUT | nCFD |
| ATM | P604S | MUT | nCFD | KMT2D | L2610P | MUT | nCFD |
| ATM | Q2522H | MUT | nCFD | KMT2D | L303P | MUT | CFD |
| ATM | Q2730R | MUT | CFD | KMT2D | L4075F | MUT | nCFD |
| ATM | Q984E | MUT | nCFD | KMT2D | L4786P | MUT | nCFD |
| ATM | R2034P | MUT | nCFD | KMT2D | L5293H | MUT | CFD |
| ATM | R2263S | MUT | CFD | KMT2D | L5411H | MUT | CFD |
| ATM | R2486G | MUT | CFD | KMT2D | M3777R | MUT | nCFD |
| ATM | R248Q | MUT | nCFD | KMT2D | P1135S | MUT | nCFD |
| ATM | R2526S | MUT | nCFD | KMT2D | P1170L | MUT | nCFD |
| ATM | R3008C | MUT | nCFD | KMT2D | P1669L | MUT | nCFD |
| ATM | R3008H | MUT | nCFD | KMT2D | P1984L | MUT | nCFD |
| ATM | R981C | MUT | nCFD | KMT2D | P2781S | MUT | nCFD |
| ATM | S1863F | MUT | nCFD | KMT2D | P2930S | MUT | nCFD |
| ATM | S2165F | MUT | CFD | KMT2D | P3100R | MUT | nCFD |
| ATM | S2489F | MUT | CFD | KMT2D | P3289L | MUT | nCFD |
| ATM | S2859F | MUT | CFD | KMT2D | P4163S | MUT | nCFD |
| ATM | S496R | MUT | nCFD | KMT2D | P4631T | MUT | nCFD |
| ATM | S601I | MUT | nCFD | KMT2D | Q3950R | MUT | nCFD |
| ATM | T2113A | MUT | CFD | KMT2D | Q4228E | MUT | nCFD |
| ATM | T2666A | MUT | nCFD | KMT2D | R1388L | MUT | nCFD |
| ATM | V2441F | MUT | CFD | KMT2D | R2635Q | MUT | nCFD |
| ATM | V2727A | MUT | CFD | KMT2D | R3127H | MUT | nCFD |
| ATM | W1221R | MUT | nCFD | KMT2D | R3656H | MUT | nCFD |
| ATM | W2769L | MUT | CFD | KMT2D | R4484Q | MUT | nCFD |
| ATM | Y1216H | MUT | nCFD | KMT2D | R4659P | MUT | nCFD |
| ATM | Y1470C | MUT | nCFD | KMT2D | R4825Q | MUT | nCFD |
| ATM | Y2019F | MUT | nCFD | KMT2D | R5021Q | MUT | nCFD |
| ATM | Y2437D | MUT | CFD | KMT2D | R5027Q | MUT | nCFD |
| ATM | Y2437S | MUT | CFD | KMT2D | R5048C | MUT | CFD |
| ATM | Y2954C | MUT | CFD | KMT2D | R5153Q | MUT | nCFD |
| ATM | Y332C | MUT | nCFD | KMT2D | R5179C | MUT | nCFD |
| ATM | A112T | SNP | CFD | KMT2D | R5179H | MUT | nCFD |
| ATM | A1309T | SNP | nCFD | KMT2D | R5179S | MUT | nCFD |
| ATM | A1427T | SNP | nCFD | KMT2D | R5214C | MUT | CFD |
| ATM | A216P | SNP | nCFD | KMT2D | R5225C | MUT | CFD |
| ATM | A216S | SNP | nCFD | KMT2D | R5390W | MUT | nCFD |
| ATM | A2274S | SNP | CFD | KMT2D | R5392H | MUT | nCFD |
| ATM | A2274T | SNP | CFD | KMT2D | R5432W | MUT | CFD |
| ATM | A235T | SNP | nCFD | KMT2D | S1335F | MUT | nCFD |
| ATM | A799V | SNP | nCFD | KMT2D | S2970I | MUT | nCFD |
| ATM | C107Y | SNP | CFD | KMT2D | T1506S | MUT | nCFD |
| ATM | C2464R | SNP | CFD | KMT2D | T2441S | MUT | nCFD |
| ATM | C532Y | SNP | nCFD | KMT2D | T3188A | MUT | nCFD |
| ATM | D1080E | SNP | nCFD | KMT2D | V4642I | MUT | nCFD |
| ATM | D126E | SNP | CFD | KMT2D | V4799M | MUT | nCFD |
| ATM | D1278E | SNP | nCFD | KMT2D | Y5216S | MUT | CFD |
| ATM | D1853N | SNP | nCFD | KMT2D | Y5317C | MUT | CFD |
| ATM | D1853V | SNP | nCFD | KMT2D | A1130V | SNP | nCFD |
| ATM | D44G | SNP | CFD | KMT2D | A1565V | SNP | nCFD |
| ATM | E1169K | SNP | nCFD | KMT2D | A1716V | SNP | nCFD |
| ATM | E119K | SNP | CFD | KMT2D | A2434D | SNP | nCFD |
| ATM | E1313Q | SNP | nCFD | KMT2D | A2657V | SNP | nCFD |
| ATM | E2990K | SNP | nCFD | KMT2D | A2925V | SNP | nCFD |
| ATM | E347Q | SNP | nCFD | KMT2D | A3183P | SNP | nCFD |
| ATM | E347V | SNP | nCFD | KMT2D | A352V | SNP | nCFD |
| ATM | E699G | SNP | nCFD | KMT2D | A4361T | SNP | nCFD |

| | | | | | | | |
|---|---|---|---|---|---|---|---|
| ATM | E708K | SNP | nCFD | KMT2D | A4594P | SNP | nCFD |
| ATM | F1463C | SNP | nCFD | KMT2D | A4599V | SNP | nCFD |
| ATM | F3002V | SNP | nCFD | KMT2D | A4655V | SNP | nCFD |
| ATM | F582L | SNP | nCFD | KMT2D | A476T | SNP | nCFD |
| ATM | F627C | SNP | nCFD | KMT2D | A4965T | SNP | nCFD |
| ATM | F763L | SNP | nCFD | KMT2D | A765P | SNP | nCFD |
| ATM | G1307R | SNP | nCFD | KMT2D | C239R | SNP | nCFD |
| ATM | G2023R | SNP | nCFD | KMT2D | C323Y | SNP | CFD |
| ATM | G2180V | SNP | CFD | KMT2D | D1646Y | SNP | nCFD |
| ATM | G2709S | SNP | nCFD | KMT2D | D1825N | SNP | nCFD |
| ATM | G514D | SNP | nCFD | KMT2D | D2066G | SNP | nCFD |
| ATM | G833A | SNP | nCFD | KMT2D | D2092N | SNP | nCFD |
| ATM | H1380Y | SNP | nCFD | KMT2D | D3419G | SNP | nCFD |
| ATM | H1436Y | SNP | nCFD | KMT2D | D4861N | SNP | nCFD |
| ATM | H42R | SNP | CFD | KMT2D | D632E | SNP | nCFD |
| ATM | H674R | SNP | nCFD | KMT2D | E1549D | SNP | nCFD |
| ATM | H996Q | SNP | nCFD | KMT2D | E1663K | SNP | nCFD |
| ATM | I124V | SNP | CFD | KMT2D | E446A | SNP | nCFD |
| ATM | I1422V | SNP | nCFD | KMT2D | E446G | SNP | nCFD |
| ATM | I1547V | SNP | nCFD | KMT2D | E4805K | SNP | nCFD |
| ATM | I1688T | SNP | nCFD | KMT2D | E4939K | SNP | nCFD |
| ATM | I2030V | SNP | nCFD | KMT2D | E913K | SNP | nCFD |
| ATM | I709M | SNP | nCFD | KMT2D | F1961L | SNP | nCFD |
| ATM | I879V | SNP | nCFD | KMT2D | F2566L | SNP | nCFD |
| ATM | K1454N | SNP | nCFD | KMT2D | F3515V | SNP | nCFD |
| ATM | K1992T | SNP | nCFD | KMT2D | F372Y | SNP | nCFD |
| ATM | K482Q | SNP | nCFD | KMT2D | G1286R | SNP | nCFD |
| ATM | K810E | SNP | nCFD | KMT2D | G1323D | SNP | nCFD |
| ATM | K92T | SNP | CFD | KMT2D | G2279E | SNP | nCFD |
| ATM | L1420F | SNP | nCFD | KMT2D | G2493E | SNP | nCFD |
| ATM | L1541F | SNP | nCFD | KMT2D | G2569S | SNP | nCFD |
| ATM | L2307F | SNP | CFD | KMT2D | G3169V | SNP | nCFD |
| ATM | L2330V | SNP | CFD | KMT2D | G3324V | SNP | nCFD |
| ATM | L2332P | SNP | CFD | KMT2D | G3366S | SNP | nCFD |
| ATM | L2332R | SNP | CFD | KMT2D | G3694V | SNP | nCFD |
| ATM | L2965F | SNP | nCFD | KMT2D | G3819D | SNP | nCFD |
| ATM | L432Q | SNP | nCFD | KMT2D | G4189A | SNP | nCFD |
| ATM | L546V | SNP | nCFD | KMT2D | G4373S | SNP | nCFD |
| ATM | L942F | SNP | nCFD | KMT2D | G4489R | SNP | nCFD |
| ATM | M1040V | SNP | nCFD | KMT2D | G4833R | SNP | nCFD |
| ATM | M1210T | SNP | nCFD | KMT2D | G4971D | SNP | nCFD |
| ATM | M1321I | SNP | nCFD | KMT2D | G4971V | SNP | nCFD |
| ATM | M1909T | SNP | nCFD | KMT2D | H769P | SNP | nCFD |
| ATM | M2224V | SNP | CFD | KMT2D | I1344V | SNP | nCFD |
| ATM | M963V | SNP | nCFD | KMT2D | I1509V | SNP | nCFD |
| ATM | N1005S | SNP | nCFD | KMT2D | I2026V | SNP | nCFD |
| ATM | N1094S | SNP | nCFD | KMT2D | I5523T | SNP | nCFD |
| ATM | N1356D | SNP | nCFD | KMT2D | K2548E | SNP | nCFD |
| ATM | N1431S | SNP | nCFD | KMT2D | K287E | SNP | CFD |
| ATM | N1477K | SNP | nCFD | KMT2D | K4416R | SNP | nCFD |
| ATM | N1650S | SNP | nCFD | KMT2D | K4494T | SNP | nCFD |
| ATM | N1650T | SNP | nCFD | KMT2D | L1599F | SNP | nCFD |
| ATM | N2501S | SNP | nCFD | KMT2D | L2245V | SNP | nCFD |
| ATM | N358S | SNP | nCFD | KMT2D | L2398V | SNP | nCFD |
| ATM | N504S | SNP | nCFD | KMT2D | L3367F | SNP | nCFD |
| ATM | N870D | SNP | nCFD | KMT2D | L3619R | SNP | nCFD |
| ATM | P1054H | SNP | nCFD | KMT2D | L474S | SNP | nCFD |
| ATM | P1054R | SNP | nCFD | KMT2D | M1098I | SNP | nCFD |

| | | | | | | | |
|---|---|---|---|---|---|---|---|
| ATM | P1480L | SNP | nCFD | KMT2D | M1417I | SNP | nCFD |
| ATM | P2974L | SNP | nCFD | KMT2D | M2652L | SNP | nCFD |
| ATM | Q1128R | SNP | nCFD | KMT2D | M307R | SNP | CFD |
| ATM | Q1982L | SNP | nCFD | KMT2D | M3161T | SNP | nCFD |
| ATM | Q1982R | SNP | nCFD | KMT2D | M3349V | SNP | nCFD |
| ATM | R1086C | SNP | nCFD | KMT2D | M3777I | SNP | nCFD |
| ATM | R114K | SNP | CFD | KMT2D | M3870I | SNP | nCFD |
| ATM | R1150T | SNP | nCFD | KMT2D | M5029L | SNP | nCFD |
| ATM | R1489H | SNP | nCFD | KMT2D | M5135V | SNP | CFD |
| ATM | R1489L | SNP | nCFD | KMT2D | M635V | SNP | nCFD |
| ATM | R1898Q | SNP | nCFD | KMT2D | M716T | SNP | nCFD |
| ATM | R1918T | SNP | nCFD | KMT2D | N106S | SNP | nCFD |
| ATM | R2034Q | SNP | nCFD | KMT2D | N2085K | SNP | nCFD |
| ATM | R2392W | SNP | CFD | KMT2D | N2965S | SNP | nCFD |
| ATM | R2461C | SNP | CFD | KMT2D | N2965T | SNP | nCFD |
| ATM | R2461S | SNP | CFD | KMT2D | P1039S | SNP | nCFD |
| ATM | R2580S | SNP | nCFD | KMT2D | P1191L | SNP | nCFD |
| ATM | R2691C | SNP | nCFD | KMT2D | P2100L | SNP | nCFD |
| ATM | R2719H | SNP | CFD | KMT2D | P2100Q | SNP | nCFD |
| ATM | R2719L | SNP | CFD | KMT2D | P2129S | SNP | nCFD |
| ATM | R2748T | SNP | CFD | KMT2D | P2145L | SNP | nCFD |
| ATM | R2832H | SNP | CFD | KMT2D | P2210L | SNP | nCFD |
| ATM | R337H | SNP | nCFD | KMT2D | P2271S | SNP | nCFD |
| ATM | R451C | SNP | nCFD | KMT2D | P2349L | SNP | nCFD |
| ATM | R451H | SNP | nCFD | KMT2D | P2352L | SNP | nCFD |
| ATM | R45W | SNP | CFD | KMT2D | P2354L | SNP | nCFD |
| ATM | R568I | SNP | nCFD | KMT2D | P2387L | SNP | nCFD |
| ATM | R568K | SNP | nCFD | KMT2D | P2400A | SNP | nCFD |
| ATM | S1455R | SNP | nCFD | KMT2D | P2407L | SNP | nCFD |
| ATM | S1691R | SNP | nCFD | KMT2D | P2545A | SNP | nCFD |
| ATM | S1983N | SNP | nCFD | KMT2D | P2557L | SNP | nCFD |
| ATM | S2146T | SNP | CFD | KMT2D | P2717S | SNP | nCFD |
| ATM | S2168L | SNP | CFD | KMT2D | P2938L | SNP | nCFD |
| ATM | S2168W | SNP | CFD | KMT2D | P3129S | SNP | nCFD |
| ATM | S333F | SNP | nCFD | KMT2D | P3369S | SNP | nCFD |
| ATM | S49C | SNP | CFD | KMT2D | P3490L | SNP | nCFD |
| ATM | S49F | SNP | CFD | KMT2D | P3665A | SNP | nCFD |
| ATM | S707P | SNP | nCFD | KMT2D | P367L | SNP | nCFD |
| ATM | S788R | SNP | nCFD | KMT2D | P3695S | SNP | nCFD |
| ATM | S978P | SNP | nCFD | KMT2D | P374T | SNP | nCFD |
| ATM | S99G | SNP | CFD | KMT2D | P3794S | SNP | nCFD |
| ATM | T1100M | SNP | nCFD | KMT2D | P396R | SNP | nCFD |
| ATM | T1100R | SNP | nCFD | KMT2D | P4048L | SNP | nCFD |
| ATM | T1118A | SNP | nCFD | KMT2D | P4155T | SNP | nCFD |
| ATM | T1697A | SNP | nCFD | KMT2D | P439L | SNP | nCFD |
| ATM | T1871I | SNP | nCFD | KMT2D | P443Q | SNP | nCFD |
| ATM | T1880M | SNP | nCFD | KMT2D | P448R | SNP | nCFD |
| ATM | T1880R | SNP | nCFD | KMT2D | P460T | SNP | nCFD |
| ATM | T2059I | SNP | nCFD | KMT2D | P4916L | SNP | nCFD |
| ATM | T2113S | SNP | CFD | KMT2D | P5325T | SNP | nCFD |
| ATM | T2335I | SNP | CFD | KMT2D | P607L | SNP | nCFD |
| ATM | T2335K | SNP | CFD | KMT2D | P628L | SNP | nCFD |
| ATM | T2438I | SNP | CFD | KMT2D | P637T | SNP | nCFD |
| ATM | T2640I | SNP | nCFD | KMT2D | P647A | SNP | nCFD |
| ATM | T2640S | SNP | nCFD | KMT2D | P647T | SNP | nCFD |
| ATM | T452I | SNP | nCFD | KMT2D | P692T | SNP | nCFD |
| ATM | T761A | SNP | nCFD | KMT2D | P706S | SNP | nCFD |
| ATM | T761S | SNP | nCFD | KMT2D | P719L | SNP | nCFD |

| | | | | | | | |
|---|---|---|---|---|---|---|---|
| ATM | T935A | SNP | nCFD | KMT2D | P736S | SNP | nCFD |
| ATM | T935P | SNP | nCFD | KMT2D | P786A | SNP | nCFD |
| ATM | V1160L | SNP | nCFD | KMT2D | P813L | SNP | nCFD |
| ATM | V1160M | SNP | nCFD | KMT2D | P826H | SNP | nCFD |
| ATM | V1570A | SNP | nCFD | KMT2D | P826R | SNP | nCFD |
| ATM | V1729F | SNP | nCFD | KMT2D | P859R | SNP | nCFD |
| ATM | V1729L | SNP | nCFD | KMT2D | P859S | SNP | nCFD |
| ATM | V182L | SNP | nCFD | KMT2D | P877S | SNP | nCFD |
| ATM | V2079I | SNP | nCFD | KMT2D | P877T | SNP | nCFD |
| ATM | V2540I | SNP | nCFD | KMT2D | P881L | SNP | nCFD |
| ATM | V2757G | SNP | CFD | KMT2D | P886A | SNP | nCFD |
| ATM | V341I | SNP | nCFD | KMT2D | P886T | SNP | nCFD |
| ATM | V410A | SNP | nCFD | KMT2D | P949L | SNP | nCFD |
| ATM | V519A | SNP | nCFD | KMT2D | P972L | SNP | nCFD |
| ATM | V976A | SNP | nCFD | KMT2D | P998A | SNP | nCFD |
| ATM | V998M | SNP | nCFD | KMT2D | P998S | SNP | nCFD |
| ATM | W2638G | SNP | nCFD | KMT2D | P998T | SNP | nCFD |
| ATM | Y1300C | SNP | nCFD | KMT2D | Q3489H | SNP | nCFD |
| ATM | Y137F | SNP | CFD | KMT2D | Q3738R | SNP | nCFD |
| ATM | Y1475C | SNP | nCFD | KMT2D | Q4007K | SNP | nCFD |
| ATM | Y1961C | SNP | nCFD | KMT2D | R1189C | SNP | nCFD |
| ATM | Y2009H | SNP | nCFD | KMT2D | R1313Q | SNP | nCFD |
| ATRX | A1804V | MUT | CFD | KMT2D | R1814G | SNP | nCFD |
| ATRX | A1868P | MUT | CFD | KMT2D | R185C | SNP | CFD |
| ATRX | A1988D | MUT | nCFD | KMT2D | R191Q | SNP | CFD |
| ATRX | A2011T | MUT | nCFD | KMT2D | R2188H | SNP | nCFD |
| ATRX | A364T | MUT | nCFD | KMT2D | R2188L | SNP | nCFD |
| ATRX | C240G | MUT | nCFD | KMT2D | R2235K | SNP | nCFD |
| ATRX | C265Y | MUT | nCFD | KMT2D | R228C | SNP | nCFD |
| ATRX | D1925N | MUT | nCFD | KMT2D | R228G | SNP | nCFD |
| ATRX | D1949E | MUT | nCFD | KMT2D | R2443C | SNP | nCFD |
| ATRX | D1949Y | MUT | nCFD | KMT2D | R2460C | SNP | nCFD |
| ATRX | E1904V | MUT | nCFD | KMT2D | R2611C | SNP | nCFD |
| ATRX | E447K | MUT | nCFD | KMT2D | R2975C | SNP | nCFD |
| ATRX | E557K | MUT | nCFD | KMT2D | R3342C | SNP | nCFD |
| ATRX | E8D | MUT | nCFD | KMT2D | R3482W | SNP | nCFD |
| ATRX | F2210L | MUT | nCFD | KMT2D | R3596Q | SNP | nCFD |
| ATRX | F239L | MUT | nCFD | KMT2D | R3656C | SNP | nCFD |
| ATRX | F847Y | MUT | nCFD | KMT2D | R3714K | SNP | nCFD |
| ATRX | G175V | MUT | nCFD | KMT2D | R3727C | SNP | nCFD |
| ATRX | G2155V | MUT | CFD | KMT2D | R4238C | SNP | nCFD |
| ATRX | I1216V | MUT | nCFD | KMT2D | R4288Q | SNP | nCFD |
| ATRX | I353F | MUT | nCFD | KMT2D | R4288W | SNP | nCFD |
| ATRX | I85T | MUT | nCFD | KMT2D | R4420Q | SNP | nCFD |
| ATRX | K1361E | MUT | nCFD | KMT2D | R4420W | SNP | nCFD |
| ATRX | K2225N | MUT | nCFD | KMT2D | R4455C | SNP | nCFD |
| ATRX | K425Q | MUT | nCFD | KMT2D | R4478W | SNP | nCFD |
| ATRX | L1592H | MUT | CFD | KMT2D | R4659W | SNP | nCFD |
| ATRX | L1651H | MUT | CFD | KMT2D | R466C | SNP | nCFD |
| ATRX | L1708F | MUT | CFD | KMT2D | R4729Q | SNP | nCFD |
| ATRX | L192F | MUT | nCFD | KMT2D | R4825G | SNP | nCFD |
| ATRX | L924I | MUT | nCFD | KMT2D | R4825W | SNP | nCFD |
| ATRX | M828I | MUT | nCFD | KMT2D | R5224H | SNP | CFD |
| ATRX | N2125K | MUT | CFD | KMT2D | R5229H | SNP | CFD |
| ATRX | P144L | MUT | nCFD | KMT2D | R5229L | SNP | CFD |
| ATRX | Q219K | MUT | nCFD | KMT2D | R737W | SNP | nCFD |
| ATRX | Q2416H | MUT | nCFD | KMT2D | R746W | SNP | nCFD |
| ATRX | Q929E | MUT | nCFD | KMT2D | R83Q | SNP | nCFD |

| | | | | | | | |
|---|---|---|---|---|---|---|---|
| ATRX | R1022Q | MUT | nCFD | KMT2D | S2215T | SNP | nCFD |
| ATRX | R1302I | MUT | nCFD | KMT2D | S2251L | SNP | nCFD |
| ATRX | R1504L | MUT | nCFD | KMT2D | S2858A | SNP | nCFD |
| ATRX | R2028P | MUT | CFD | KMT2D | S4010P | SNP | nCFD |
| ATRX | R2346M | MUT | nCFD | KMT2D | S4327C | SNP | nCFD |
| ATRX | R246C | MUT | nCFD | KMT2D | S4907L | SNP | nCFD |
| ATRX | R840I | MUT | nCFD | KMT2D | S495C | SNP | nCFD |
| ATRX | S2365L | MUT | nCFD | KMT2D | S849L | SNP | nCFD |
| ATRX | T387A | MUT | nCFD | KMT2D | T1246M | SNP | nCFD |
| ATRX | V1834L | MUT | CFD | KMT2D | T1370A | SNP | nCFD |
| ATRX | V2162A | MUT | nCFD | KMT2D | T2137A | SNP | nCFD |
| ATRX | W1958G | MUT | nCFD | KMT2D | T2191M | SNP | nCFD |
| ATRX | A462T | SNP | nCFD | KMT2D | T2524A | SNP | nCFD |
| ATRX | A833V | SNP | nCFD | KMT2D | T261S | SNP | nCFD |
| ATRX | A891G | SNP | nCFD | KMT2D | T2959N | SNP | nCFD |
| ATRX | D705G | SNP | nCFD | KMT2D | T3221N | SNP | nCFD |
| ATRX | D709V | SNP | nCFD | KMT2D | T4368A | SNP | nCFD |
| ATRX | D975N | SNP | nCFD | KMT2D | T4368N | SNP | nCFD |
| ATRX | D99H | SNP | nCFD | KMT2D | T4852I | SNP | nCFD |
| ATRX | E2351D | SNP | nCFD | KMT2D | T944I | SNP | nCFD |
| ATRX | E822D | SNP | nCFD | KMT2D | V2786M | SNP | nCFD |
| ATRX | E884D | SNP | nCFD | KMT2D | V401M | SNP | nCFD |
| ATRX | F847S | SNP | nCFD | KMT2D | V4305I | SNP | nCFD |
| ATRX | G1085E | SNP | nCFD | KMT2D | V4407A | SNP | nCFD |
| ATRX | G1217R | SNP | nCFD | KMT2D | V4639M | SNP | nCFD |
| ATRX | G164V | SNP | nCFD | NSD1 | A1033T | MUT | nCFD |
| ATRX | G2489E | SNP | nCFD | NSD1 | A1260S | MUT | nCFD |
| ATRX | H1238Y | SNP | nCFD | NSD1 | A2247T | MUT | nCFD |
| ATRX | H475D | SNP | nCFD | NSD1 | A2323V | MUT | nCFD |
| ATRX | H475Y | SNP | nCFD | NSD1 | A376S | MUT | CFD |
| ATRX | H865Q | SNP | nCFD | NSD1 | A520T | MUT | nCFD |
| ATRX | I2291M | SNP | nCFD | NSD1 | A690T | MUT | nCFD |
| ATRX | I347M | SNP | nCFD | NSD1 | A735T | MUT | nCFD |
| ATRX | I565V | SNP | nCFD | NSD1 | C1733Y | MUT | nCFD |
| ATRX | I708M | SNP | nCFD | NSD1 | D2525N | MUT | nCFD |
| ATRX | I737V | SNP | nCFD | NSD1 | D659G | MUT | nCFD |
| ATRX | I901V | SNP | nCFD | NSD1 | D875E | MUT | nCFD |
| ATRX | K1176M | SNP | nCFD | NSD1 | E2053K | MUT | CFD |
| ATRX | K782R | SNP | nCFD | NSD1 | E724K | MUT | nCFD |
| ATRX | L464V | SNP | nCFD | NSD1 | F1110L | MUT | nCFD |
| ATRX | L98F | SNP | nCFD | NSD1 | F1633L | MUT | nCFD |
| ATRX | M216I | SNP | nCFD | NSD1 | G1364E | MUT | nCFD |
| ATRX | M2410T | SNP | nCFD | NSD1 | G1518C | MUT | nCFD |
| ATRX | M2479V | SNP | nCFD | NSD1 | G1656V | MUT | nCFD |
| ATRX | N1860S | SNP | CFD | NSD1 | G971D | MUT | nCFD |
| ATRX | N1860T | SNP | CFD | NSD1 | H515L | MUT | nCFD |
| ATRX | N237H | SNP | nCFD | NSD1 | H515R | MUT | nCFD |
| ATRX | P609A | SNP | nCFD | NSD1 | I636V | MUT | nCFD |
| ATRX | P667L | SNP | nCFD | NSD1 | K1121E | MUT | nCFD |
| ATRX | P717L | SNP | nCFD | NSD1 | L2329R | MUT | nCFD |
| ATRX | P717R | SNP | nCFD | NSD1 | L7P | MUT | nCFD |
| ATRX | Q545E | SNP | nCFD | NSD1 | M2456T | MUT | nCFD |
| ATRX | Q883R | SNP | nCFD | NSD1 | N2057D | MUT | CFD |
| ATRX | R1506L | SNP | nCFD | NSD1 | P130S | MUT | nCFD |
| ATRX | R1687T | SNP | CFD | NSD1 | P1350L | MUT | nCFD |
| ATRX | R390C | SNP | nCFD | NSD1 | P1726L | MUT | nCFD |
| ATRX | R808Q | SNP | nCFD | NSD1 | P2225S | MUT | nCFD |
| ATRX | R907Q | SNP | nCFD | NSD1 | P360L | MUT | CFD |

| | | | | | | | |
|---|---|---|---|---|---|---|---|
| ATRX | S1376N | SNP | nCFD | NSD1 | P530L | MUT | nCFD |
| ATRX | S1732C | SNP | CFD | NSD1 | P884S | MUT | nCFD |
| ATRX | S1990T | SNP | nCFD | NSD1 | R1233Q | MUT | nCFD |
| ATRX | S550G | SNP | nCFD | NSD1 | R2219H | MUT | nCFD |
| ATRX | T1146N | SNP | nCFD | NSD1 | R362Q | MUT | CFD |
| ATRX | T1146S | SNP | nCFD | NSD1 | S213N | MUT | nCFD |
| ATRX | T870I | SNP | nCFD | NSD1 | S316A | MUT | nCFD |
| ATRX | V1181L | SNP | nCFD | NSD1 | S486C | MUT | nCFD |
| ATRX | V936L | SNP | nCFD | NSD1 | S868P | MUT | nCFD |
| ATRX | Y615H | SNP | nCFD | NSD1 | S965A | MUT | nCFD |
| BCOR | A1537T | MUT | CFD | NSD1 | T2029A | MUT | CFD |
| BCOR | A967V | MUT | nCFD | NSD1 | T266A | MUT | nCFD |
| BCOR | D1536N | MUT | CFD | NSD1 | V1620I | MUT | nCFD |
| BCOR | E1005K | MUT | nCFD | NSD1 | V614L | MUT | nCFD |
| BCOR | E1544K | MUT | CFD | NSD1 | Y1971C | MUT | CFD |
| BCOR | G154E | MUT | nCFD | NSD1 | A1036P | SNP | nCFD |
| BCOR | G421V | MUT | nCFD | NSD1 | A2546T | SNP | nCFD |
| BCOR | G479R | MUT | nCFD | NSD1 | A2594T | SNP | nCFD |
| BCOR | H1367D | MUT | CFD | NSD1 | A2684T | SNP | nCFD |
| BCOR | K1228R | MUT | CFD | NSD1 | A2691T | SNP | nCFD |
| BCOR | L1203H | MUT | CFD | NSD1 | A555T | SNP | nCFD |
| BCOR | N1459S | MUT | nCFD | NSD1 | A564S | SNP | nCFD |
| BCOR | N629S | MUT | nCFD | NSD1 | A691T | SNP | nCFD |
| BCOR | P1205S | MUT | CFD | NSD1 | C310R | SNP | nCFD |
| BCOR | P663T | MUT | nCFD | NSD1 | D163N | SNP | nCFD |
| BCOR | P840S | MUT | nCFD | NSD1 | D163Y | SNP | nCFD |
| BCOR | R1375Q | MUT | CFD | NSD1 | D1737N | SNP | nCFD |
| BCOR | R1395Q | MUT | CFD | NSD1 | D2511H | SNP | nCFD |
| BCOR | R1469W | MUT | CFD | NSD1 | D2670V | SNP | nCFD |
| BCOR | R63K | MUT | nCFD | NSD1 | E1051K | SNP | nCFD |
| BCOR | S1122L | MUT | nCFD | NSD1 | E1130G | SNP | nCFD |
| BCOR | T1644I | MUT | CFD | NSD1 | E1248G | SNP | nCFD |
| BCOR | V594I | MUT | nCFD | NSD1 | E171D | SNP | nCFD |
| BCOR | A1037V | SNP | nCFD | NSD1 | E2692G | SNP | nCFD |
| BCOR | A1149V | SNP | nCFD | NSD1 | E990D | SNP | nCFD |
| BCOR | A184T | SNP | nCFD | NSD1 | F890Y | SNP | nCFD |
| BCOR | D551N | SNP | nCFD | NSD1 | G1132R | SNP | nCFD |
| BCOR | D814N | SNP | nCFD | NSD1 | G2496V | SNP | nCFD |
| BCOR | E99D | SNP | nCFD | NSD1 | H1096Y | SNP | nCFD |
| BCOR | G549S | SNP | nCFD | NSD1 | I274V | SNP | nCFD |
| BCOR | G81E | SNP | nCFD | NSD1 | I899T | SNP | nCFD |
| BCOR | H1745P | SNP | CFD | NSD1 | K144E | SNP | nCFD |
| BCOR | K1325R | SNP | CFD | NSD1 | K1631R | SNP | nCFD |
| BCOR | L808H | SNP | nCFD | NSD1 | K2629E | SNP | nCFD |
| BCOR | M1575T | SNP | nCFD | NSD1 | K954T | SNP | nCFD |
| BCOR | N390D | SNP | nCFD | NSD1 | L1392F | SNP | nCFD |
| BCOR | N784S | SNP | nCFD | NSD1 | L2277M | SNP | nCFD |
| BCOR | P288L | SNP | nCFD | NSD1 | L254F | SNP | nCFD |
| BCOR | P288Q | SNP | nCFD | NSD1 | L2617S | SNP | nCFD |
| BCOR | P483L | SNP | nCFD | NSD1 | L593F | SNP | nCFD |
| BCOR | R1131L | SNP | nCFD | NSD1 | L839R | SNP | nCFD |
| BCOR | R1131Q | SNP | nCFD | NSD1 | M1628T | SNP | nCFD |
| BCOR | R1231Q | SNP | CFD | NSD1 | M2250I | SNP | nCFD |
| BCOR | R1268G | SNP | CFD | NSD1 | M2261T | SNP | nCFD |
| BCOR | R79Q | SNP | nCFD | NSD1 | M455V | SNP | nCFD |
| BCOR | R850C | SNP | nCFD | NSD1 | M48V | SNP | nCFD |
| BCOR | S1747T | SNP | CFD | NSD1 | N272K | SNP | nCFD |
| BCOR | T870A | SNP | nCFD | NSD1 | N357S | SNP | CFD |

| | | | | | | | |
|---|---|---|---|---|---|---|---|
| BCOR | V1138A | SNP | nCFD | NSD1 | N556D | SNP | nCFD |
| BCOR | V679I | SNP | nCFD | NSD1 | N693S | SNP | nCFD |
| BCOR | Y393C | SNP | nCFD | NSD1 | P1060S | SNP | nCFD |
| BCOR | Y704C | SNP | nCFD | NSD1 | P1217S | SNP | nCFD |
| CREBBP | A110S | MUT | nCFD | NSD1 | P2282R | SNP | nCFD |
| CREBBP | A162V | MUT | nCFD | NSD1 | Q2239H | SNP | nCFD |
| CREBBP | A290T | MUT | nCFD | NSD1 | Q236H | SNP | nCFD |
| CREBBP | C1408Y | MUT | CFD | NSD1 | Q2579E | SNP | nCFD |
| CREBBP | C1421Y | MUT | CFD | NSD1 | Q2582P | SNP | nCFD |
| CREBBP | D1224G | MUT | CFD | NSD1 | Q610R | SNP | nCFD |
| CREBBP | D1435E | MUT | CFD | NSD1 | Q727H | SNP | nCFD |
| CREBBP | D1435G | MUT | CFD | NSD1 | Q727L | SNP | nCFD |
| CREBBP | D1435N | MUT | CFD | NSD1 | R1188S | SNP | nCFD |
| CREBBP | E1012K | MUT | nCFD | NSD1 | R1471G | SNP | nCFD |
| CREBBP | E1278K | MUT | nCFD | NSD1 | R2117Q | SNP | nCFD |
| CREBBP | E1963G | MUT | nCFD | NSD1 | R2391I | SNP | nCFD |
| CREBBP | F1484C | MUT | CFD | NSD1 | R2391T | SNP | nCFD |
| CREBBP | G1069S | MUT | nCFD | NSD1 | R525Q | SNP | nCFD |
| CREBBP | G1411E | MUT | CFD | NSD1 | R604L | SNP | nCFD |
| CREBBP | G1411R | MUT | CFD | NSD1 | R604Q | SNP | nCFD |
| CREBBP | G2229S | MUT | nCFD | NSD1 | R632Q | SNP | nCFD |
| CREBBP | G2275R | MUT | nCFD | NSD1 | R941H | SNP | nCFD |
| CREBBP | H1487Y | MUT | CFD | NSD1 | R941P | SNP | nCFD |
| CREBBP | I1437F | MUT | CFD | NSD1 | S1241T | SNP | nCFD |
| CREBBP | I1483S | MUT | CFD | NSD1 | S224L | SNP | nCFD |
| CREBBP | I1483T | MUT | CFD | NSD1 | S2342L | SNP | nCFD |
| CREBBP | I1493K | MUT | CFD | NSD1 | S2489G | SNP | nCFD |
| CREBBP | I2101M | MUT | CFD | NSD1 | S510G | SNP | nCFD |
| CREBBP | K1320R | MUT | nCFD | NSD1 | S552P | SNP | nCFD |
| CREBBP | L1434Q | MUT | CFD | NSD1 | S726P | SNP | nCFD |
| CREBBP | L1499P | MUT | CFD | NSD1 | S780L | SNP | nCFD |
| CREBBP | L1499Q | MUT | CFD | NSD1 | S817F | SNP | nCFD |
| CREBBP | M1625V | MUT | CFD | NSD1 | T1063A | SNP | nCFD |
| CREBBP | N1162D | MUT | CFD | NSD1 | T1098A | SNP | nCFD |
| CREBBP | N1547S | MUT | CFD | NSD1 | T1507M | SNP | nCFD |
| CREBBP | N1789K | MUT | CFD | NSD1 | T2286A | SNP | nCFD |
| CREBBP | P1277A | MUT | nCFD | NSD1 | T2382I | SNP | nCFD |
| CREBBP | P12H | MUT | nCFD | NSD1 | T2612I | SNP | nCFD |
| CREBBP | P1488L | MUT | CFD | NSD1 | T263A | SNP | nCFD |
| CREBBP | P1488Q | MUT | CFD | NSD1 | V2509M | SNP | nCFD |
| CREBBP | P1488R | MUT | CFD | NSD1 | W2663R | SNP | nCFD |
| CREBBP | P532T | MUT | nCFD | NSD1 | Y2549N | SNP | nCFD |
| CREBBP | P688A | MUT | nCFD | SETD2 | A2339V | MUT | nCFD |
| CREBBP | P858S | MUT | nCFD | SETD2 | C1631S | MUT | CFD |
| CREBBP | Q1491E | MUT | CFD | SETD2 | C1754R | MUT | nCFD |
| CREBBP | Q1491K | MUT | CFD | SETD2 | D1022E | MUT | nCFD |
| CREBBP | Q1500P | MUT | CFD | SETD2 | E1048Q | MUT | nCFD |
| CREBBP | Q1904H | MUT | nCFD | SETD2 | F1473L | MUT | nCFD |
| CREBBP | Q2298R | MUT | nCFD | SETD2 | G1231E | MUT | nCFD |
| CREBBP | Q2318E | MUT | nCFD | SETD2 | G675R | MUT | nCFD |
| CREBBP | R1169C | MUT | CFD | SETD2 | H756N | MUT | nCFD |
| CREBBP | R1347W | MUT | CFD | SETD2 | I2295M | MUT | nCFD |
| CREBBP | R1441P | MUT | CFD | SETD2 | I582M | MUT | nCFD |
| CREBBP | R1446C | MUT | CFD | SETD2 | K1906T | MUT | nCFD |
| CREBBP | R1446H | MUT | CFD | SETD2 | L886V | MUT | nCFD |
| CREBBP | R1446L | MUT | CFD | SETD2 | P1470L | MUT | nCFD |
| CREBBP | R1563S | MUT | CFD | SETD2 | P615T | MUT | nCFD |
| CREBBP | R1858H | MUT | nCFD | SETD2 | Q872E | MUT | nCFD |

| | | | | | | | |
|---|---|---|---|---|---|---|---|
| CREBBP | S1436N | MUT | CFD | SETD2 | R1523C | MUT | nCFD |
| CREBBP | S1436R | MUT | CFD | SETD2 | R1625C | MUT | CFD |
| CREBBP | S2184N | MUT | nCFD | SETD2 | R1625H | MUT | CFD |
| CREBBP | T1874I | MUT | nCFD | SETD2 | R1686P | MUT | nCFD |
| CREBBP | T902I | MUT | nCFD | SETD2 | R1740W | MUT | nCFD |
| CREBBP | V391I | MUT | CFD | SETD2 | R2490W | MUT | CFD |
| CREBBP | W1472G | MUT | CFD | SETD2 | R2510G | MUT | CFD |
| CREBBP | W1502R | MUT | CFD | SETD2 | S791L | MUT | nCFD |
| CREBBP | Y1450C | MUT | CFD | SETD2 | S882C | MUT | nCFD |
| CREBBP | Y1482C | MUT | CFD | SETD2 | V1190M | MUT | nCFD |
| CREBBP | Y1482H | MUT | CFD | SETD2 | V1576G | MUT | CFD |
| CREBBP | Y1482N | MUT | CFD | SETD2 | V658L | MUT | nCFD |
| CREBBP | Y1482S | MUT | CFD | SETD2 | Y1666C | MUT | CFD |
| CREBBP | Y1503D | MUT | CFD | SETD2 | A1124V | SNP | nCFD |
| CREBBP | Y1503F | MUT | CFD | SETD2 | A175S | SNP | nCFD |
| CREBBP | Y1503H | MUT | CFD | SETD2 | A1868D | SNP | nCFD |
| CREBBP | Y1503N | MUT | CFD | SETD2 | A197V | SNP | nCFD |
| CREBBP | Y1503S | MUT | CFD | SETD2 | A2350S | SNP | nCFD |
| CREBBP | Y1622C | MUT | CFD | SETD2 | A2350T | SNP | nCFD |
| CREBBP | A1907T | SNP | nCFD | SETD2 | A50T | SNP | nCFD |
| CREBBP | A2099G | SNP | CFD | SETD2 | A848V | SNP | nCFD |
| CREBBP | A2243V | SNP | nCFD | SETD2 | C836W | SNP | nCFD |
| CREBBP | A2265V | SNP | nCFD | SETD2 | D1057A | SNP | nCFD |
| CREBBP | A2347V | SNP | nCFD | SETD2 | D1890G | SNP | nCFD |
| CREBBP | A2419V | SNP | nCFD | SETD2 | D2204E | SNP | nCFD |
| CREBBP | A254T | SNP | nCFD | SETD2 | D699G | SNP | nCFD |
| CREBBP | A254V | SNP | nCFD | SETD2 | D868G | SNP | nCFD |
| CREBBP | A339G | SNP | nCFD | SETD2 | E2089K | SNP | nCFD |
| CREBBP | A787V | SNP | nCFD | SETD2 | E902Q | SNP | nCFD |
| CREBBP | A91S | SNP | nCFD | SETD2 | G1967A | SNP | nCFD |
| CREBBP | A91T | SNP | nCFD | SETD2 | G1967D | SNP | nCFD |
| CREBBP | A981T | SNP | nCFD | SETD2 | G412D | SNP | nCFD |
| CREBBP | G2050R | SNP | CFD | SETD2 | G412V | SNP | nCFD |
| CREBBP | G2126C | SNP | nCFD | SETD2 | G675E | SNP | nCFD |
| CREBBP | G2126R | SNP | nCFD | SETD2 | G933V | SNP | nCFD |
| CREBBP | G2126S | SNP | nCFD | SETD2 | I1153T | SNP | nCFD |
| CREBBP | G98V | SNP | nCFD | SETD2 | I1194V | SNP | nCFD |
| CREBBP | I1643V | SNP | CFD | SETD2 | I1398T | SNP | nCFD |
| CREBBP | I445L | SNP | nCFD | SETD2 | I1514L | SNP | nCFD |
| CREBBP | I968S | SNP | nCFD | SETD2 | I563T | SNP | nCFD |
| CREBBP | I968T | SNP | nCFD | SETD2 | I819V | SNP | nCFD |
| CREBBP | L551I | SNP | nCFD | SETD2 | K303E | SNP | nCFD |
| CREBBP | L833F | SNP | nCFD | SETD2 | K304T | SNP | nCFD |
| CREBBP | L843V | SNP | nCFD | SETD2 | K629E | SNP | nCFD |
| CREBBP | L853R | SNP | nCFD | SETD2 | K759E | SNP | nCFD |
| CREBBP | L853V | SNP | nCFD | SETD2 | L222I | SNP | nCFD |
| CREBBP | M1916T | SNP | nCFD | SETD2 | L514I | SNP | nCFD |
| CREBBP | M2161I | SNP | nCFD | SETD2 | L89F | SNP | nCFD |
| CREBBP | M2221L | SNP | nCFD | SETD2 | L981S | SNP | nCFD |
| CREBBP | M747V | SNP | nCFD | SETD2 | M1009T | SNP | nCFD |
| CREBBP | N1310S | SNP | nCFD | SETD2 | M1889T | SNP | nCFD |
| CREBBP | N1978S | SNP | nCFD | SETD2 | M45V | SNP | nCFD |
| CREBBP | N2111S | SNP | CFD | SETD2 | N2058S | SNP | nCFD |
| CREBBP | N984Y | SNP | nCFD | SETD2 | N2373S | SNP | nCFD |
| CREBBP | P1053L | SNP | nCFD | SETD2 | N579I | SNP | nCFD |
| CREBBP | P1053S | SNP | nCFD | SETD2 | N719D | SNP | nCFD |
| CREBBP | P153L | SNP | nCFD | SETD2 | N840S | SNP | nCFD |
| CREBBP | P1608T | SNP | CFD | SETD2 | N951D | SNP | nCFD |

| | | | | | | | |
|---|---|---|---|---|---|---|---|
| CREBBP | P1947S | SNP | nCFD | SETD2 | P1028S | SNP | nCFD |
| CREBBP | P578S | SNP | nCFD | SETD2 | P104T | SNP | nCFD |
| CREBBP | P847T | SNP | nCFD | SETD2 | P1141L | SNP | nCFD |
| CREBBP | Q136E | SNP | nCFD | SETD2 | P167L | SNP | nCFD |
| CREBBP | Q2200L | SNP | nCFD | SETD2 | P186L | SNP | nCFD |
| CREBBP | Q2208H | SNP | nCFD | SETD2 | P190L | SNP | nCFD |
| CREBBP | Q2215E | SNP | nCFD | SETD2 | P193L | SNP | nCFD |
| CREBBP | Q278P | SNP | nCFD | SETD2 | P2057S | SNP | nCFD |
| CREBBP | R1682C | SNP | nCFD | SETD2 | P2379L | SNP | nCFD |
| CREBBP | R386Q | SNP | CFD | SETD2 | P617S | SNP | nCFD |
| CREBBP | S1043L | SNP | nCFD | SETD2 | P751T | SNP | nCFD |
| CREBBP | S128C | SNP | nCFD | SETD2 | Q1147R | SNP | nCFD |
| CREBBP | S1386C | SNP | CFD | SETD2 | Q1734H | SNP | nCFD |
| CREBBP | S141C | SNP | nCFD | SETD2 | Q1981R | SNP | nCFD |
| CREBBP | S2425C | SNP | nCFD | SETD2 | Q662H | SNP | nCFD |
| CREBBP | S299G | SNP | nCFD | SETD2 | Q68R | SNP | nCFD |
| CREBBP | S56C | SNP | nCFD | SETD2 | R1297H | SNP | nCFD |
| CREBBP | S56R | SNP | nCFD | SETD2 | R1335H | SNP | nCFD |
| CREBBP | S861F | SNP | nCFD | SETD2 | R1509K | SNP | nCFD |
| CREBBP | S893L | SNP | nCFD | SETD2 | R329W | SNP | nCFD |
| CREBBP | S893W | SNP | nCFD | SETD2 | R402Q | SNP | nCFD |
| CREBBP | T1468I | SNP | CFD | SETD2 | R414G | SNP | nCFD |
| CREBBP | T1575A | SNP | CFD | SETD2 | R433C | SNP | nCFD |
| CREBBP | T910A | SNP | nCFD | SETD2 | R448Q | SNP | nCFD |
| CREBBP | T910S | SNP | nCFD | SETD2 | R471K | SNP | nCFD |
| CREBBP | T941N | SNP | nCFD | SETD2 | R472H | SNP | nCFD |
| CREBBP | V701L | SNP | nCFD | SETD2 | R541Q | SNP | nCFD |
| CREBBP | V992I | SNP | nCFD | SETD2 | R976G | SNP | nCFD |
| CREBBP | V994M | SNP | nCFD | SETD2 | S1001C | SNP | nCFD |
| CREBBP | W1151C | SNP | CFD | SETD2 | S1059N | SNP | nCFD |
| CREBBP | Y1204F | SNP | CFD | SETD2 | S130Y | SNP | nCFD |
| DNMT3A | A368T | MUT | CFD | SETD2 | S1888I | SNP | nCFD |
| DNMT3A | A410T | MUT | nCFD | SETD2 | S1940G | SNP | nCFD |
| DNMT3A | A741V | MUT | CFD | SETD2 | S1940N | SNP | nCFD |
| DNMT3A | A910P | MUT | nCFD | SETD2 | S2193N | SNP | nCFD |
| DNMT3A | A910V | MUT | nCFD | SETD2 | S2279A | SNP | nCFD |
| DNMT3A | C562Y | MUT | nCFD | SETD2 | S2424N | SNP | nCFD |
| DNMT3A | D11A | MUT | nCFD | SETD2 | S262R | SNP | nCFD |
| DNMT3A | D389N | MUT | nCFD | SETD2 | S705L | SNP | nCFD |
| DNMT3A | D614Y | MUT | nCFD | SETD2 | S708F | SNP | nCFD |
| DNMT3A | D748N | MUT | CFD | SETD2 | T1033A | SNP | nCFD |
| DNMT3A | D857N | MUT | nCFD | SETD2 | T1077A | SNP | nCFD |
| DNMT3A | E119V | MUT | nCFD | SETD2 | T1483A | SNP | nCFD |
| DNMT3A | E733G | MUT | CFD | SETD2 | T1483S | SNP | nCFD |
| DNMT3A | F414L | MUT | nCFD | SETD2 | T1866A | SNP | nCFD |
| DNMT3A | F731C | MUT | CFD | SETD2 | T2037A | SNP | nCFD |
| DNMT3A | F731V | MUT | CFD | SETD2 | T592K | SNP | nCFD |
| DNMT3A | F732L | MUT | CFD | SETD2 | T767A | SNP | nCFD |
| DNMT3A | F732S | MUT | CFD | SETD2 | T773A | SNP | nCFD |
| DNMT3A | F752L | MUT | CFD | SETD2 | T928R | SNP | nCFD |
| DNMT3A | F868S | MUT | nCFD | SETD2 | V1938I | SNP | nCFD |
| DNMT3A | F870L | MUT | nCFD | SETD2 | V2229G | SNP | nCFD |
| DNMT3A | F909C | MUT | nCFD | SETD2 | V2259L | SNP | nCFD |
| DNMT3A | G308D | MUT | CFD | SETD2 | V267I | SNP | nCFD |
| DNMT3A | G332R | MUT | CFD | SETD2 | V768L | SNP | nCFD |
| DNMT3A | G543C | MUT | nCFD | SETD2 | V816D | SNP | nCFD |
| DNMT3A | G646E | MUT | CFD | SETD2 | Y1094C | SNP | nCFD |
| DNMT3A | G685R | MUT | CFD | SETD2 | Y419C | SNP | nCFD |

| | | | | | | | |
|---|---|---|---|---|---|---|---|
| DNMT3A | G699D | MUT | CFD | SETD2 | Y555S | SNP | nCFD |
| DNMT3A | G699R | MUT | CFD | SF3B1 | A1229V | MUT | nCFD |
| DNMT3A | G699S | MUT | CFD | SF3B1 | A364V | MUT | CFD |
| DNMT3A | G707D | MUT | CFD | SF3B1 | A672T | MUT | nCFD |
| DNMT3A | G728D | MUT | CFD | SF3B1 | A708T | MUT | nCFD |
| DNMT3A | G762C | MUT | CFD | SF3B1 | A744P | MUT | nCFD |
| DNMT3A | H873N | MUT | nCFD | SF3B1 | C1204R | MUT | nCFD |
| DNMT3A | I369N | MUT | CFD | SF3B1 | D781G | MUT | nCFD |
| DNMT3A | I655N | MUT | CFD | SF3B1 | D894G | MUT | nCFD |
| DNMT3A | I695T | MUT | CFD | SF3B1 | D894N | MUT | nCFD |
| DNMT3A | I705T | MUT | CFD | SF3B1 | E622D | MUT | nCFD |
| DNMT3A | I780T | MUT | nCFD | SF3B1 | E862K | MUT | nCFD |
| DNMT3A | K468R | MUT | nCFD | SF3B1 | G1146R | MUT | nCFD |
| DNMT3A | K54N | MUT | nCFD | SF3B1 | G605S | MUT | nCFD |
| DNMT3A | K826N | MUT | nCFD | SF3B1 | G740E | MUT | nCFD |
| DNMT3A | K829R | MUT | nCFD | SF3B1 | G740R | MUT | nCFD |
| DNMT3A | K841Q | MUT | nCFD | SF3B1 | G742D | MUT | nCFD |
| DNMT3A | L344Q | MUT | CFD | SF3B1 | G751V | MUT | nCFD |
| DNMT3A | L547R | MUT | nCFD | SF3B1 | H662D | MUT | nCFD |
| DNMT3A | L653W | MUT | CFD | SF3B1 | H662Q | MUT | nCFD |
| DNMT3A | L703V | MUT | CFD | SF3B1 | H738Y | MUT | nCFD |
| DNMT3A | L737R | MUT | CFD | SF3B1 | I704F | MUT | nCFD |
| DNMT3A | L754R | MUT | CFD | SF3B1 | I704N | MUT | nCFD |
| DNMT3A | L805F | MUT | nCFD | SF3B1 | I704S | MUT | nCFD |
| DNMT3A | L889P | MUT | nCFD | SF3B1 | I704V | MUT | nCFD |
| DNMT3A | M880I | MUT | nCFD | SF3B1 | K666E | MUT | nCFD |
| DNMT3A | M880V | MUT | nCFD | SF3B1 | K666M | MUT | nCFD |
| DNMT3A | N838D | MUT | nCFD | SF3B1 | K666N | MUT | nCFD |
| DNMT3A | N879S | MUT | nCFD | SF3B1 | K666Q | MUT | nCFD |
| DNMT3A | P718L | MUT | CFD | SF3B1 | K666R | MUT | nCFD |
| DNMT3A | P799A | MUT | nCFD | SF3B1 | K666T | MUT | nCFD |
| DNMT3A | P849L | MUT | nCFD | SF3B1 | K700E | MUT | nCFD |
| DNMT3A | P849R | MUT | nCFD | SF3B1 | K741E | MUT | nCFD |
| DNMT3A | P904L | MUT | nCFD | SF3B1 | K741N | MUT | nCFD |
| DNMT3A | Q356R | MUT | CFD | SF3B1 | N619K | MUT | nCFD |
| DNMT3A | Q842E | MUT | nCFD | SF3B1 | N626D | MUT | nCFD |
| DNMT3A | Q886R | MUT | nCFD | SF3B1 | N626H | MUT | nCFD |
| DNMT3A | R326C | MUT | CFD | SF3B1 | N626Y | MUT | nCFD |
| DNMT3A | R366P | MUT | CFD | SF3B1 | P1224T | MUT | nCFD |
| DNMT3A | R474S | MUT | nCFD | SF3B1 | Q659R | MUT | nCFD |
| DNMT3A | R635Q | MUT | CFD | SF3B1 | Q670E | MUT | nCFD |
| DNMT3A | R635W | MUT | CFD | SF3B1 | Q903R | MUT | nCFD |
| DNMT3A | R659C | MUT | CFD | SF3B1 | R625C | MUT | nCFD |
| DNMT3A | R676W | MUT | CFD | SF3B1 | R625G | MUT | nCFD |
| DNMT3A | R688H | MUT | CFD | SF3B1 | R625L | MUT | nCFD |
| DNMT3A | R720C | MUT | CFD | SF3B1 | R625S | MUT | nCFD |
| DNMT3A | R720G | MUT | CFD | SF3B1 | R630S | MUT | nCFD |
| DNMT3A | R720H | MUT | CFD | SF3B1 | T663I | MUT | nCFD |
| DNMT3A | R729Q | MUT | CFD | SF3B1 | V701F | MUT | nCFD |
| DNMT3A | R729W | MUT | CFD | SF3B1 | Y623C | MUT | nCFD |
| DNMT3A | R736C | MUT | CFD | SF3B1 | H8Y | SNP | nCFD |
| DNMT3A | R736H | MUT | CFD | SF3B1 | K700Q | SNP | nCFD |
| DNMT3A | R749C | MUT | CFD | SF3B1 | T1096K | SNP | nCFD |
| DNMT3A | R771L | MUT | CFD | SF3B1 | T7I | SNP | nCFD |
| DNMT3A | R792H | MUT | nCFD | SF3B1 | V961I | SNP | nCFD |
| DNMT3A | R803S | MUT | nCFD | SPEN | A1745E | MUT | nCFD |
| DNMT3A | R882C | MUT | nCFD | SPEN | A2037S | MUT | nCFD |
| DNMT3A | R882H | MUT | nCFD | SPEN | A2721T | MUT | nCFD |

| | | | | | | | |
|---|---|---|---|---|---|---|---|
| DNMT3A | R882L | MUT | nCFD | SPEN | A970V | MUT | nCFD |
| DNMT3A | R882P | MUT | nCFD | SPEN | C2567S | MUT | nCFD |
| DNMT3A | R882S | MUT | nCFD | SPEN | D605Y | MUT | nCFD |
| DNMT3A | S352N | MUT | CFD | SPEN | G2935W | MUT | nCFD |
| DNMT3A | S535P | MUT | nCFD | SPEN | G3562R | MUT | CFD |
| DNMT3A | S714C | MUT | CFD | SPEN | H3638P | MUT | CFD |
| DNMT3A | S770L | MUT | CFD | SPEN | K1064E | MUT | nCFD |
| DNMT3A | S770W | MUT | CFD | SPEN | L1091P | MUT | nCFD |
| DNMT3A | S828N | MUT | nCFD | SPEN | L2990V | MUT | nCFD |
| DNMT3A | S839Y | MUT | nCFD | SPEN | L440P | MUT | CFD |
| DNMT3A | V265L | MUT | nCFD | SPEN | N1748T | MUT | nCFD |
| DNMT3A | V296L | MUT | CFD | SPEN | N2360D | MUT | nCFD |
| DNMT3A | V339M | MUT | CFD | SPEN | P1810A | MUT | nCFD |
| DNMT3A | V636M | MUT | CFD | SPEN | P3345S | MUT | nCFD |
| DNMT3A | V665G | MUT | CFD | SPEN | R187Q | MUT | nCFD |
| DNMT3A | V704M | MUT | CFD | SPEN | R1902W | MUT | nCFD |
| DNMT3A | V716I | MUT | CFD | SPEN | R1949W | MUT | nCFD |
| DNMT3A | V897D | MUT | nCFD | SPEN | R423C | MUT | nCFD |
| DNMT3A | W409R | MUT | nCFD | SPEN | S2221F | MUT | nCFD |
| DNMT3A | W581C | MUT | nCFD | SPEN | S260N | MUT | nCFD |
| DNMT3A | W753G | MUT | CFD | SPEN | A1486T | SNP | nCFD |
| DNMT3A | W795C | MUT | nCFD | SPEN | A1665V | SNP | nCFD |
| DNMT3A | W860R | MUT | nCFD | SPEN | A1713S | SNP | nCFD |
| DNMT3A | Y660F | MUT | CFD | SPEN | A2058P | SNP | nCFD |
| DNMT3A | Y735C | MUT | CFD | SPEN | A2058T | SNP | nCFD |
| DNMT3A | Y735S | MUT | CFD | SPEN | A2058V | SNP | nCFD |
| DNMT3A | E102D | SNP | nCFD | SPEN | A2199S | SNP | nCFD |
| DNMT3A | E30A | SNP | nCFD | SPEN | A2653T | SNP | nCFD |
| DNMT3A | F755C | SNP | CFD | SPEN | A2729V | SNP | nCFD |
| DNMT3A | F755S | SNP | CFD | SPEN | A2745V | SNP | nCFD |
| DNMT3A | G172A | SNP | nCFD | SPEN | A2777V | SNP | nCFD |
| DNMT3A | M161L | SNP | nCFD | SPEN | A2800V | SNP | nCFD |
| DNMT3A | M161V | SNP | nCFD | SPEN | A3167D | SNP | nCFD |
| DNMT3A | N501S | SNP | nCFD | SPEN | D1234N | SNP | nCFD |
| DNMT3A | P904Q | SNP | nCFD | SPEN | D1363E | SNP | nCFD |
| DNMT3A | P904R | SNP | nCFD | SPEN | D2007E | SNP | nCFD |
| DNMT3A | S663L | SNP | CFD | SPEN | D2606E | SNP | nCFD |
| DNMT3A | S775P | SNP | CFD | SPEN | D303G | SNP | nCFD |
| DNMT3A | V480G | SNP | nCFD | SPEN | D303V | SNP | nCFD |
| DNMT3A | V483G | SNP | nCFD | SPEN | E1271A | SNP | nCFD |
| EP300 | A1189V | MUT | CFD | SPEN | E1768K | SNP | nCFD |
| EP300 | A13V | MUT | nCFD | SPEN | E1792K | SNP | nCFD |
| EP300 | A1498T | MUT | CFD | SPEN | E2379K | SNP | nCFD |
| EP300 | A2089G | MUT | CFD | SPEN | E923K | SNP | nCFD |
| EP300 | A2259V | MUT | nCFD | SPEN | G158V | SNP | nCFD |
| EP300 | A2289V | MUT | nCFD | SPEN | G2157V | SNP | nCFD |
| EP300 | A2354V | MUT | nCFD | SPEN | G2294R | SNP | nCFD |
| EP300 | A921T | MUT | nCFD | SPEN | G2294S | SNP | nCFD |
| EP300 | C1164Y | MUT | CFD | SPEN | G3464A | SNP | nCFD |
| EP300 | C1201Y | MUT | nCFD | SPEN | G553S | SNP | CFD |
| EP300 | C1438R | MUT | CFD | SPEN | H1914Y | SNP | nCFD |
| EP300 | C1683Y | MUT | CFD | SPEN | I2469V | SNP | nCFD |
| EP300 | C1790G | MUT | CFD | SPEN | K1609R | SNP | nCFD |
| EP300 | C369F | MUT | CFD | SPEN | K1820R | SNP | nCFD |
| EP300 | C819Y | MUT | nCFD | SPEN | K2971R | SNP | nCFD |
| EP300 | D1625V | MUT | nCFD | SPEN | K909T | SNP | nCFD |
| EP300 | E1263V | MUT | nCFD | SPEN | K943E | SNP | nCFD |
| EP300 | E1536K | MUT | CFD | SPEN | L1091V | SNP | nCFD |

| | | | | | | | |
|---|---|---|---|---|---|---|---|
| EP300 | G1368S | MUT | CFD | SPEN | L1136P | SNP | nCFD |
| EP300 | G1382D | MUT | CFD | SPEN | L3512P | SNP | CFD |
| EP300 | G1572R | MUT | CFD | SPEN | L3512Q | SNP | CFD |
| EP300 | G787S | MUT | nCFD | SPEN | L3512R | SNP | CFD |
| EP300 | H1449Q | MUT | CFD | SPEN | L3512V | SNP | CFD |
| EP300 | I1086V | MUT | CFD | SPEN | L808V | SNP | nCFD |
| EP300 | I947T | MUT | nCFD | SPEN | M2169L | SNP | nCFD |
| EP300 | L1360P | MUT | CFD | SPEN | N2593S | SNP | nCFD |
| EP300 | L1639P | MUT | nCFD | SPEN | P1007T | SNP | nCFD |
| EP300 | L2376I | MUT | nCFD | SPEN | P1296A | SNP | nCFD |
| EP300 | L415P | MUT | nCFD | SPEN | P1691S | SNP | nCFD |
| EP300 | L633P | MUT | CFD | SPEN | P1795S | SNP | nCFD |
| EP300 | M104I | MUT | nCFD | SPEN | P2004L | SNP | nCFD |
| EP300 | M2175L | MUT | nCFD | SPEN | P2054T | SNP | nCFD |
| EP300 | M2372V | MUT | nCFD | SPEN | P2067L | SNP | nCFD |
| EP300 | M514I | MUT | nCFD | SPEN | P2158L | SNP | nCFD |
| EP300 | N581H | MUT | CFD | SPEN | P2237A | SNP | nCFD |
| EP300 | P1439Q | MUT | CFD | SPEN | P2238S | SNP | nCFD |
| EP300 | P1879S | MUT | nCFD | SPEN | P2240L | SNP | nCFD |
| EP300 | P250S | MUT | nCFD | SPEN | P2240R | SNP | nCFD |
| EP300 | P300L | MUT | nCFD | SPEN | P2433L | SNP | nCFD |
| EP300 | P481L | MUT | nCFD | SPEN | P2433R | SNP | nCFD |
| EP300 | P481Q | MUT | nCFD | SPEN | P2574L | SNP | nCFD |
| EP300 | P766L | MUT | nCFD | SPEN | P2710S | SNP | nCFD |
| EP300 | P882R | MUT | nCFD | SPEN | P2984S | SNP | nCFD |
| EP300 | P925L | MUT | nCFD | SPEN | P3002L | SNP | nCFD |
| EP300 | P925T | MUT | nCFD | SPEN | P3123S | SNP | nCFD |
| EP300 | Q2195R | MUT | nCFD | SPEN | P3165A | SNP | nCFD |
| EP300 | Q341P | MUT | CFD | SPEN | P3257S | SNP | nCFD |
| EP300 | R1410W | MUT | CFD | SPEN | P3259S | SNP | nCFD |
| EP300 | R1627W | MUT | nCFD | SPEN | P3346S | SNP | nCFD |
| EP300 | R838S | MUT | nCFD | SPEN | P3346T | SNP | nCFD |
| EP300 | S1136P | MUT | CFD | SPEN | P542S | SNP | CFD |
| EP300 | S1212N | MUT | nCFD | SPEN | P771S | SNP | nCFD |
| EP300 | S1220C | MUT | nCFD | SPEN | P876A | SNP | nCFD |
| EP300 | S1534G | MUT | CFD | SPEN | Q2020R | SNP | nCFD |
| EP300 | S255L | MUT | nCFD | SPEN | Q261K | SNP | nCFD |
| EP300 | T132S | MUT | nCFD | SPEN | Q3324R | SNP | nCFD |
| EP300 | T1491S | MUT | CFD | SPEN | Q3493E | SNP | nCFD |
| EP300 | T1669I | MUT | CFD | SPEN | Q822E | SNP | nCFD |
| EP300 | T558A | MUT | nCFD | SPEN | Q918E | SNP | nCFD |
| EP300 | T594M | MUT | CFD | SPEN | R1143C | SNP | nCFD |
| EP300 | T890P | MUT | nCFD | SPEN | R1303S | SNP | nCFD |
| EP300 | V361M | MUT | CFD | SPEN | R1475Q | SNP | nCFD |
| EP300 | Y1414C | MUT | CFD | SPEN | R1959G | SNP | nCFD |
| EP300 | Y1467D | MUT | CFD | SPEN | R1985S | SNP | nCFD |
| EP300 | Y1467H | MUT | CFD | SPEN | R2010H | SNP | nCFD |
| EP300 | Y1467N | MUT | CFD | SPEN | R2043C | SNP | nCFD |
| EP300 | Y1483C | MUT | CFD | SPEN | R235W | SNP | nCFD |
| EP300 | Y638C | MUT | CFD | SPEN | R267G | SNP | nCFD |
| EP300 | A171V | SNP | nCFD | SPEN | R3136C | SNP | nCFD |
| EP300 | A2014V | SNP | CFD | SPEN | R3168G | SNP | nCFD |
| EP300 | A2028V | SNP | CFD | SPEN | R3185Q | SNP | nCFD |
| EP300 | A2165V | SNP | nCFD | SPEN | R3312C | SNP | nCFD |
| EP300 | A357P | SNP | CFD | SPEN | R3536W | SNP | CFD |
| EP300 | A922V | SNP | nCFD | SPEN | R637Q | SNP | nCFD |
| EP300 | C1779G | SNP | CFD | SPEN | R75C | SNP | CFD |
| EP300 | E3K | SNP | nCFD | SPEN | R784H | SNP | nCFD |

| | | | | | | | |
|---|---|---|---|---|---|---|---|
| EP300 | G2032R | SNP | CFD | SPEN | R807H | SNP | nCFD |
| EP300 | G211S | SNP | nCFD | SPEN | R807P | SNP | nCFD |
| EP300 | G271V | SNP | nCFD | SPEN | R824C | SNP | nCFD |
| EP300 | G324D | SNP | nCFD | SPEN | R871C | SNP | nCFD |
| EP300 | G663V | SNP | nCFD | SPEN | S101G | SNP | nCFD |
| EP300 | H2339N | SNP | nCFD | SPEN | S1189P | SNP | nCFD |
| EP300 | H2339Y | SNP | nCFD | SPEN | S2139T | SNP | nCFD |
| EP300 | H2414Y | SNP | nCFD | SPEN | S2426F | SNP | nCFD |
| EP300 | I1693V | SNP | CFD | SPEN | S2452F | SNP | nCFD |
| EP300 | I196V | SNP | nCFD | SPEN | S2493R | SNP | nCFD |
| EP300 | I429V | SNP | nCFD | SPEN | S2525F | SNP | nCFD |
| EP300 | I725V | SNP | nCFD | SPEN | S2553G | SNP | nCFD |
| EP300 | I859T | SNP | nCFD | SPEN | S260R | SNP | nCFD |
| EP300 | I997V | SNP | nCFD | SPEN | S2841G | SNP | nCFD |
| EP300 | L2406P | SNP | nCFD | SPEN | S3207C | SNP | nCFD |
| EP300 | M126V | SNP | nCFD | SPEN | S3223R | SNP | nCFD |
| EP300 | M2130I | SNP | nCFD | SPEN | S3466G | SNP | nCFD |
| EP300 | M2133T | SNP | nCFD | SPEN | S3492C | SNP | nCFD |
| EP300 | M2161V | SNP | nCFD | SPEN | T1633I | SNP | nCFD |
| EP300 | M2168I | SNP | nCFD | SPEN | T1646S | SNP | nCFD |
| EP300 | M2382T | SNP | nCFD | SPEN | T1662M | SNP | nCFD |
| EP300 | M247V | SNP | nCFD | SPEN | T2983M | SNP | nCFD |
| EP300 | M289V | SNP | nCFD | SPEN | T3204M | SNP | nCFD |
| EP300 | M664T | SNP | nCFD | SPEN | T3558M | SNP | CFD |
| EP300 | M664V | SNP | nCFD | SPEN | T922M | SNP | nCFD |
| EP300 | M693T | SNP | nCFD | SPEN | V1014M | SNP | nCFD |
| EP300 | N182S | SNP | nCFD | SPEN | V1022M | SNP | nCFD |
| EP300 | N2379H | SNP | nCFD | SPEN | V1637A | SNP | nCFD |
| EP300 | N249S | SNP | nCFD | SPEN | V1645L | SNP | nCFD |
| EP300 | N493S | SNP | nCFD | SPEN | V1920I | SNP | nCFD |
| EP300 | P1222H | SNP | nCFD | SPEN | V1924I | SNP | nCFD |
| EP300 | P1855L | SNP | nCFD | SPEN | V2023M | SNP | nCFD |
| EP300 | P1875L | SNP | nCFD | SPEN | V2690M | SNP | nCFD |
| EP300 | P1875S | SNP | nCFD | SPEN | V2704M | SNP | nCFD |
| EP300 | P1875T | SNP | nCFD | SPEN | V2741I | SNP | nCFD |
| EP300 | P1986L | SNP | nCFD | SPEN | V3250A | SNP | nCFD |
| EP300 | P1986Q | SNP | nCFD | SPEN | V3472A | SNP | nCFD |
| EP300 | P1986R | SNP | nCFD | SPEN | V3496A | SNP | nCFD |
| EP300 | P2031S | SNP | CFD | SPEN | V3496M | SNP | nCFD |
| EP300 | P2043Q | SNP | CFD | SPEN | V3527I | SNP | CFD |
| EP300 | P2115L | SNP | nCFD | SPEN | V3569G | SNP | CFD |
| EP300 | P2220R | SNP | nCFD | SPEN | V828A | SNP | nCFD |
| EP300 | P2312S | SNP | nCFD | SPEN | V828I | SNP | nCFD |
| EP300 | P747L | SNP | nCFD | SPEN | Y1297C | SNP | nCFD |
| EP300 | P784L | SNP | nCFD | SPEN | Y3624H | SNP | CFD |
| EP300 | P834T | SNP | nCFD | TET2 | A1153E | MUT | nCFD |
| EP300 | P870L | SNP | nCFD | TET2 | A1241D | MUT | nCFD |
| EP300 | P886S | SNP | nCFD | TET2 | A1355V | MUT | CFD |
| EP300 | P934L | SNP | nCFD | TET2 | A1379V | MUT | CFD |
| EP300 | Q1836H | SNP | nCFD | TET2 | A1505T | MUT | CFD |
| EP300 | Q190R | SNP | nCFD | TET2 | A1505V | MUT | CFD |
| EP300 | Q1990R | SNP | nCFD | TET2 | A1863D | MUT | CFD |
| EP300 | Q2223P | SNP | nCFD | TET2 | A1863T | MUT | CFD |
| EP300 | Q2241H | SNP | nCFD | TET2 | A1876V | MUT | CFD |
| EP300 | Q229H | SNP | nCFD | TET2 | A1882P | MUT | CFD |
| EP300 | R1665C | SNP | CFD | TET2 | A827T | MUT | nCFD |
| EP300 | R2088W | SNP | CFD | TET2 | C1135Y | MUT | nCFD |
| EP300 | R695C | SNP | nCFD | TET2 | C1193W | MUT | nCFD |

| | | | | | | | |
|---|---|---|---|---|---|---|---|
| EP300 | R705Q | SNP | nCFD | TET2 | C1211R | MUT | nCFD |
| EP300 | S106G | SNP | nCFD | TET2 | C1211Y | MUT | nCFD |
| EP300 | S1716T | SNP | nCFD | TET2 | C1221Y | MUT | nCFD |
| EP300 | S2328P | SNP | nCFD | TET2 | C1263R | MUT | nCFD |
| EP300 | S35A | SNP | nCFD | TET2 | C1263Y | MUT | nCFD |
| EP300 | S507G | SNP | nCFD | TET2 | C1271G | MUT | nCFD |
| EP300 | S545I | SNP | nCFD | TET2 | C1271S | MUT | nCFD |
| EP300 | S545N | SNP | nCFD | TET2 | C1271W | MUT | nCFD |
| EP300 | S545T | SNP | nCFD | TET2 | C1271Y | MUT | nCFD |
| EP300 | S697R | SNP | nCFD | TET2 | C1289F | MUT | nCFD |
| EP300 | S719G | SNP | nCFD | TET2 | C1289Y | MUT | nCFD |
| EP300 | S916T | SNP | nCFD | TET2 | C1298G | MUT | CFD |
| EP300 | S952P | SNP | nCFD | TET2 | C1298T | MUT | CFD |
| EP300 | T1282A | SNP | nCFD | TET2 | C1298Y | MUT | CFD |
| EP300 | T1851P | SNP | nCFD | TET2 | C1358G | MUT | CFD |
| EP300 | T1909A | SNP | nCFD | TET2 | C1358R | MUT | CFD |
| EP300 | T1909P | SNP | nCFD | TET2 | C1378R | MUT | CFD |
| EP300 | T258A | SNP | nCFD | TET2 | C1378Y | MUT | CFD |
| EP300 | T576N | SNP | CFD | TET2 | C1875R | MUT | CFD |
| EP300 | T858A | SNP | nCFD | TET2 | D1242V | MUT | nCFD |
| EP300 | V520I | SNP | nCFD | TET2 | D1376E | MUT | CFD |
| EP300 | Y207H | SNP | nCFD | TET2 | D1376N | MUT | CFD |
| EZH2 | A576V | MUT | nCFD | TET2 | D1427Y | MUT | CFD |
| EZH2 | A651V | MUT | CFD | TET2 | D1730G | MUT | CFD |
| EZH2 | A677G | MUT | CFD | TET2 | E1144A | MUT | nCFD |
| EZH2 | A687V | MUT | CFD | TET2 | E1207Q | MUT | nCFD |
| EZH2 | C523R | MUT | nCFD | TET2 | E1222G | MUT | nCFD |
| EZH2 | C549R | MUT | nCFD | TET2 | E1234G | MUT | nCFD |
| EZH2 | C585W | MUT | nCFD | TET2 | E1318D | MUT | CFD |
| EZH2 | C601Y | MUT | nCFD | TET2 | E1492G | MUT | CFD |
| EZH2 | D188N | MUT | nCFD | TET2 | E1492K | MUT | CFD |
| EZH2 | D659A | MUT | CFD | TET2 | E1492V | MUT | CFD |
| EZH2 | D659G | MUT | CFD | TET2 | E1513Q | MUT | CFD |
| EZH2 | D672H | MUT | CFD | TET2 | E1874G | MUT | CFD |
| EZH2 | E125V | MUT | nCFD | TET2 | E788K | MUT | nCFD |
| EZH2 | E249K | MUT | nCFD | TET2 | E971K | MUT | nCFD |
| EZH2 | F145L | MUT | nCFD | TET2 | F1287S | MUT | nCFD |
| EZH2 | F145S | MUT | nCFD | TET2 | F1300C | MUT | CFD |
| EZH2 | F665L | MUT | CFD | TET2 | F1300I | MUT | CFD |
| EZH2 | F665S | MUT | CFD | TET2 | F1368L | MUT | CFD |
| EZH2 | G159R | MUT | nCFD | TET2 | F1368Y | MUT | CFD |
| EZH2 | G330R | MUT | nCFD | TET2 | F1377I | MUT | CFD |
| EZH2 | G5R | MUT | nCFD | TET2 | F1377L | MUT | CFD |
| EZH2 | G643E | MUT | CFD | TET2 | F1377S | MUT | CFD |
| EZH2 | G655R | MUT | CFD | TET2 | G1172S | MUT | nCFD |
| EZH2 | H279Q | MUT | nCFD | TET2 | G1192R | MUT | nCFD |
| EZH2 | H525N | MUT | nCFD | TET2 | G1235R | MUT | nCFD |
| EZH2 | H689R | MUT | CFD | TET2 | G1256A | MUT | nCFD |
| EZH2 | I146N | MUT | nCFD | TET2 | G1256D | MUT | nCFD |
| EZH2 | I264T | MUT | nCFD | TET2 | G1275E | MUT | nCFD |
| EZH2 | K199N | MUT | nCFD | TET2 | G1275R | MUT | nCFD |
| EZH2 | K629E | MUT | CFD | TET2 | G1282D | MUT | nCFD |
| EZH2 | K656E | MUT | CFD | TET2 | G1282R | MUT | nCFD |
| EZH2 | L149Q | MUT | nCFD | TET2 | G1288D | MUT | nCFD |
| EZH2 | L26P | MUT | nCFD | TET2 | G1288S | MUT | nCFD |
| EZH2 | L666V | MUT | CFD | TET2 | G1288V | MUT | nCFD |
| EZH2 | L669S | MUT | CFD | TET2 | G1361D | MUT | CFD |
| EZH2 | L669V | MUT | CFD | TET2 | G1365V | MUT | CFD |

| | | | | | | | |
|---|---|---|---|---|---|---|---|
| EZH2 | N670K | MUT | CFD | TET2 | G1370R | MUT | CFD |
| EZH2 | N688K | MUT | CFD | TET2 | G1370V | MUT | CFD |
| EZH2 | N688T | MUT | CFD | TET2 | G1370W | MUT | CFD |
| EZH2 | R288Q | MUT | nCFD | TET2 | G1735R | MUT | CFD |
| EZH2 | R382W | MUT | nCFD | TET2 | G1861E | MUT | CFD |
| EZH2 | R497Q | MUT | nCFD | TET2 | G1913D | MUT | nCFD |
| EZH2 | R556P | MUT | nCFD | TET2 | G210D | MUT | nCFD |
| EZH2 | R561L | MUT | nCFD | TET2 | G494R | MUT | nCFD |
| EZH2 | R654K | MUT | CFD | TET2 | G520S | MUT | nCFD |
| EZH2 | R679C | MUT | CFD | TET2 | G773V | MUT | nCFD |
| EZH2 | R679H | MUT | CFD | TET2 | H1219Q | MUT | nCFD |
| EZH2 | R685C | MUT | CFD | TET2 | H1219R | MUT | nCFD |
| EZH2 | R685H | MUT | CFD | TET2 | H1380L | MUT | CFD |
| EZH2 | S380T | MUT | nCFD | TET2 | H1380R | MUT | CFD |
| EZH2 | S664G | MUT | CFD | TET2 | H1382Y | MUT | CFD |
| EZH2 | S664R | MUT | CFD | TET2 | H1792Y | MUT | CFD |
| EZH2 | S690L | MUT | CFD | TET2 | H1868Y | MUT | CFD |
| EZH2 | S690P | MUT | CFD | TET2 | H1881P | MUT | CFD |
| EZH2 | T261N | MUT | nCFD | TET2 | H1881Y | MUT | CFD |
| EZH2 | T568I | MUT | nCFD | TET2 | H1904R | MUT | CFD |
| EZH2 | T678I | MUT | CFD | TET2 | H434Y | MUT | nCFD |
| EZH2 | V442D | MUT | nCFD | TET2 | H937D | MUT | nCFD |
| EZH2 | V621M | MUT | nCFD | TET2 | I1175F | MUT | nCFD |
| EZH2 | V674M | MUT | CFD | TET2 | I1762V | MUT | CFD |
| EZH2 | W624R | MUT | CFD | TET2 | I1871S | MUT | CFD |
| EZH2 | Y153C | MUT | nCFD | TET2 | I1873T | MUT | CFD |
| EZH2 | Y641C | MUT | CFD | TET2 | K110R | MUT | nCFD |
| EZH2 | Y641F | MUT | CFD | TET2 | K1197R | MUT | nCFD |
| EZH2 | Y641H | MUT | CFD | TET2 | K1243R | MUT | nCFD |
| EZH2 | Y641N | MUT | CFD | TET2 | K1310T | MUT | CFD |
| EZH2 | Y641S | MUT | CFD | TET2 | K1491R | MUT | CFD |
| EZH2 | Y726D | MUT | CFD | TET2 | K504N | MUT | nCFD |
| EZH2 | Y726N | MUT | CFD | TET2 | L103V | MUT | nCFD |
| EZH2 | A482T | SNP | nCFD | TET2 | L1210P | MUT | nCFD |
| EZH2 | D185H | SNP | nCFD | TET2 | L1229R | MUT | nCFD |
| EZH2 | D202N | SNP | nCFD | TET2 | L1231P | MUT | nCFD |
| EZH2 | H129Y | SNP | nCFD | TET2 | L1248H | MUT | nCFD |
| EZH2 | I476L | SNP | nCFD | TET2 | L1248P | MUT | nCFD |
| EZH2 | I55M | SNP | CFD | TET2 | L1322Q | MUT | CFD |
| EZH2 | P347L | SNP | nCFD | TET2 | L1322R | MUT | CFD |
| EZH2 | Q250E | SNP | nCFD | TET2 | L1329P | MUT | CFD |
| EZH2 | T378I | SNP | nCFD | TET2 | L1329R | MUT | CFD |
| JAK2 | A29T | MUT | nCFD | TET2 | L1332P | MUT | CFD |
| JAK2 | D544G | MUT | nCFD | TET2 | L1340R | MUT | CFD |
| JAK2 | D869G | MUT | CFD | TET2 | L1398R | MUT | CFD |
| JAK2 | D873N | MUT | CFD | TET2 | L1609M | MUT | CFD |
| JAK2 | E592K | MUT | CFD | TET2 | L1801F | MUT | CFD |
| JAK2 | F240L | MUT | nCFD | TET2 | L1899P | MUT | CFD |
| JAK2 | G281D | MUT | nCFD | TET2 | L307R | MUT | nCFD |
| JAK2 | G571S | MUT | CFD | TET2 | L346P | MUT | nCFD |
| JAK2 | G861W | MUT | CFD | TET2 | L541P | MUT | nCFD |
| JAK2 | I288V | MUT | nCFD | TET2 | L615F | MUT | nCFD |
| JAK2 | I682F | MUT | CFD | TET2 | M1164R | MUT | nCFD |
| JAK2 | K1055R | MUT | CFD | TET2 | M1907K | MUT | nCFD |
| JAK2 | K539E | MUT | nCFD | TET2 | M638V | MUT | nCFD |
| JAK2 | K539L | MUT | nCFD | TET2 | N1266H | MUT | nCFD |
| JAK2 | K607N | MUT | CFD | TET2 | N1266Y | MUT | nCFD |
| JAK2 | K728E | MUT | CFD | TET2 | N1387I | MUT | CFD |

| | | | | | | | |
|---|---|---|---|---|---|---|---|
| JAK2 | L224W | MUT | nCFD | TET2 | N1387S | MUT | CFD |
| JAK2 | L545S | MUT | CFD | TET2 | N1641I | MUT | CFD |
| JAK2 | L579F | MUT | CFD | TET2 | N1890S | MUT | CFD |
| JAK2 | L611S | MUT | CFD | TET2 | N275S | MUT | nCFD |
| JAK2 | L611V | MUT | CFD | TET2 | N312S | MUT | nCFD |
| JAK2 | N1108S | MUT | CFD | TET2 | P101H | MUT | nCFD |
| JAK2 | N249K | MUT | nCFD | TET2 | P1115H | MUT | nCFD |
| JAK2 | P933R | MUT | CFD | TET2 | P1194L | MUT | nCFD |
| JAK2 | R122H | MUT | nCFD | TET2 | P1367R | MUT | CFD |
| JAK2 | R487C | MUT | nCFD | TET2 | P1419R | MUT | CFD |
| JAK2 | R683G | MUT | CFD | TET2 | P174H | MUT | nCFD |
| JAK2 | R683K | MUT | CFD | TET2 | P1857S | MUT | CFD |
| JAK2 | R683S | MUT | CFD | TET2 | P1889L | MUT | CFD |
| JAK2 | R683T | MUT | CFD | TET2 | P1894L | MUT | CFD |
| JAK2 | R715T | MUT | CFD | TET2 | P1894T | MUT | CFD |
| JAK2 | R867Q | MUT | CFD | TET2 | P1962L | MUT | nCFD |
| JAK2 | R923C | MUT | CFD | TET2 | P22L | MUT | nCFD |
| JAK2 | R923H | MUT | CFD | TET2 | P874A | MUT | nCFD |
| JAK2 | S367P | MUT | nCFD | TET2 | Q108L | MUT | nCFD |
| JAK2 | S398T | MUT | nCFD | TET2 | Q1274R | MUT | nCFD |
| JAK2 | S797C | MUT | CFD | TET2 | Q1348P | MUT | CFD |
| JAK2 | T108S | MUT | nCFD | TET2 | Q548L | MUT | nCFD |
| JAK2 | T875N | MUT | CFD | TET2 | R1167M | MUT | nCFD |
| JAK2 | V617F | MUT | CFD | TET2 | R1179G | MUT | nCFD |
| JAK2 | C480F | SNP | CFD | TET2 | R1214Q | MUT | nCFD |
| JAK2 | D1096E | SNP | CFD | TET2 | R1214W | MUT | nCFD |
| JAK2 | D194A | SNP | nCFD | TET2 | R1261C | MUT | nCFD |
| JAK2 | D519Y | SNP | nCFD | TET2 | R1261H | MUT | nCFD |
| JAK2 | D789E | SNP | CFD | TET2 | R1261S | MUT | nCFD |
| JAK2 | E577K | SNP | CFD | TET2 | R1262W | MUT | nCFD |
| JAK2 | E846D | SNP | nCFD | TET2 | R1359C | MUT | CFD |
| JAK2 | E890K | SNP | CFD | TET2 | R1359H | MUT | CFD |
| JAK2 | G294S | SNP | nCFD | TET2 | R1383G | MUT | CFD |
| JAK2 | G417S | SNP | CFD | TET2 | R1896M | MUT | CFD |
| JAK2 | G48E | SNP | nCFD | TET2 | R1896S | MUT | CFD |
| JAK2 | H886R | SNP | CFD | TET2 | R2000K | MUT | nCFD |
| JAK2 | I136L | SNP | nCFD | TET2 | R814C | MUT | nCFD |
| JAK2 | I19V | SNP | nCFD | TET2 | S1190Y | MUT | nCFD |
| JAK2 | I223T | SNP | nCFD | TET2 | S1203R | MUT | nCFD |
| JAK2 | I354T | SNP | nCFD | TET2 | S1290P | MUT | CFD |
| JAK2 | I899S | SNP | CFD | TET2 | S1292R | MUT | CFD |
| JAK2 | K244R | SNP | nCFD | TET2 | S1392R | MUT | CFD |
| JAK2 | K253R | SNP | nCFD | TET2 | S1563F | MUT | CFD |
| JAK2 | K639R | SNP | CFD | TET2 | S1870P | MUT | CFD |
| JAK2 | K883R | SNP | CFD | TET2 | S1898F | MUT | CFD |
| JAK2 | L113V | SNP | nCFD | TET2 | S282F | MUT | nCFD |
| JAK2 | L393V | SNP | nCFD | TET2 | S460F | MUT | nCFD |
| JAK2 | L732S | SNP | CFD | TET2 | S521N | MUT | nCFD |
| JAK2 | L830V | SNP | nCFD | TET2 | S774T | MUT | nCFD |
| JAK2 | L892V | SNP | CFD | TET2 | S826I | MUT | nCFD |
| JAK2 | N337D | SNP | nCFD | TET2 | S99T | MUT | nCFD |
| JAK2 | N479K | SNP | CFD | TET2 | T1249N | MUT | nCFD |
| JAK2 | N490S | SNP | nCFD | TET2 | T1270A | MUT | nCFD |
| JAK2 | P521L | SNP | nCFD | TET2 | T1372I | MUT | CFD |
| JAK2 | R1063H | SNP | CFD | TET2 | T1393A | MUT | CFD |
| JAK2 | S1115C | SNP | CFD | TET2 | T1393I | MUT | CFD |
| JAK2 | S15F | SNP | nCFD | TET2 | T1883R | MUT | CFD |
| JAK2 | S797P | SNP | CFD | TET2 | T1884A | MUT | CFD |

| | | | | | | | |
|---|---|---|---|---|---|---|---|
| JAK2 | V341A | SNP | nCFD | TET2 | T1884I | MUT | CFD |
| JAK2 | V392L | SNP | nCFD | TET2 | T492S | MUT | nCFD |
| JAK2 | V392M | SNP | nCFD | TET2 | V1199I | MUT | nCFD |
| JAK2 | V567I | SNP | CFD | TET2 | V1213M | MUT | nCFD |
| JAK2 | W659R | SNP | CFD | TET2 | V1417F | MUT | CFD |
| JAK2 | Y201C | SNP | nCFD | TET2 | V1718L | MUT | CFD |
| JAK2 | Y435C | SNP | CFD | TET2 | V218M | MUT | nCFD |
| KMT2A | A1560T | MUT | nCFD | TET2 | V9I | MUT | nCFD |
| KMT2A | A3356V | MUT | nCFD | TET2 | W1233G | MUT | nCFD |
| KMT2A | D1396N | MUT | nCFD | TET2 | W1291C | MUT | CFD |
| KMT2A | D1580N | MUT | CFD | TET2 | W1291R | MUT | CFD |
| KMT2A | D2817N | MUT | nCFD | TET2 | Y1148H | MUT | nCFD |
| KMT2A | E1412K | MUT | nCFD | TET2 | Y1579S | MUT | CFD |
| KMT2A | E3013Q | MUT | nCFD | TET2 | Y1902H | MUT | CFD |
| KMT2A | G152D | MUT | nCFD | TET2 | Y867H | MUT | nCFD |
| KMT2A | H3761Q | MUT | nCFD | TET2 | A1014S | SNP | nCFD |
| KMT2A | H468N | MUT | nCFD | TET2 | A1769P | SNP | CFD |
| KMT2A | K1270N | MUT | nCFD | TET2 | A1769T | SNP | CFD |
| KMT2A | K1590R | MUT | CFD | TET2 | A308P | SNP | nCFD |
| KMT2A | K2461N | MUT | nCFD | TET2 | A575V | SNP | nCFD |
| KMT2A | K3738N | MUT | CFD | TET2 | A911D | SNP | nCFD |
| KMT2A | K853Q | MUT | nCFD | TET2 | D115E | SNP | nCFD |
| KMT2A | K895R | MUT | nCFD | TET2 | D1788G | SNP | CFD |
| KMT2A | L905M | MUT | nCFD | TET2 | E1513G | SNP | CFD |
| KMT2A | M2599V | MUT | nCFD | TET2 | E1929K | SNP | nCFD |
| KMT2A | M3931T | MUT | CFD | TET2 | F868L | SNP | nCFD |
| KMT2A | N125T | MUT | nCFD | TET2 | G1187S | SNP | nCFD |
| KMT2A | P1252H | MUT | nCFD | TET2 | G355D | SNP | nCFD |
| KMT2A | P2122S | MUT | nCFD | TET2 | G429R | SNP | nCFD |
| KMT2A | P2462T | MUT | nCFD | TET2 | H169R | SNP | nCFD |
| KMT2A | P2555L | MUT | nCFD | TET2 | H1778R | SNP | CFD |
| KMT2A | P2741A | MUT | nCFD | TET2 | H1806R | SNP | CFD |
| KMT2A | Q2513K | MUT | nCFD | TET2 | H1817N | SNP | CFD |
| KMT2A | Q408L | MUT | nCFD | TET2 | H786R | SNP | nCFD |
| KMT2A | R2376S | MUT | nCFD | TET2 | H924R | SNP | nCFD |
| KMT2A | R3225H | MUT | nCFD | TET2 | I1195V | SNP | nCFD |
| KMT2A | R3819H | MUT | nCFD | TET2 | I1762L | SNP | CFD |
| KMT2A | S2408N | MUT | nCFD | TET2 | I1873S | SNP | CFD |
| KMT2A | S3036Y | MUT | nCFD | TET2 | I921V | SNP | nCFD |
| KMT2A | T3075S | MUT | nCFD | TET2 | L103P | SNP | nCFD |
| KMT2A | V3862I | MUT | CFD | TET2 | L1120M | SNP | nCFD |
| KMT2A | A2332V | SNP | nCFD | TET2 | L1721W | SNP | CFD |
| KMT2A | A30G | SNP | nCFD | TET2 | L1816F | SNP | CFD |
| KMT2A | A3299T | SNP | nCFD | TET2 | L34F | SNP | nCFD |
| KMT2A | A3313V | SNP | nCFD | TET2 | L567S | SNP | nCFD |
| KMT2A | A3422V | SNP | nCFD | TET2 | M1701I | SNP | CFD |
| KMT2A | A3440T | SNP | nCFD | TET2 | N1567S | SNP | CFD |
| KMT2A | A3489T | SNP | nCFD | TET2 | N1581D | SNP | CFD |
| KMT2A | A53V | SNP | nCFD | TET2 | N1616S | SNP | CFD |
| KMT2A | C3427G | SNP | nCFD | TET2 | N1746H | SNP | CFD |
| KMT2A | D1251V | SNP | nCFD | TET2 | N275K | SNP | nCFD |
| KMT2A | D3394A | SNP | nCFD | TET2 | N767D | SNP | nCFD |
| KMT2A | E2694D | SNP | nCFD | TET2 | P1723S | SNP | CFD |
| KMT2A | E502K | SNP | nCFD | TET2 | P1770L | SNP | CFD |
| KMT2A | E502Q | SNP | nCFD | TET2 | P29R | SNP | nCFD |
| KMT2A | E919D | SNP | nCFD | TET2 | P363L | SNP | nCFD |
| KMT2A | G1065V | SNP | nCFD | TET2 | P472A | SNP | nCFD |
| KMT2A | G2349S | SNP | nCFD | TET2 | P474L | SNP | nCFD |

| | | | | | | | |
|---|---|---|---|---|---|---|---|
| KMT2A | G3128S | SNP | nCFD | TET2 | P507R | SNP | nCFD |
| KMT2A | G3513E | SNP | nCFD | TET2 | P555L | SNP | nCFD |
| KMT2A | G76A | SNP | nCFD | TET2 | P761L | SNP | nCFD |
| KMT2A | H2993R | SNP | nCFD | TET2 | Q1084P | SNP | nCFD |
| KMT2A | I1642V | SNP | nCFD | TET2 | Q232R | SNP | nCFD |
| KMT2A | I3437V | SNP | nCFD | TET2 | Q591R | SNP | nCFD |
| KMT2A | I3569S | SNP | nCFD | TET2 | R1095K | SNP | nCFD |
| KMT2A | K3101E | SNP | nCFD | TET2 | R1366H | SNP | CFD |
| KMT2A | K398R | SNP | nCFD | TET2 | R1404Q | SNP | CFD |
| KMT2A | K860R | SNP | nCFD | TET2 | R1572Q | SNP | CFD |
| KMT2A | L3274F | SNP | nCFD | TET2 | R369Q | SNP | nCFD |
| KMT2A | L3614P | SNP | nCFD | TET2 | R369W | SNP | nCFD |
| KMT2A | L989F | SNP | nCFD | TET2 | R5T | SNP | nCFD |
| KMT2A | M1788I | SNP | nCFD | TET2 | S1039L | SNP | nCFD |
| KMT2A | M1923I | SNP | CFD | TET2 | S1205T | SNP | nCFD |
| KMT2A | M2213V | SNP | nCFD | TET2 | S145N | SNP | nCFD |
| KMT2A | M604V | SNP | nCFD | TET2 | S1611Y | SNP | CFD |
| KMT2A | N1811T | SNP | nCFD | TET2 | S602C | SNP | nCFD |
| KMT2A | N3754S | SNP | nCFD | TET2 | S689A | SNP | nCFD |
| KMT2A | P1354H | SNP | nCFD | TET2 | S795R | SNP | nCFD |
| KMT2A | P1367L | SNP | nCFD | TET2 | S890L | SNP | nCFD |
| KMT2A | P2077L | SNP | nCFD | TET2 | V1978M | SNP | nCFD |
| KMT2A | P2161L | SNP | nCFD | TET2 | Y192H | SNP | nCFD |
| KMT2A | P2170S | SNP | nCFD | TET2 | Y559C | SNP | nCFD |
| KMT2A | P3528A | SNP | nCFD | TP53 | C135F | MUT | CFD |
| KMT2A | P3533S | SNP | nCFD | TP53 | C135R | MUT | CFD |
| KMT2A | P3533T | SNP | nCFD | TP53 | C135S | MUT | CFD |
| KMT2A | P507S | SNP | nCFD | TP53 | C135W | MUT | CFD |
| KMT2A | P562S | SNP | nCFD | TP53 | C141G | MUT | CFD |
| KMT2A | P562T | SNP | nCFD | TP53 | C141W | MUT | CFD |
| KMT2A | Q1761H | SNP | nCFD | TP53 | C141Y | MUT | CFD |
| KMT2A | Q1975P | SNP | CFD | TP53 | C176F | MUT | CFD |
| KMT2A | Q2387R | SNP | nCFD | TP53 | C176G | MUT | CFD |
| KMT2A | Q3083H | SNP | nCFD | TP53 | C176S | MUT | CFD |
| KMT2A | Q3598H | SNP | nCFD | TP53 | C176Y | MUT | CFD |
| KMT2A | R1502Q | SNP | CFD | TP53 | C238S | MUT | CFD |
| KMT2A | R1627Q | SNP | CFD | TP53 | C238Y | MUT | CFD |
| KMT2A | R2188Q | SNP | nCFD | TP53 | C242F | MUT | CFD |
| KMT2A | R2516Q | SNP | nCFD | TP53 | C242R | MUT | CFD |
| KMT2A | R3561W | SNP | nCFD | TP53 | C275F | MUT | CFD |
| KMT2A | S1325N | SNP | nCFD | TP53 | C275R | MUT | CFD |
| KMT2A | S1337L | SNP | nCFD | TP53 | C275Y | MUT | CFD |
| KMT2A | S2319T | SNP | nCFD | TP53 | C277F | MUT | CFD |
| KMT2A | S2432C | SNP | nCFD | TP53 | C277S | MUT | CFD |
| KMT2A | S252I | SNP | nCFD | TP53 | D259Y | MUT | CFD |
| KMT2A | S2831P | SNP | nCFD | TP53 | D281E | MUT | CFD |
| KMT2A | S3107P | SNP | nCFD | TP53 | D281N | MUT | CFD |
| KMT2A | S3172G | SNP | nCFD | TP53 | D281Y | MUT | CFD |
| KMT2A | S3178I | SNP | nCFD | TP53 | D48N | MUT | nCFD |
| KMT2A | S3178N | SNP | nCFD | TP53 | E224D | MUT | CFD |
| KMT2A | S3481Y | SNP | nCFD | TP53 | E258D | MUT | CFD |
| KMT2A | S3659G | SNP | nCFD | TP53 | E271K | MUT | CFD |
| KMT2A | S3702T | SNP | CFD | TP53 | E285K | MUT | CFD |
| KMT2A | S3710A | SNP | CFD | TP53 | E286K | MUT | CFD |
| KMT2A | S487P | SNP | nCFD | TP53 | F134C | MUT | CFD |
| KMT2A | S779L | SNP | nCFD | TP53 | F134L | MUT | CFD |
| KMT2A | S783C | SNP | nCFD | TP53 | F134V | MUT | CFD |
| KMT2A | S830N | SNP | nCFD | TP53 | G105V | MUT | CFD |

| | | | | | | | |
|---|---|---|---|---|---|---|---|
| KMT2A | S830T | SNP | nCFD | TP53 | G108S | MUT | CFD |
| KMT2A | S96L | SNP | nCFD | TP53 | G187D | MUT | CFD |
| KMT2A | T1245I | SNP | nCFD | TP53 | G187S | MUT | CFD |
| KMT2A | T2230I | SNP | nCFD | TP53 | G199E | MUT | CFD |
| KMT2A | T3210I | SNP | nCFD | TP53 | G244C | MUT | CFD |
| KMT2A | T3318I | SNP | nCFD | TP53 | G244D | MUT | CFD |
| KMT2A | T3463A | SNP | nCFD | TP53 | G244S | MUT | CFD |
| KMT2A | T993I | SNP | nCFD | TP53 | G245C | MUT | CFD |
| KMT2A | V2310A | SNP | nCFD | TP53 | G245D | MUT | CFD |
| KMT2A | V2772A | SNP | nCFD | TP53 | G245S | MUT | CFD |
| KMT2A | V3198A | SNP | nCFD | TP53 | G262R | MUT | CFD |
| KMT2A | V3714I | SNP | CFD | TP53 | G262V | MUT | CFD |
| KMT2A | V484A | SNP | nCFD | TP53 | G266E | MUT | CFD |
| KMT2A | V484I | SNP | nCFD | TP53 | G266V | MUT | CFD |
| KMT2A | V498I | SNP | nCFD | TP53 | H168R | MUT | CFD |
| KMT2C | A2456T | MUT | nCFD | TP53 | H178P | MUT | CFD |
| KMT2C | A4446T | MUT | CFD | TP53 | H179D | MUT | CFD |
| KMT2C | A803P | MUT | nCFD | TP53 | H179L | MUT | CFD |
| KMT2C | C1114R | MUT | nCFD | TP53 | H179Q | MUT | CFD |
| KMT2C | C4503R | MUT | CFD | TP53 | H179Y | MUT | CFD |
| KMT2C | C4503W | MUT | CFD | TP53 | H193L | MUT | CFD |
| KMT2C | D149V | MUT | nCFD | TP53 | H193P | MUT | CFD |
| KMT2C | D2092V | MUT | nCFD | TP53 | H193R | MUT | CFD |
| KMT2C | D2673G | MUT | nCFD | TP53 | H193Y | MUT | CFD |
| KMT2C | D2713N | MUT | nCFD | TP53 | H214R | MUT | CFD |
| KMT2C | D2714N | MUT | nCFD | TP53 | I162N | MUT | CFD |
| KMT2C | D348N | MUT | nCFD | TP53 | I195F | MUT | CFD |
| KMT2C | D738E | MUT | nCFD | TP53 | I195T | MUT | CFD |
| KMT2C | E1253A | MUT | nCFD | TP53 | I232F | MUT | CFD |
| KMT2C | E2698K | MUT | nCFD | TP53 | I251N | MUT | CFD |
| KMT2C | E2885K | MUT | nCFD | TP53 | I255F | MUT | CFD |
| KMT2C | E4319K | MUT | nCFD | TP53 | I332S | MUT | CFD |
| KMT2C | E864G | MUT | nCFD | TP53 | K120E | MUT | CFD |
| KMT2C | F1753L | MUT | nCFD | TP53 | K132E | MUT | CFD |
| KMT2C | G1624R | MUT | nCFD | TP53 | K132Q | MUT | CFD |
| KMT2C | G1815V | MUT | nCFD | TP53 | K139N | MUT | CFD |
| KMT2C | G2041R | MUT | nCFD | TP53 | K164E | MUT | CFD |
| KMT2C | G315S | MUT | CFD | TP53 | L111R | MUT | CFD |
| KMT2C | G4074S | MUT | nCFD | TP53 | L130P | MUT | CFD |
| KMT2C | G4802V | MUT | CFD | TP53 | L130V | MUT | CFD |
| KMT2C | G639C | MUT | nCFD | TP53 | L145R | MUT | CFD |
| KMT2C | G845E | MUT | nCFD | TP53 | L188V | MUT | CFD |
| KMT2C | G892R | MUT | nCFD | TP53 | L194R | MUT | CFD |
| KMT2C | H290N | MUT | CFD | TP53 | L330P | MUT | CFD |
| KMT2C | H3205Y | MUT | nCFD | TP53 | M133R | MUT | CFD |
| KMT2C | I2756V | MUT | nCFD | TP53 | M237I | MUT | CFD |
| KMT2C | I3590L | MUT | nCFD | TP53 | M237L | MUT | CFD |
| KMT2C | K3889Q | MUT | nCFD | TP53 | M243T | MUT | CFD |
| KMT2C | L224P | MUT | nCFD | TP53 | M246I | MUT | CFD |
| KMT2C | L2387V | MUT | nCFD | TP53 | M246K | MUT | CFD |
| KMT2C | L3116P | MUT | nCFD | TP53 | M246L | MUT | CFD |
| KMT2C | L901P | MUT | nCFD | TP53 | M246R | MUT | CFD |
| KMT2C | M1974I | MUT | nCFD | TP53 | M246V | MUT | CFD |
| KMT2C | M2304V | MUT | nCFD | TP53 | N235S | MUT | CFD |
| KMT2C | M796V | MUT | nCFD | TP53 | N239D | MUT | CFD |
| KMT2C | N1563D | MUT | nCFD | TP53 | N239T | MUT | CFD |
| KMT2C | N2088I | MUT | nCFD | TP53 | N345I | MUT | CFD |
| KMT2C | N2106I | MUT | nCFD | TP53 | P152L | MUT | CFD |

| | | | | | | | |
|---|---|---|---|---|---|---|---|
| KMT2C | N2830H | MUT | nCFD | TP53 | P278L | MUT | CFD |
| KMT2C | N3347S | MUT | nCFD | TP53 | P278R | MUT | CFD |
| KMT2C | P1080L | MUT | nCFD | TP53 | P58Q | MUT | nCFD |
| KMT2C | P1544S | MUT | nCFD | TP53 | P82L | MUT | nCFD |
| KMT2C | P157L | MUT | nCFD | TP53 | Q136H | MUT | CFD |
| KMT2C | P1606L | MUT | nCFD | TP53 | R110L | MUT | CFD |
| KMT2C | P2012S | MUT | nCFD | TP53 | R158H | MUT | CFD |
| KMT2C | P2050S | MUT | nCFD | TP53 | R175G | MUT | CFD |
| KMT2C | P2278L | MUT | nCFD | TP53 | R175H | MUT | CFD |
| KMT2C | P2278S | MUT | nCFD | TP53 | R181H | MUT | CFD |
| KMT2C | P2450S | MUT | nCFD | TP53 | R181P | MUT | CFD |
| KMT2C | P2616S | MUT | nCFD | TP53 | R181S | MUT | CFD |
| KMT2C | P309S | MUT | CFD | TP53 | R196P | MUT | CFD |
| KMT2C | P3452L | MUT | nCFD | TP53 | R213Q | MUT | CFD |
| KMT2C | P3513L | MUT | nCFD | TP53 | R248L | MUT | CFD |
| KMT2C | P3583L | MUT | nCFD | TP53 | R248W | MUT | CFD |
| KMT2C | P837T | MUT | nCFD | TP53 | R249M | MUT | CFD |
| KMT2C | Q2680H | MUT | nCFD | TP53 | R249S | MUT | CFD |
| KMT2C | Q3792K | MUT | nCFD | TP53 | R267Q | MUT | CFD |
| KMT2C | Q588H | MUT | nCFD | TP53 | R273C | MUT | CFD |
| KMT2C | Q608R | MUT | nCFD | TP53 | R273G | MUT | CFD |
| KMT2C | R164K | MUT | nCFD | TP53 | R273H | MUT | CFD |
| KMT2C | R2388C | MUT | nCFD | TP53 | R273S | MUT | CFD |
| KMT2C | R254C | MUT | CFD | TP53 | R280G | MUT | CFD |
| KMT2C | R2610Q | MUT | nCFD | TP53 | R280T | MUT | CFD |
| KMT2C | R3177H | MUT | nCFD | TP53 | R282G | MUT | CFD |
| KMT2C | R3400H | MUT | nCFD | TP53 | R282Q | MUT | CFD |
| KMT2C | R3403H | MUT | nCFD | TP53 | R282W | MUT | CFD |
| KMT2C | R380P | MUT | nCFD | TP53 | R283C | MUT | CFD |
| KMT2C | R3960Q | MUT | nCFD | TP53 | R290H | MUT | nCFD |
| KMT2C | R4584Q | MUT | CFD | TP53 | R333C | MUT | CFD |
| KMT2C | R4822H | MUT | CFD | TP53 | R337C | MUT | CFD |
| KMT2C | R4907Q | MUT | nCFD | TP53 | S127P | MUT | CFD |
| KMT2C | R894W | MUT | nCFD | TP53 | S215R | MUT | CFD |
| KMT2C | S1307A | MUT | nCFD | TP53 | S240G | MUT | CFD |
| KMT2C | S2095G | MUT | nCFD | TP53 | S241A | MUT | CFD |
| KMT2C | S2095I | MUT | nCFD | TP53 | S241C | MUT | CFD |
| KMT2C | S2508F | MUT | nCFD | TP53 | S241P | MUT | CFD |
| KMT2C | S2869T | MUT | nCFD | TP53 | S241T | MUT | CFD |
| KMT2C | S3588L | MUT | nCFD | TP53 | S261T | MUT | CFD |
| KMT2C | S4190T | MUT | nCFD | TP53 | S46F | MUT | nCFD |
| KMT2C | S730Y | MUT | nCFD | TP53 | S99F | MUT | CFD |
| KMT2C | S990G | MUT | nCFD | TP53 | T118I | MUT | CFD |
| KMT2C | T2100I | MUT | nCFD | TP53 | T125R | MUT | CFD |
| KMT2C | T3498I | MUT | nCFD | TP53 | T150A | MUT | CFD |
| KMT2C | T820I | MUT | nCFD | TP53 | T155N | MUT | CFD |
| KMT2C | V2322A | MUT | nCFD | TP53 | T18A | MUT | CFD |
| KMT2C | V3661M | MUT | nCFD | TP53 | T377P | MUT | nCFD |
| KMT2C | V4204I | MUT | nCFD | TP53 | V143M | MUT | CFD |
| KMT2C | V655I | MUT | nCFD | TP53 | V147G | MUT | CFD |
| KMT2C | V920L | MUT | nCFD | TP53 | V157F | MUT | CFD |
| KMT2C | Y2094F | MUT | nCFD | TP53 | V157G | MUT | CFD |
| KMT2C | Y2145C | MUT | nCFD | TP53 | V172D | MUT | CFD |
| KMT2C | A1685S | SNP | nCFD | TP53 | V173L | MUT | CFD |
| KMT2C | A1685T | SNP | nCFD | TP53 | V197E | MUT | CFD |
| KMT2C | A2223T | SNP | nCFD | TP53 | V197G | MUT | CFD |
| KMT2C | A241G | SNP | nCFD | TP53 | V216M | MUT | CFD |
| KMT2C | A3616V | SNP | nCFD | TP53 | V272M | MUT | CFD |

| | | | | | | | |
|---|---|---|---|---|---|---|---|
| KMT2C | A3723P | SNP | nCFD | TP53 | V274L | MUT | CFD |
| KMT2C | A3748T | SNP | nCFD | TP53 | W23C | MUT | CFD |
| KMT2C | A3921V | SNP | nCFD | TP53 | Y107D | MUT | CFD |
| KMT2C | A3930D | SNP | nCFD | TP53 | Y126C | MUT | CFD |
| KMT2C | A3930V | SNP | nCFD | TP53 | Y126D | MUT | CFD |
| KMT2C | A4252G | SNP | nCFD | TP53 | Y163C | MUT | CFD |
| KMT2C | A4252V | SNP | nCFD | TP53 | Y163D | MUT | CFD |
| KMT2C | A4709S | SNP | nCFD | TP53 | Y205H | MUT | CFD |
| KMT2C | A846V | SNP | nCFD | TP53 | Y205N | MUT | CFD |
| KMT2C | C1953S | SNP | nCFD | TP53 | Y220C | MUT | CFD |
| KMT2C | C394Y | SNP | CFD | TP53 | Y220H | MUT | CFD |
| KMT2C | D1319H | SNP | nCFD | TP53 | Y234C | MUT | CFD |
| KMT2C | D1450V | SNP | nCFD | TP53 | Y234H | MUT | CFD |
| KMT2C | D1800E | SNP | nCFD | TP53 | Y234N | MUT | CFD |
| KMT2C | D2092G | SNP | nCFD | TP53 | Y236C | MUT | CFD |
| KMT2C | D2461N | SNP | nCFD | TP53 | Y236H | MUT | CFD |
| KMT2C | D3264G | SNP | nCFD | TP53 | Y236N | MUT | CFD |
| KMT2C | D3841V | SNP | nCFD | TP53 | E339K | SNP | CFD |
| KMT2C | D3990N | SNP | nCFD | TP53 | E339Q | SNP | CFD |
| KMT2C | D4393N | SNP | nCFD | TP53 | G262C | SNP | CFD |
| KMT2C | D4790H | SNP | CFD | TP53 | G262S | SNP | CFD |
| KMT2C | D525A | SNP | nCFD | TP53 | N263D | SNP | CFD |
| KMT2C | D525G | SNP | nCFD | TP53 | P47S | SNP | nCFD |
| KMT2C | D599Y | SNP | nCFD | TP53 | P47T | SNP | nCFD |
| KMT2C | D958H | SNP | nCFD | TP53 | P72H | SNP | nCFD |
| KMT2C | E2656K | SNP | nCFD | TP53 | P72R | SNP | nCFD |
| KMT2C | E2834D | SNP | nCFD | TP53 | R110H | SNP | CFD |
| KMT2C | E3872K | SNP | nCFD | TP53 | R110P | SNP | CFD |
| KMT2C | E672D | SNP | nCFD | TP53 | R175C | SNP | CFD |
| KMT2C | E765G | SNP | nCFD | TP53 | R333H | SNP | CFD |
| KMT2C | F2482S | SNP | nCFD | TP53 | S366A | SNP | nCFD |
| KMT2C | F3171L | SNP | nCFD | TP53 | V10I | SNP | CFD |
| KMT2C | F3831C | SNP | nCFD | TP53 | V10L | SNP | CFD |
| KMT2C | F835L | SNP | nCFD | TP53 | V217M | SNP | CFD |
| KMT2C | G1789A | SNP | nCFD | TP53 | V31F | SNP | nCFD |
| KMT2C | G1789C | SNP | nCFD | TP53 | V31I | SNP | nCFD |
| KMT2C | G1789V | SNP | nCFD | | | | |

Abbreviations in the table: AAS – Amino Acid Substitution, CFD – Conserved Functional Domain, nCFD – not Conserved Functional Domain

Supplementary Table 2. Features selected using Naïve Bayes machine learning algorithm for each gene (GSM approach) in the variant dataset.

| Gene | Selected features – AAIndex ID | Selected features- Description |
|---|---|---|
| ARID1A | CHAM830108<br>FASG760104<br>FAUJ880105<br>FAUJ880108<br>GEIM800103<br>MAXF760104<br><br>QIAN880103<br><br>QIAN880113 | A parameter of charge transfer donor capability (Charton-Charton, 1983)<br>pK-N (Fasman, 1976)<br>STERIMOL minimum width of the side chain (Fauchere et al., 1988)<br>Localized electrical effect (Fauchere et al., 1988)<br>Alpha-helix indices for beta-proteins (Geisow-Roberts, 1980)<br>Normalized frequency of left-handed alpha-helix (Maxfield-Scheraga, 1976)<br>Weights for alpha-helix at the window position of -4 (Qian-Sejnowski, 1988)<br>Weights for alpha-helix at the window position of 6 (Qian-Sejnowski, |

| | | |
|---|---|---|
| | | 1988) |
| | QIAN880122 | Weights for beta-sheet at the window position of 2 (Qian-Sejnowski, 1988) |
| | RACS820104 | Average relative fractional occurrence in EL(i) (Rackovsky-Scheraga, 1982) |
| | AURR980105 | Normalized positional residue frequency at helix termini Nc (Aurora-Rose, 1998) |
| | AURR980119 | Normalized positional residue frequency at helix termini C''' (Aurora-Rose, 1998) |
| | AURR980120 | Normalized positional residue frequency at helix termini C4' (Aurora-Rose, 1998) |
| | MONM990201 | Averaged turn propensities in a transmembrane helix (Monne et al., 1999) |
| | GEOR030102 | Linker propensity from 1-linker dataset (George-Heringa, 2003) |
| | GEOR030105 | Linker propensity from small dataset (linker length is less than six residues) (George-Heringa, 2003) |
| | GEOR030107 | Linker propensity from long dataset (linker length is greater than 14 residues) (George-Heringa, 2003) |
| | GEOR030109 | Linker propensity from non-helical (annotated by DSSP) dataset (George-Heringa, 2003) |
| | KARS160103 | Total weighted degree of the graph (obtained by adding all the weights of all the vertices) (Karkbara-Knisley, 2016) |
| | KARS160107 | Diameter (maximum eccentricity) (Karkbara-Knisley, 2016) |
| | KARS160113 | Weighted domination number using the atomic number (Karkbara-Knisley, 2016) |
| | KARS160114 | Average weighted eccentricity based on the the atomic number (Karkbara-Knisley, 2016) |
| | KARS160115 | Weighted radius based on the atomic number (minimum eccentricity) (Karkbara-Knisley, 2016) |
| | KARS160117 | Total weighted atomic number of the graph (obtained by summing all the atomic number of each of the vertices in the graph) (Karkbara-Knisley, 2016) |
| ASXL1 | GARJ730101 | Partition coefficient (Garel et al., 1973) |
| | KLEP840101 | Net charge (Klein et al., 1984) |
| | NAKH900112 | Transmembrane regions of mt-proteins (Nakashima et al., 1990) |
| | PRAM820103 | Correlation coefficient in regression analysis (Prabhakaran-Ponnuswamy, 1982) |
| | PRAM900101 | Hydrophobicity (Prabhakaran, 1990) |
| | QIAN880114 | Weights for beta-sheet at the window position of -6 (Qian-Sejnowski, 1988) |
| | QIAN880117 | Weights for beta-sheet at the window position of -3 (Qian-Sejnowski, 1988) |
| | QIAN880126 | Weights for beta-sheet at the window position of 6 (Qian-Sejnowski, 1988) |
| | RACS820102 | Average relative fractional occurrence in AR(i) (Rackovsky-Scheraga, 1982) |
| | VENT840101 | Bitterness (Venanzi, 1984) |
| | WOLS870101 | Principal property value z1 (Wold et al., 1987) |
| | ZASB820101 | Dependence of partition coefficient on ionic strength (Zaslavsky et al., 1982) |
| | ZIMJ680102 | Bulkiness (Zimmerman et al., 1968) |
| | ZIMJ680103 | Polarity (Zimmerman et al., 1968) |
| | AURR980105 | Normalized positional residue frequency at helix termini Nc (Aurora-Rose, 1998) |
| | AURR980107 | Normalized positional residue frequency at helix termini N2 (Aurora-Rose, 1998) |
| | AURR980110 | Normalized positional residue frequency at helix termini N5 (Aurora-Rose, 1998) |
| | FUKS010102 | Surface composition of amino acids in intracellular proteins of mesophiles (percent) (Fukuchi-Nishikawa, 2001) |
| | WILM950102 | Hydrophobicity coefficient in RP-HPLC, C8 with 0.1%TFA/MeCN/H2O |

| | | |
|---|---|---|
| | | (Wilce et al. 1995) |
| | WILM950103 | Hydrophobicity coefficient in RP-HPLC, C4 with 0.1%TFA/MeCN/H2O (Wilce et al. 1995) |
| | ENGD860101 | Hydrophobicity index (Engelman et al., 1986) |
| | KARS160118 | Average weighted atomic number or degree based on atomic number in the graph (Karkbara-Knisley, 2016) |
| | KARS160121 | Weighted average eigenvalue based on the atomic numbers (Karkbara-Knisley, 2016) |
| ATM | BHAR880101 | Average flexibility indices (Bhaskaran-Ponnuswamy, 1988) |
| | BURA740102 | Normalized frequency of extended structure (Burgess et al., 1974) |
| | CHAM830103 | The number of atoms in the side chain labelled 1+1 (Charton-Charton, 1983) |
| | CHAM830107 | A parameter of charge transfer capability (Charton-Charton, 1983) |
| | CHOC760102 | Residue accessible surface area in folded protein (Chothia, 1976) |
| | CHOC760103 | Proportion of residues 95% buried (Chothia, 1976) |
| | CHOP780209 | Normalized frequency of C-terminal beta-sheet (Chou-Fasman, 1978b) |
| | CHOP780211 | Normalized frequency of C-terminal non beta region (Chou-Fasman, 1978b) |
| | CHOP780215 | Frequency of the 4th residue in turn (Chou-Fasman, 1978b) |
| | CIDH920102 | Normalized hydrophobicity scales for beta-proteins (Cid et al., 1992) |
| | DESM900101 | Membrane preference for cytochrome b: MPH89 (Degli Esposti et al., 1990) |
| | FASG760103 | Optical rotation (Fasman, 1976) |
| | FAUJ880111 | Positive charge (Fauchere et al., 1988) |
| | FAUJ880112 | Negative charge (Fauchere et al., 1988) |
| | FINA910101 | Helix initiation parameter at posision i-1 (Finkelstein et al., 1991) |
| | GEIM800101 | Alpha-helix indices (Geisow-Roberts, 1980) |
| | GRAR740103 | Volume (Grantham, 1974) |
| | ISOY800103 | Normalized relative frequency of bend (Isogai et al., 1980) |
| | ISOY800105 | Normalized relative frequency of bend S (Isogai et al., 1980) |
| | ISOY800106 | Normalized relative frequency of helix end (Isogai et al., 1980) |
| | JANJ790101 | Ratio of buried and accessible molar fractions (Janin, 1979) |
| | KANM800101 | Average relative probability of helix (Kanehisa-Tsong, 1980) |
| | KRIW790103 | Side chain volume (Krigbaum-Komoriya, 1979) |
| | KYTJ820101 | Hydropathy index (Kyte-Doolittle, 1982) |
| | MAXF760105 | Normalized frequency of zeta L (Maxfield-Scheraga, 1976) |
| | MAXF760106 | Normalized frequency of alpha region (Maxfield-Scheraga, 1976) |
| | MEEJ800101 | Retention coefficient in HPLC, pH7.4 (Meek, 1980) |
| | MEEJ810102 | Retention coefficient in NaH2PO4 (Meek-Rossetti, 1981) |
| | MEIH800102 | Average reduced distance for side chain (Meirovitch et al., 1980) |
| | NAGK730101 | Normalized frequency of alpha-helix (Nagano, 1973) |
| | NAGK730103 | Normalized frequency of coil (Nagano, 1973) |
| | NAKH900104 | Normalized composition of mt-proteins (Nakashima et al., 1990) |
| | NAKH900106 | Normalized composition from animal (Nakashima et al., 1990) |
| | NAKH900108 | Normalized composition from fungi and plant (Nakashima et al., 1990) |
| | NAKH900111 | Transmembrane regions of non-mt-proteins (Nakashima et al., 1990) |
| | NAKH920102 | AA composition of CYT2 of single-spanning proteins (Nakashima-Nishikawa, 1992) |
| | NAKH920104 | AA composition of EXT2 of single-spanning proteins (Nakashima-Nishikawa, 1992) |
| | NAKH920107 | AA composition of EXT of multi-spanning proteins (Nakashima-Nishikawa, 1992) |
| | NAKH920108 | AA composition of MEM of multi-spanning proteins (Nakashima-Nishikawa, 1992) |
| | OOBM770104 | Average non-bonded energy per residue (Oobatake-Ooi, 1977) |
| | OOBM850102 | Optimized propensity to form reverse turn (Oobatake et al., 1985) |
| | PONP800102 | Average gain in surrounding hydrophobicity (Ponnuswamy et al., 1980) |
| | PONP800103 | Average gain ratio in surrounding hydrophobicity (Ponnuswamy et al., 1980) |
| | PONP800106 | Surrounding hydrophobicity in turn (Ponnuswamy et al., 1980) |

| | |
|---|---|
| PRAM820103 | Correlation coefficient in regression analysis (Prabhakaran-Ponnuswamy, 1982) |
| QIAN880108 | Weights for alpha-helix at the window position of 1 (Qian-Sejnowski, 1988) |
| QIAN880119 | Weights for beta-sheet at the window position of -1 (Qian-Sejnowski, 1988) |
| QIAN880125 | Weights for beta-sheet at the window position of 5 (Qian-Sejnowski, 1988) |
| QIAN880132 | Weights for coil at the window position of -1 (Qian-Sejnowski, 1988) |
| RACS770102 | Average reduced distance for side chain (Rackovsky-Scheraga, 1977) |
| RACS820101 | Average relative fractional occurrence in A0(i) (Rackovsky-Scheraga, 1982) |
| RACS820111 | Average relative fractional occurrence in E0(i-1) (Rackovsky-Scheraga, 1982) |
| RADA880103 | Transfer free energy from vap to chx (Radzicka-Wolfenden, 1988) |
| RADA880105 | Transfer free energy from vap to oct (Radzicka-Wolfenden, 1988) |
| RADA880106 | Accessible surface area (Radzicka-Wolfenden, 1988) |
| RADA880107 | Energy transfer from out to in(95%buried) (Radzicka-Wolfenden, 1988) |
| RICJ880103 | Relative preference value at N-cap (Richardson-Richardson, 1988) |
| RICJ880111 | Relative preference value at C4 (Richardson-Richardson, 1988) |
| RICJ880114 | Relative preference value at C1 (Richardson-Richardson, 1988) |
| RICJ880116 | Relative preference value at C' (Richardson-Richardson, 1988) |
| ROBB760102 | Information measure for N-terminal helix (Robson-Suzuki, 1976) |
| ROBB760111 | Information measure for C-terminal turn (Robson-Suzuki, 1976) |
| ROSM880103 | Loss of Side chain hydropathy by helix formation (Roseman, 1988) |
| SNEP660103 | Principal component III (Sneath, 1966) |
| SNEP660104 | Principal component IV (Sneath, 1966) |
| SWER830101 | Optimal matching hydrophobicity (Sweet-Eisenberg, 1983) |
| TANS770103 | Normalized frequency of extended structure (Tanaka-Scheraga, 1977) |
| TANS770105 | Normalized frequency of chain reversal S (Tanaka-Scheraga, 1977) |
| TANS770109 | Normalized frequency of coil (Tanaka-Scheraga, 1977) |
| VASM830103 | Relative population of conformational state E (Vasquez et al., 1983) |
| WARP780101 | Average interactions per side chain atom (Warme-Morgan, 1978) |
| WEBA780101 | RF value in high salt chromatography (Weber-Lacey, 1978) |
| WERD780103 | Free energy change of alpha(Ri) to alpha(Rh) (Wertz-Scheraga, 1978) |
| WOLS870101 | Principal property value z1 (Wold et al., 1987) |
| WOLS870103 | Principal property value z3 (Wold et al., 1987) |
| YUTK870103 | Activation Gibbs energy of unfolding, pH7.0 (Yutani et al., 1987) |
| YUTK870104 | Activation Gibbs energy of unfolding, pH9.0 (Yutani et al., 1987) |
| ZASB820101 | Dependence of partition coefficient on ionic strength (Zaslavsky et al., 1982) |
| AURR980101 | Normalized positional residue frequency at helix termini N4'(Aurora-Rose, 1998) |
| AURR980104 | Normalized positional residue frequency at helix termini N'(Aurora-Rose, 1998) |
| AURR980106 | Normalized positional residue frequency at helix termini N1 (Aurora-Rose, 1998) |
| AURR980110 | Normalized positional residue frequency at helix termini N5 (Aurora-Rose, 1998) |
| VINM940102 | Normalized flexibility parameters (B-values) for each residue surrounded by none rigid neighbours (Vihinen et al., 1994) |
| MUNV940105 | Free energy in beta-strand region (Munoz-Serrano, 1994) |
| WIMW960101 | Free energies of transfer of AcWl-X-LL peptides from bilayer interface to water (Wimley-White, 1996) |
| MONM990101 | Turn propensity scale for transmembrane helices (Monne et al., 1999) |
| BLAM930101 | Alpha helix propensity of position 44 in T4 lysozyme (Blaber et al., 1993) |
| PARS000101 | p-Values of mesophilic proteins based on the distributions of B values (Parthasarathy-Murthy, 2000) |
| NADH010107 | Hydropathy scale based on self-information values in the two-state model (50% accessibility) (Naderi-Manesh et al., 2001) |

| | | |
|---|---|---|
| | MONM990201 | Averaged turn propensities in a transmembrane helix (Monne et al., 1999) |
| | CEDJ970104 | Composition of amino acids in intracellular proteins (percent) (Cedano et al., 1997) |
| | FUKS010108 | Interior composition of amino acids in nuclear proteins (percent) (Fukuchi-Nishikawa, 2001) |
| | MITS020101 | Amphiphilicity index (Mitaku et al., 2002) |
| | WILM950101 | Hydrophobicity coefficient in RP-HPLC, C18 with 0.1%TFA/MeCN/H2O (Wilce et al. 1995) |
| | WILM950102 | Hydrophobicity coefficient in RP-HPLC, C8 with 0.1%TFA/MeCN/H2O (Wilce et al. 1995) |
| | GUOD860101 | Retention coefficient at pH 2 (Guo et al., 1986) |
| | JURD980101 | Modified Kyte-Doolittle hydrophobicity scale (Juretic et al., 1998) |
| | BASU050102 | Interactivity scale obtained by maximizing the mean of correlation coefficient over single-domain globular proteins (Bastolla et al., 2005) |
| | GEOR030101 | Linker propensity from all dataset (George-Heringa, 2003) |
| | GEOR030104 | Linker propensity from 3-linker dataset (George-Heringa, 2003) |
| | GEOR030107 | Linker propensity from long dataset (linker length is greater than 14 residues) (George-Heringa, 2003) |
| | DIGM050101 | Hydrostatic pressure asymmetry index, PAI (Di Giulio, 2005) |
| | WOLR790101 | Hydrophobicity index (Wolfenden et al., 1979) |
| | OLSK800101 | Average internal preferences (Olsen, 1980) |
| | CORJ870102 | SWEIG index (Cornette et al., 1987) |
| | KARS160103 | Total weighted degree of the graph (obtained by adding all the weights of all the vertices) (Karkbara-Knisley, 2016) |
| | KARS160109 | Maximum eigenvalue of the weighted Laplacian matrix of the graph (Karkbara-Knisley, 2016) |
| | KARS160112 | Second smallest eigenvalue of the Laplacian matrix of the graph (Karkbara-Knisley, 2016) |
| ATRX | ANDN920101 | alpha-CH chemical shifts (Andersen et al., 1992) |
| | ARGP820102 | Signal sequence helical potential (Argos et al., 1982) |
| | ARGP820103 | Membrane-buried preference parameters (Argos et al., 1982) |
| | BULH740102 | Apparent partial specific volume (Bull-Breese, 1974) |
| | BUNA790101 | alpha-NH chemical shifts (Bundi-Wuthrich, 1979) |
| | BUNA790103 | Spin-spin coupling constants 3JHalpha-NH (Bundi-Wuthrich, 1979) |
| | CHAM820102 | Free energy of solution in water, kcal/mole (Charton-Charton, 1982) |
| | CHAM830103 | The number of atoms in the side chain labelled 1+1 (Charton-Charton, 1983) |
| | CHOC760102 | Residue accessible surface area in folded protein (Chothia, 1976) |
| | CHOC760103 | Proportion of residues 95% buried (Chothia, 1976) |
| | CHOP780202 | Normalized frequency of beta-sheet (Chou-Fasman, 1978b) |
| | CHOP780205 | Normalized frequency of C-terminal helix (Chou-Fasman, 1978b) |
| | CHOP780208 | Normalized frequency of N-terminal beta-sheet (Chou-Fasman, 1978b) |
| | CHOP780209 | Normalized frequency of C-terminal beta-sheet (Chou-Fasman, 1978b) |
| | CHOP780211 | Normalized frequency of C-terminal non beta region (Chou-Fasman, 1978b) |
| | CHOP780213 | Frequency of the 2nd residue in turn (Chou-Fasman, 1978b) |
| | CHOP780216 | Normalized frequency of the 2nd and 3rd residues in turn (Chou-Fasman, 1978b) |
| | COHE430101 | Partial specific volume (Cohn-Edsall, 1943) |
| | FAUJ880109 | Number of hydrogen bond donors (Fauchere et al., 1988) |
| | FAUJ880113 | pK-a(RCOOH) (Fauchere et al., 1988) |
| | FINA910104 | Helix termination parameter at posision j+1 (Finkelstein et al., 1991) |
| | GEIM800111 | Aperiodic indices for alpha/beta-proteins (Geisow-Roberts, 1980) |
| | GRAR740101 | Composition (Grantham, 1974) |
| | ISOY800107 | Normalized relative frequency of double bend (Isogai et al., 1980) |
| | JANJ790102 | Transfer free energy (Janin, 1979) |
| | KARP850101 | Flexibility parameter for no rigid neighbors (Karplus-Schulz, 1985) |
| | KLEP840101 | Net charge (Klein et al., 1984) |
| | LEVM760107 | van der Waals parameter epsilon (Levitt, 1976) |
| | LEVM780103 | Normalized frequency of reverse turn, with weights (Levitt, 1978) |

| | |
|---|---|
| LEVM780104 | Normalized frequency of alpha-helix, unweighted (Levitt, 1978) |
| LEVM780106 | Normalized frequency of reverse turn, unweighted (Levitt, 1978) |
| MAXF760104 | Normalized frequency of left-handed alpha-helix (Maxfield-Scheraga, 1976) |
| MAXF760106 | Normalized frequency of alpha region (Maxfield-Scheraga, 1976) |
| MCMT640101 | Refractivity (McMeekin et al., 1964), Cited by Jones (1975) |
| MEEJ800101 | Retention coefficient in HPLC, pH7.4 (Meek, 1980) |
| NAGK730101 | Normalized frequency of alpha-helix (Nagano, 1973) |
| NAKH900104 | Normalized composition of mt-proteins (Nakashima et al., 1990) |
| NAKH900106 | Normalized composition from animal (Nakashima et al., 1990) |
| NAKH900108 | Normalized composition from fungi and plant (Nakashima et al., 1990) |
| NAKH900111 | Transmembrane regions of non-mt-proteins (Nakashima et al., 1990) |
| NAKH920101 | AA composition of CYT of single-spanning proteins (Nakashima-Nishikawa, 1992) |
| NAKH920102 | AA composition of CYT2 of single-spanning proteins (Nakashima-Nishikawa, 1992) |
| OOBM850102 | Optimized propensity to form reverse turn (Oobatake et al., 1985) |
| OOBM850104 | Optimized average non-bonded energy per atom (Oobatake et al., 1985) |
| PALJ810114 | Normalized frequency of turn in all-beta class (Palau et al., 1981) |
| PALJ810116 | Normalized frequency of turn in alpha/beta class (Palau et al., 1981) |
| PRAM820101 | Intercept in regression analysis (Prabhakaran-Ponnuswamy, 1982) |
| PRAM900104 | Relative frequency in reverse-turn (Prabhakaran, 1990) |
| PTIO830101 | Helix-coil equilibrium constant (Ptitsyn-Finkelstein, 1983) |
| QIAN880101 | Weights for alpha-helix at the window position of -6 (Qian-Sejnowski, 1988) |
| QIAN880103 | Weights for alpha-helix at the window position of -4 (Qian-Sejnowski, 1988) |
| QIAN880109 | Weights for alpha-helix at the window position of 2 (Qian-Sejnowski, 1988) |
| QIAN880110 | Weights for alpha-helix at the window position of 3 (Qian-Sejnowski, 1988) |
| QIAN880111 | Weights for alpha-helix at the window position of 4 (Qian-Sejnowski, 1988) |
| QIAN880124 | Weights for beta-sheet at the window position of 4 (Qian-Sejnowski, 1988) |
| QIAN880125 | Weights for beta-sheet at the window position of 5 (Qian-Sejnowski, 1988) |
| QIAN880128 | Weights for coil at the window position of -5 (Qian-Sejnowski, 1988) |
| QIAN880133 | Weights for coil at the window position of 0 (Qian-Sejnowski, 1988) |
| QIAN880135 | Weights for coil at the window position of 2 (Qian-Sejnowski, 1988) |
| QIAN880136 | Weights for coil at the window position of 3 (Qian-Sejnowski, 1988) |
| QIAN880139 | Weights for coil at the window position of 6 (Qian-Sejnowski, 1988) |
| RACS820106 | Average relative fractional occurrence in ER(i) (Rackovsky-Scheraga, 1982) |
| RACS820110 | Average relative fractional occurrence in EL(i-1) (Rackovsky-Scheraga, 1982) |
| RADA880107 | Energy transfer from out to in(95%buried) (Radzicka-Wolfenden, 1988) |
| RICJ880105 | Relative preference value at N2 (Richardson-Richardson, 1988) |
| RICJ880109 | Relative preference value at Mid (Richardson-Richardson, 1988) |
| RICJ880110 | Relative preference value at C5 (Richardson-Richardson, 1988) |
| ROBB760104 | Information measure for C-terminal helix (Robson-Suzuki, 1976) |
| ROBB760106 | Information measure for pleated-sheet (Robson-Suzuki, 1976) |
| ROBB760107 | Information measure for extended without H-bond (Robson-Suzuki, 1976) |
| SUEM840101 | Zimm-Bragg parameter s at 20 C (Sueki et al., 1984) |
| TANS770102 | Normalized frequency of isolated helix (Tanaka-Scheraga, 1977) |
| TANS770104 | Normalized frequency of chain reversal R (Tanaka-Scheraga, 1977) |
| TANS770108 | Normalized frequency of zeta R (Tanaka-Scheraga, 1977) |
| WERD780104 | Free energy change of epsilon(i) to alpha(Rh) (Wertz-Scheraga, 1978) |
| YUTK870102 | Unfolding Gibbs energy in water, pH9.0 (Yutani et al., 1987) |
| ZASB820101 | Dependence of partition coefficient on ionic strength (Zaslavsky et al., |

| | | |
|---|---|---|
| | | 1982) |
| | ZIMJ680104 | Isoelectric point (Zimmerman et al., 1968) |
| | ZIMJ680105 | RF rank (Zimmerman et al., 1968) |
| | AURR980101 | Normalized positional residue frequency at helix termini N4'(Aurora-Rose, 1998) |
| | AURR980104 | Normalized positional residue frequency at helix termini N'(Aurora-Rose, 1998) |
| | AURR980106 | Normalized positional residue frequency at helix termini N1 (Aurora-Rose, 1998) |
| | AURR980107 | Normalized positional residue frequency at helix termini N2 (Aurora-Rose, 1998) |
| | AURR980109 | Normalized positional residue frequency at helix termini N4 (Aurora-Rose, 1998) |
| | AURR980118 | Normalized positional residue frequency at helix termini C" (Aurora-Rose, 1998) |
| | MUNV940101 | Free energy in alpha-helical conformation (Munoz-Serrano, 1994) |
| | KUMS000103 | Distribution of amino acid residues in the alpha-helices in thermophilic proteins (Kumar et al., 2000) |
| | FODM020101 | Propensity of amino acids within pi-helices (Fodje-Al-Karadaghi, 2002) |
| | NADH010101 | Hydropathy scale based on self-information values in the two-state model (5% accessibility) (Naderi-Manesh et al., 2001 |
| | NADH010106 | Hydropathy scale based on self-information values in the two-state model (36% accessibility) (Naderi-Manesh et al., 2001) |
| | KOEP990101 | Alpha-helix propensity derived from designed sequences (Koehl-Levitt, 1999) |
| | KOEP990102 | Beta-sheet propensity derived from designed sequences (Koehl-Levitt, 1999) |
| | FUKS010111 | Entire chain composition of amino acids in extracellular proteins of mesophiles (percent) (Fukuchi-Nishikawa, 2001) |
| | SUYM030101 | Linker propensity index (Suyama-Ohara, 2003) |
| | PUNT030102 | Knowledge-based membrane-propensity scale from 3D_Helix in MPtopo databases (Punta-Maritan, 2003) |
| | GEOR030105 | Linker propensity from small dataset (linker length is less than six residues) (George-Heringa, 2003) |
| | GEOR030109 | Linker propensity from non-helical (annotated by DSSP) dataset (George-Heringa, 2003) |
| | OLSK800101 | Average internal preferences (Olsen, 1980) |
| | GUYH850104 | Apparent partition energies calculated from Janin index (Guy, 1985) |
| | GUYH850105 | Apparent partition energies calculated from Chothia index (Guy, 1985) |
| | KARS160120 | Weighted minimum eigenvalue based on the atomic numbers (Karkbara-Knisley, 2016) |
| BCOR | ARGP820101 | Hydrophobicity index (Argos et al., 1982) |
| | ARGP820102 | Signal sequence helical potential (Argos et al., 1982) |
| | BIOV880101 | Information value for accessibility; average fraction 35% (Biou et al., 1988) |
| | CHAM830102 | A parameter defined from the residuals obtained from the best correlation of the Chou-Fasman parameter of beta-sheet (Charton-Charton, 1983) |
| | GEIM800107 | Beta-strand indices for alpha/beta-proteins (Geisow-Roberts, 1980) |
| | JANJ790101 | Ratio of buried and accessible molar fractions (Janin, 1979) |
| | JOND750101 | Hydrophobicity (Jones, 1975) |
| | LEVM780102 | Normalized frequency of beta-sheet, with weights (Levitt, 1978) |
| | MAXF760103 | Normalized frequency of zeta R (Maxfield-Scheraga, 1976) |
| | NAKH900102 | SD of AA composition of total proteins (Nakashima et al., 1990) |
| | NAKH920102 | AA composition of CYT2 of single-spanning proteins (Nakashima-Nishikawa, 1992) |
| | NAKH920108 | AA composition of MEM of multi-spanning proteins (Nakashima-Nishikawa, 1992) |
| | OOBM770103 | Long range non-bonded energy per atom (Oobatake-Ooi, 1977) |
| | PALJ810103 | Normalized frequency of beta-sheet from LG (Palau et al., 1981) |
| | PALJ810111 | Normalized frequency of beta-sheet in alpha+beta class (Palau et al., |

| | | |
|---|---|---|
| | | 1981) |
| | PRAM900103 | Relative frequency in beta-sheet (Prabhakaran, 1990) |
| | QIAN880110 | Weights for alpha-helix at the window position of 3 (Qian-Sejnowski, 1988) |
| | QIAN880129 | Weights for coil at the window position of -4 (Qian-Sejnowski, 1988) |
| | RICJ880117 | Relative preference value at C" (Richardson-Richardson, 1988) |
| | ROBB760106 | Information measure for pleated-sheet (Robson-Suzuki, 1976) |
| | ROBB790101 | Hydration free energy (Robson-Osguthorpe, 1979) |
| | ZASB820101 | Dependence of partition coefficient on ionic strength (Zaslavsky et al., 1982) |
| | MUNV940103 | Free energy in beta-strand conformation (Munoz-Serrano, 1994) |
| | FUKS010103 | Surface composition of amino acids in extracellular proteins of mesophiles (percent) (Fukuchi-Nishikawa, 2001) |
| | WILM950103 | Hydrophobicity coefficient in RP-HPLC, C4 with 0.1%TFA/MeCN/H2O (Wilce et al. 1995) |
| | JURD980101 | Modified Kyte-Doolittle hydrophobicity scale (Juretic et al., 1998) |
| | GUYH850102 | Apparent partition energies calculated from Wertz-Scheraga index (Guy, 1985) |
| CREBBP | FAUJ880112 | Negative charge (Fauchere et al., 1988) |
| | HOPA770101 | Hydration number (Hopfinger, 1971), Cited by Charton-Charton (1982) |
| | JOND750102 | pK (-COOH) (Jones, 1975) |
| | KARP850102 | Flexibility parameter for one rigid neighbor (Karplus-Schulz, 1985) |
| | MAXF760104 | Normalized frequency of left-handed alpha-helix (Maxfield-Scheraga, 1976) |
| | MAXF760105 | Normalized frequency of zeta L (Maxfield-Scheraga, 1976) |
| | NAKH920104 | AA composition of EXT2 of single-spanning proteins (Nakashima-Nishikawa, 1992) |
| | QIAN880123 | Weights for beta-sheet at the window position of 3 (Qian-Sejnowski, 1988) |
| | QIAN880124 | Weights for beta-sheet at the window position of 4 (Qian-Sejnowski, 1988) |
| | RACS820106 | Average relative fractional occurrence in ER(i) (Rackovsky-Scheraga, 1982) |
| | RICJ880112 | Relative preference value at C3 (Richardson-Richardson, 1988) |
| | TANS770109 | Normalized frequency of coil (Tanaka-Scheraga, 1977) |
| | ZIMJ680103 | Polarity (Zimmerman et al., 1968) |
| | AURR980113 | Normalized positional residue frequency at helix termini C3 (Aurora-Rose, 1998) |
| | AURR980115 | Normalized positional residue frequency at helix termini C1 (Aurora-Rose, 1998) |
| | AURR980117 | Normalized positional residue frequency at helix termini C' (Aurora-Rose, 1998) |
| | KUMS000104 | Distribution of amino acid residues in the alpha-helices in mesophilic proteins (Kumar et al., 2000) |
| | FUKS010101 | Surface composition of amino acids in intracellular proteins of thermophiles (percent) (Fukuchi-Nishikawa, 2001) |
| | KARS160113 | Weighted domination number using the atomic number (Karkbara-Knisley, 2016) |
| | KARS160114 | Average weighted eccentricity based on the the atomic number (Karkbara-Knisley, 2016) |
| | KARS160116 | Weighted diameter based on the atomic number (maximum eccentricity) (Karkbara-Knisley, 2016) |
| DNMT3A | FAUJ880111 | Positive charge (Fauchere et al., 1988) |
| | LAWE840101 | Transfer free energy, CHP/water (Lawson et al., 1984) |
| | QIAN880110 | Weights for alpha-helix at the window position of 3 (Qian-Sejnowski, 1988) |
| | RICJ880105 | Relative preference value at N2 (Richardson-Richardson, 1988) |
| | AURR980120 | Normalized positional residue frequency at helix termini C4' (Aurora-Rose, 1998) |
| | FUKS010110 | Entire chain composition of amino acids in intracellular proteins of |

| | | |
|---|---|---|
| | | mesophiles (percent) (Fukuchi-Nishikawa, 2001) |
| | WILM950104 | Hydrophobicity coefficient in RP-HPLC, C18 with 0.1%TFA/2-PrOH/MeCN/H2O (Wilce et al. 1995) |
| | KARS160120 | Weighted minimum eigenvalue based on the atomic numbers (Karkbara-Knisley, 2016) |
| EP300 | BEGF750102 | Conformational parameter of beta-structure (Beghin-Dirkx, 1975) |
| | CHAM830103 | The number of atoms in the side chain labelled 1+1 (Charton-Charton, 1983) |
| | DAYM780201 | Relative mutability (Dayhoff et al., 1978b) |
| | FAUJ880112 | Negative charge (Fauchere et al., 1988) |
| | GEIM800105 | Beta-strand indices (Geisow-Roberts, 1980) |
| | JANJ790101 | Ratio of buried and accessible molar fractions (Janin, 1979) |
| | PALJ810115 | Normalized frequency of turn in alpha+beta class (Palau et al., 1981) |
| | SNEP660103 | Principal component III (Sneath, 1966) |
| | NADH010106 | Hydropathy scale based on self-information values in the two-state model (36% accessibility) (Naderi-Manesh et al., 2001) |
| | NADH010107 | Hydropathy scale based on self-information values in the two-state model (50% accessibility) (Naderi-Manesh et al., 2001) |
| | WILM950102 | Hydrophobicity coefficient in RP-HPLC, C8 with 0.1%TFA/MeCN/H2O (Wilce et al. 1995) |
| | WILM950104 | Hydrophobicity coefficient in RP-HPLC, C18 with 0.1%TFA/2-PrOH/MeCN/H2O (Wilce et al. 1995) |
| | GEOR030104 | Linker propensity from 3-linker dataset (George-Heringa, 2003) |
| | GEOR030105 | Linker propensity from small dataset (linker length is less than six residues) (George-Heringa, 2003) |
| | OLSK800101 | Average internal preferences (Olsen, 1980) |
| | KARS160109 | Maximum eigenvalue of the weighted Laplacian matrix of the graph (Karkbara-Knisley, 2016) |
| EZH2 | GOLD730101 | Hydrophobicity factor (Goldsack-Chalifoux, 1973) |
| | RACS820114 | Value of theta(i-1) (Rackovsky-Scheraga, 1982) |
| | TANS770105 | Normalized frequency of chain reversal S (Tanaka-Scheraga, 1977) |
| | MUNV940105 | Free energy in beta-strand region (Munoz-Serrano, 1994) |
| | KARS160116 | Weighted diameter based on the atomic number (maximum eccentricity) (Karkbara-Knisley, 2016) |
| JAK2 | FASG760102 | Melting point (Fasman, 1976) |
| | FAUJ880109 | Number of hydrogen bond donors (Fauchere et al., 1988) |
| | FAUJ880111 | Positive charge (Fauchere et al., 1988) |
| | GEIM800104 | Alpha-helix indices for alpha/beta-proteins (Geisow-Roberts, 1980) |
| | GRAR740101 | Composition (Grantham, 1974) |
| | KRIW790102 | Fraction of site occupied by water (Krigbaum-Komoriya, 1979) |
| | LEVM760106 | van der Waals parameter R0 (Levitt, 1976) |
| | LEWP710101 | Frequency of occurrence in beta-bends (Lewis et al., 1971) |
| | MEEJ800102 | Retention coefficient in HPLC, pH2.1 (Meek, 1980) |
| | NAKH900104 | Normalized composition of mt-proteins (Nakashima et al., 199 |
| | NAKH900106 | Normalized composition from animal (Nakashima et al., 1990) |
| | OOBM850101 | Optimized beta-structure-coil equilibrium constant (Oobatake et al., 1985) |
| | PALJ810114 | Normalized frequency of turn in all-beta class (Palau et al., 1981) |
| | PONP800105 | Surrounding hydrophobicity in beta-sheet (Ponnuswamy et al., 1980) |
| | PONP800107 | Accessibility reduction ratio (Ponnuswamy et al., 1980) |
| | PTIO830102 | Beta-coil equilibrium constant (Ptitsyn-Finkelstein, 1983) |
| | QIAN880115 | Weights for beta-sheet at the window position of -5 (Qian-Sejnowski, 1988) |
| | QIAN880133 | Weights for coil at the window position of 0 (Qian-Sejnowski, 1988) |
| | QIAN880137 | Weights for coil at the window position of 4 (Qian-Sejnowski, 1988) |
| | RICJ880107 | Relative preference value at N4 (Richardson-Richardson, 1988) |
| | ROBB790101 | Hydration free energy (Robson-Osguthorpe, 1979) |
| | ROSG850102 | Mean fractional area loss (Rose et al., 1985) |
| | TANS770106 | Normalized frequency of chain reversal D (Tanaka-Scheraga, 1977) |
| | TANS770110 | Normalized frequency of chain reversal (Tanaka-Scheraga, 1977) |
| | VENT840101 | Bitterness (Venanzi, 1984) |

| | | |
|---|---|---|
| | WOLR810101 | Hydration potential (Wolfenden et al., 1981) |
| | ZIMJ680105 | RF rank (Zimmerman et al., 1968) |
| | AURR980102 | Normalized positional residue frequency at helix termini N"' (Aurora-Rose, 1998) |
| | AURR980106 | Normalized positional residue frequency at helix termini N1 (Aurora-Rose, 1998) |
| | AURR980118 | Normalized positional residue frequency at helix termini C" (Aurora-Rose, 1998) |
| | NADH010102 | Hydropathy scale based on self-information values in the two-state model (9% accessibility) (Naderi-Manesh et al., 2001) |
| | FUKS010108 | Interior composition of amino acids in nuclear proteins (percent) (Fukuchi-Nishikawa, 2001) |
| | MITS020101 | Amphiphilicity index (Mitaku et al., 2002) |
| | WOLR790101 | Hydrophobicity index (Wolfenden et al., 1979) |
| | ENGD860101 | Hydrophobicity index (Engelman et al., 1986) |
| | KARS160120 | Weighted minimum eigenvalue based on the atomic numbers (Karkbara-Knisley, 2016) |
| KMT2A | BEGF750103 | Conformational parameter of beta-turn (Beghin-Dirkx, 1975) |
| | GARJ730101 | Partition coefficient (Garel et al., 1973) |
| | ISOY800105 | Normalized relative frequency of bend S (Isogai et al., 1980) |
| | JANJ790101 | Ratio of buried and accessible molar fractions (Janin, 1979) |
| | KANM800102 | Average relative probability of beta-sheet (Kanehisa-Tsong, 1980) |
| | KARP850103 | Flexibility parameter for two rigid neighbors (Karplus-Schulz, 1985) |
| | LEVM760103 | Side chain angle theta(AAR) (Levitt, 1976) |
| | NAKH900113 | Ratio of average and computed composition (Nakashima et al., 1990) |
| | QIAN880131 | Weights for coil at the window position of -2 (Qian-Sejnowski, 1988) |
| | QIAN880138 | Weights for coil at the window position of 5 (Qian-Sejnowski, 1988) |
| | ZIMJ680102 | Bulkiness (Zimmerman et al., 1968) |
| | PARS000102 | p-Values of thermophilic proteins based on the distributions of B values (Parthasarathy-Murthy, 2000) |
| | ZHOH040102 | The relative stability scale extracted from mutation experiments (Zhou-Zhou, 2004) |
| | DIGM050101 | Hydrostatic pressure asymmetry index, PAI (Di Giulio, 2005) |
| | KARS160110 | Minimum eigenvalue of the weighted Laplacian matrix of the graph (Karkbara-Knisley, 2016) |
| KMT2C | CHOC760103 | Proportion of residues 95% buried (Chothia, 1976) |
| | CHOC760104 | Proportion of residues 100% buried (Chothia, 1976) |
| | CIDH920104 | Normalized hydrophobicity scales for alpha/beta-proteins (Cid et al., 1992) |
| | CIDH920105 | Normalized average hydrophobicity scales (Cid et al., 1992) |
| | DESM900102 | Average membrane preference: AMP07 (Degli Esposti et al., 1990) |
| | GARJ730101 | Partition coefficient (Garel et al., 1973) |
| | GRAR740103 | Volume (Grantham, 1974) |
| | JANJ780102 | Percentage of buried residues (Janin et al., 1978) |
| | KLEP840101 | Net charge (Klein et al., 1984) |
| | LEVM760103 | Side chain angle theta(AAR) (Levitt, 1976) |
| | MANP780101 | Average surrounding hydrophobicity (Manavalan-Ponnuswamy, 1978) |
| | MAXF760104 | Normalized frequency of left-handed alpha-helix (Maxfield-Scheraga, 1976) |
| | MAXF760105 | Normalized frequency of zeta L (Maxfield-Scheraga, 1976) |
| | MEEJ800101 | Retention coefficient in HPLC, pH7.4 (Meek, 1980) |
| | MEIH800103 | Average side chain orientation angle (Meirovitch et al., 1980) |
| | NAKH920102 | AA composition of CYT2 of single-spanning proteins (Nakashima-Nishikawa, 1992) |
| | NISK800101 | 8 A contact number (Nishikawa-Ooi, 1980) |
| | NOZY710101 | Transfer energy, organic solvent/water (Nozaki-Tanford, 1971) |
| | PALJ810116 | Normalized frequency of turn in alpha/beta class (Palau et al., 1981) |
| | PONP800103 | Average gain ratio in surrounding hydrophobicity (Ponnuswamy et al., 1980) |
| | PTIO830102 | Beta-coil equilibrium constant (Ptitsyn-Finkelstein, 1983) |

| | | |
|---|---|---|
| | QIAN880118 | Weights for beta-sheet at the window position of -2 (Qian-Sejnowski, 1988) |
| | QIAN880122 | Weights for beta-sheet at the window position of 2 (Qian-Sejnowski, 1988) |
| | QIAN880124 | Weights for beta-sheet at the window position of 4 (Qian-Sejnowski, 1988) |
| | RACS770101 | Average reduced distance for C-alpha (Rackovsky-Scheraga, 1977) |
| | RACS770103 | Side chain orientational preference (Rackovsky-Scheraga, 1977) |
| | RACS820109 | Average relative fractional occurrence in AL(i-1) (Rackovsky-Scheraga, 1982) |
| | RICJ880115 | Relative preference value at C-cap (Richardson-Richardson, 1988) |
| | ROSG850102 | Mean fractional area loss (Rose et al., 1985) |
| | VASM830102 | Relative population of conformational state C (Vasquez et al., 1983) |
| | WERD780101 | Propensity to be buried inside (Wertz-Scheraga, 1978) |
| | WOLS870103 | Principal property value z3 (Wold et al., 1987) |
| | ZIMJ680104 | Isoelectric point (Zimmerman et al., 1968) |
| | AURR980103 | Normalized positional residue frequency at helix termini N" (Aurora-Rose, 1998) |
| | VINM940103 | Normalized flexibility parameters (B-values) for each residue surrounded by one rigid neighbours (Vihinen et al., 1994) |
| | PARS000101 | p-Values of mesophilic proteins based on the distributions of B values (Parthasarathy-Murthy, 2000) |
| | NADH010101 | Hydropathy scale based on self-information values in the two-state model (5% accessibility) (Naderi-Manesh et al., 2001) |
| | NADH010102 | Hydropathy scale based on self-information values in the two-state model (9% accessibility) (Naderi-Manesh et al., 2001) |
| | NADH010103 | Hydropathy scale based on self-information values in the two-state model (16% accessibility) (Naderi-Manesh et al., 2001) |
| | MITS020101 | Amphiphilicity index (Mitaku et al., 2002) |
| | WILM950104 | Hydrophobicity coefficient in RP-HPLC, C18 with 0.1%TFA/2-PrOH/MeCN/H2O (Wilce et al. 1995) |
| | GUOD860101 | Retention coefficient at pH 2 (Guo et al., 1986) |
| | BASU050103 | Interactivity scale obtained by maximizing the mean of correlation coefficient over pairs of sequences sharing the TIM barrel fold (Bastolla et al., 2005) |
| | GEOR030105 | Linker propensity from small dataset (linker length is less than six residues) (George-Heringa, 2003) |
| | CASG920101 | Hydrophobicity scale from native protein structures (Casari-Sippl, 19 |
| | CORJ870101 | NNEIG index (Cornette et al., 1987) |
| | MIYS990103 | Optimized relative partition energies - method B (Miyazawa-Jernigan, 1999) |
| KMT2D | BULH740102 | Apparent partial specific volume (Bull-Breese, 1974) |
| | BURA740101 | Normalized frequency of alpha-helix (Burgess et al., 1974) |
| | CHAM830105 | The number of atoms in the side chain labelled 3+1 (Charton-Charton, 1983) |
| | CHAM830106 | The number of bonds in the longest chain (Charton-Charton, 1983) |
| | CHAM830108 | A parameter of charge transfer donor capability (Charton-Charton, 1983) |
| | CHOC760101 | Residue accessible surface area in tripeptide (Chothia, 1976) |
| | CHOP780206 | Normalized frequency of N-terminal non helical region (Chou-Fasman, 1978b) |
| | CIDH920101 | Normalized hydrophobicity scales for alpha-proteins (Cid et al., 1992) |
| | FAUJ880108 | Localized electrical effect (Fauchere et al., 1988) |
| | FAUJ880112 | Negative charge (Fauchere et al., 1988) |
| | GARJ730101 | Partition coefficient (Garel et al., 1973) |
| | HOPT810101 | Hydrophilicity value (Hopp-Woods, 1981) |
| | ISOY800101 | Normalized relative frequency of alpha-helix (Isogai et al., 1980) |
| | ISOY800102 | Normalized relative frequency of extended structure (Isogai et al., 1980) |
| | ISOY800107 | Normalized relative frequency of double bend (Isogai et al., 1980) |
| | JOND750102 | pK (-COOH) (Jones, 1975) |
| | KANM800103 | Average relative probability of inner helix (Kanehisa-Tsong, 1980) |

| | |
|---|---|
| LEVM760104 | Side chain torsion angle phi(AAAR) (Levitt, 1976) |
| LEVM760107 | van der Waals parameter epsilon (Levitt, 1976) |
| NAKH900104 | Normalized composition of mt-proteins (Nakashima et al., 1990) |
| NAKH900106 | Normalized composition from animal (Nakashima et al., 1990) |
| NAKH920102 | AA composition of CYT2 of single-spanning proteins (Nakashima-Nishikawa, 1992) |
| NAKH920103 | AA composition of EXT of single-spanning proteins (Nakashima-Nishikawa, 1992) |
| OOBM850103 | Optimized transfer energy parameter (Oobatake et al., 1985) |
| OOBM850104 | Optimized average non-bonded energy per atom (Oobatake et al., 1985) |
| PALJ810105 | Normalized frequency of turn from LG (Palau et al., 1981) |
| PONP800101 | Surrounding hydrophobicity in folded form (Ponnuswamy et al., 1980) |
| PRAM820102 | Slope in regression analysis x 1.0E1 (Prabhakaran-Ponnuswamy, 1982) |
| QIAN880106 | Weights for alpha-helix at the window position of -1 (Qian-Sejnowski, 1988) |
| QIAN880114 | Weights for beta-sheet at the window position of -6 (Qian-Sejnowski, 1988) |
| QIAN880115 | Weights for beta-sheet at the window position of -5 (Qian-Sejnowski, 1988) |
| QIAN880128 | Weights for coil at the window position of -5 (Qian-Sejnowski, 1988) |
| QIAN880136 | Weights for coil at the window position of 3 (Qian-Sejnowski, 1988) |
| QIAN880139 | Weights for coil at the window position of 6 (Qian-Sejnowski, 1988) |
| RACS770103 | Side chain orientational preference (Rackovsky-Scheraga, 1977) |
| RACS820101 | Average relative fractional occurrence in A0(i) (Rackovsky-Scheraga, 1982) |
| RACS820106 | Average relative fractional occurrence in ER(i) (Rackovsky-Scheraga, 1982) |
| RACS820109 | Average relative fractional occurrence in AL(i-1) (Rackovsky-Scheraga, 1982) |
| RADA880103 | Transfer free energy from vap to chx (Radzicka-Wolfenden, 1988) |
| RADA880106 | Accessible surface area (Radzicka-Wolfenden, 1988) |
| RICJ880117 | Relative preference value at C" (Richardson-Richardson, 1988) |
| SIMZ760101 | Transfer free energy (Simon, 1976), Cited by Charton-Charton (1982) |
| SNEP660103 | Principal component III (Sneath, 1966) |
| VHEG790101 | Transfer free energy to lipophilic phase (von Heijne-Blomberg, 1979) |
| WARP780101 | Average interactions per side chain atom (Warme-Morgan, 1978) |
| YUTK870102 | Unfolding Gibbs energy in water, pH9.0 (Yutani et al., 1987) |
| YUTK870103 | Activation Gibbs energy of unfolding, pH7.0 (Yutani et al., 1987) |
| YUTK870104 | Activation Gibbs energy of unfolding, pH9.0 (Yutani et al., 1987) |
| AURR980108 | Normalized positional residue frequency at helix termini N3 (Aurora-Rose, 1998) |
| AURR980116 | Normalized positional residue frequency at helix termini Cc (Aurora-Rose, 1998) |
| WIMW960101 | Free energies of transfer of AcWl-X-LL peptides from bilayer interface to water (Wimley-White, 1996) |
| TAKK010101 | Side-chain contribution to protein stability (kJ/mol) (Takano-Yutani, 2001) |
| NADH010101 | Hydropathy scale based on self-information values in the two-state model (5% accessibility) (Naderi-Manesh et al., 2001) |
| NADH010104 | Hydropathy scale based on self-information values in the two-state model (20% accessibility) (Naderi-Manesh et al., 2001) |
| NADH010105 | Hydropathy scale based on self-information values in the two-state model (25% accessibility) (Naderi-Manesh et al., 2001) |
| NADH010106 | Hydropathy scale based on self-information values in the two-state model (36% accessibility) (Naderi-Manesh et al., 2001) |
| FUKS010101 | Surface composition of amino acids in intracellular proteins of thermophiles (percent) (Fukuchi-Nishikawa, 2001) |
| FUKS010104 | Surface composition of amino acids in nuclear proteins (percent) (Fukuchi-Nishikawa, 2001) |
| WILM950102 | Hydrophobicity coefficient in RP-HPLC, C8 with 0.1%TFA/MeCN/H2O |

| | | |
|---|---|---|
| | | (Wilce et al. 1995) |
| | KUHL950101 | Hydrophilicity scale (Kuhn et al., 1995) |
| | GEOR030101 | Linker propensity from all dataset (George-Heringa, 2003) |
| | GEOR030105 | Linker propensity from small dataset (linker length is less than six residues) (George-Heringa, 2003) |
| | PONJ960101 | Average volumes of residues (Pontius et al., 1996) |
| | WOLR790101 | Hydrophobicity index (Wolfenden et al., 1979) |
| | JACR890101 | Weights from the IFH scale (Jacobs-White, 1989) |
| | KARS160107 | Diameter (maximum eccentricity) (Karkbara-Knisley, 2016) |
| | KARS160112 | Second smallest eigenvalue of the Laplacian matrix of the graph (Karkbara-Knisley, 2016) |
| | KARS160120 | Weighted minimum eigenvalue based on the atomic numbers (Karkbara-Knisley, 2016) |
| NSD1 | ARGP820101 | Hydrophobicity index (Argos et al., 1982) |
| | ARGP820102 | Signal sequence helical potential (Argos et al., 1982) |
| | ARGP820103 | Membrane-buried preference parameters (Argos et al., 1982) |
| | BEGF750103 | Conformational parameter of beta-turn (Beghin-Dirkx, 1975) |
| | BULH740102 | Apparent partial specific volume (Bull-Breese, 1974) |
| | BUNA790101 | alpha-NH chemical shifts (Bundi-Wuthrich, 1979) |
| | CHAM830102 | A parameter defined from the residuals obtained from the best correlation of the Chou-Fasman parameter of beta-sheet (Charton-Charton, 1983) |
| | CHAM830105 | The number of atoms in the side chain labelled 3+1 (Charton-Charton, 1983) |
| | CHOP780208 | Normalized frequency of N-terminal beta-sheet (Chou-Fasman, 1978b) |
| | CHOP780214 | Frequency of the 3rd residue in turn (Chou-Fasman, 1978b) |
| | CIDH920103 | Normalized hydrophobicity scales for alpha+beta-proteins (Cid et al., 1992) |
| | CIDH920104 | Normalized hydrophobicity scales for alpha/beta-proteins (Cid et al., 1992) |
| | FAUJ830101 | Hydrophobic parameter pi (Fauchere-Pliska, 1983) |
| | FAUJ880110 | Number of full nonbonding orbitals (Fauchere et al., 1988) |
| | FAUJ880112 | Negative charge (Fauchere et al., 1988) |
| | FAUJ880113 | pK-a(RCOOH) (Fauchere et al., 1988) |
| | FINA910102 | Helix initiation parameter at posision i,i+1,i+2 (Finkelstein et al., 1991) |
| | GARJ730101 | Partition coefficient (Garel et al., 1973) |
| | GRAR740101 | Composition (Grantham, 1974) |
| | HOPA770101 | Hydration number (Hopfinger, 1971), Cited by Charton-Charton (1982) |
| | HUTJ700102 | Absolute entropy (Hutchens, 1970) |
| | ISOY800105 | Normalized relative frequency of bend S (Isogai et al., 1980) |
| | ISOY800108 | Normalized relative frequency of coil (Isogai et al., 1980) |
| | JANJ790101 | Ratio of buried and accessible molar fractions (Janin, 1979) |
| | JOND750101 | Hydrophobicity (Jones, 1975) |
| | KRIW710101 | Side chain interaction parameter (Krigbaum-Rubin, 1971) |
| | KRIW790101 | Side chain interaction parameter (Krigbaum-Komoriya, 1979) |
| | LAWE840101 | Transfer free energy, CHP/water (Lawson et al., 1984) |
| | LEVM760106 | van der Waals parameter R0 (Levitt, 1976) |
| | MEEJ800102 | Retention coefficient in HPLC, pH2.1 (Meek, 1980) |
| | NAGK730102 | Normalized frequency of bata-structure (Nagano, 1973) |
| | NAKH900103 | AA composition of mt-proteins (Nakashima et al., 1990) |
| | NAKH900107 | AA composition of mt-proteins from fungi and plant (Nakashima et al., 1990) |
| | NAKH900109 | AA composition of membrane proteins (Nakashima et al., 1990) |
| | PALJ810108 | Normalized frequency of alpha-helix in alpha+beta class (Palau et al., 1981) |
| | PARJ860101 | HPLC parameter (Parker et al., 1986) |
| | PLIV810101 | Partition coefficient (Pliska et al., 1981) |
| | PRAM820101 | Intercept in regression analysis (Prabhakaran-Ponnuswamy, 1982) |
| | PTIO830101 | Helix-coil equilibrium constant (Ptitsyn-Finkelstein, 1983) |
| | QIAN880102 | Weights for alpha-helix at the window position of -5 (Qian-Sejnowski, 1988) |

| | | |
|---|---|---|
| | QIAN880105 | Weights for alpha-helix at the window position of -2 (Qian-Sejnowski, 1988) |
| | QIAN880108 | Weights for alpha-helix at the window position of 1 (Qian-Sejnowski, 1988) |
| | QIAN880117 | Weights for beta-sheet at the window position of -3 (Qian-Sejnowski, 1988) |
| | QIAN880119 | Weights for beta-sheet at the window position of -1 (Qian-Sejnowski, 1988) |
| | QIAN880125 | Weights for beta-sheet at the window position of 5 (Qian-Sejnowski, 1988) |
| | RACS820102 | Average relative fractional occurrence in AR(i) (Rackovsky-Scheraga, 1982) |
| | RACS820104 | Average relative fractional occurrence in EL(i) (Rackovsky-Scheraga, 1982) |
| | RADA880102 | Transfer free energy from oct to wat (Radzicka-Wolfenden, 1988) |
| | RADA880107 | Energy transfer from out to in(95%buried) (Radzicka-Wolfenden, 1988) |
| | RICJ880104 | Relative preference value at N1 (Richardson-Richardson, 1988) |
| | RICJ880105 | Relative preference value at N2 (Richardson-Richardson, 1988) |
| | RICJ880107 | Relative preference value at N4 (Richardson-Richardson, 1988) |
| | RICJ880113 | Relative preference value at C2 (Richardson-Richardson, 1988) |
| | RICJ880117 | Relative preference value at C" (Richardson-Richardson, 1988) |
| | SUEM840102 | Zimm-Bragg parameter sigma x 1.0E4 (Sueki et al., 1984) |
| | TANS770109 | Normalized frequency of coil (Tanaka-Scheraga, 1977) |
| | YUTK870103 | Activation Gibbs energy of unfolding, pH7.0 (Yutani et al., 1987) |
| | YUTK870104 | Activation Gibbs energy of unfolding, pH9.0 (Yutani et al., 1987) |
| | ZIMJ680105 | RF rank (Zimmerman et al., 1968) |
| | AURR980102 | Normalized positional residue frequency at helix termini N"' (Aurora-Rose, 1998) |
| | AURR980106 | Normalized positional residue frequency at helix termini N1 (Aurora-Rose, 1998) |
| | AURR980107 | Normalized positional residue frequency at helix termini N2 (Aurora-Rose, 1998) |
| | AURR980113 | Normalized positional residue frequency at helix termini C3 (Aurora-Rose, 1998) |
| | AURR980119 | Normalized positional residue frequency at helix termini C"' (Aurora-Rose, 1998) |
| | MUNV940101 | Free energy in alpha-helical conformation (Munoz-Serrano, 1994) |
| | MUNV940102 | Free energy in alpha-helical region (Munoz-Serrano, 1994) |
| | MUNV940105 | Free energy in beta-strand region (Munoz-Serrano, 1994) |
| | KUMS000103 | Distribution of amino acid residues in the alpha-helices in thermophilic proteins (Kumar et al., 2000) |
| | KUMS000104 | Distribution of amino acid residues in the alpha-helices in mesophilic proteins (Kumar et al., 2000) |
| | FUKS010109 | Entire chain composition of amino acids in intracellular proteins of thermophiles (percent) (Fukuchi-Nishikawa, 2001) |
| | WILM950102 | Hydrophobicity coefficient in RP-HPLC, C8 with 0.1%TFA/MeCN/H2O (Wilce et al. 1995) |
| | GEOR030105 | Linker propensity from small dataset (linker length is less than six residues) (George-Heringa, 2003) |
| | GEOR030109 | Linker propensity from non-helical (annotated by DSSP) dataset (George-Heringa, 2003) |
| | DIGM050101 | Hydrostatic pressure asymmetry index, PAI (Di Giulio, 2005) |
| | JACR890101 | Weights from the IFH scale (Jacobs-White, 1989) |
| | COWR900101 | Hydrophobicity index, 3.0 pH (Cowan-Whittaker, 1990) |
| | BLAS910101 | Scaled side chain hydrophobicity values (Black-Mould, 1991) |
| | KARS160106 | Radius (minimum eccentricity) (Karkbara-Knisley, 2016) |
| | KARS160113 | Weighted domination number using the atomic number (Karkbara-Knisley, 2016) |
| | KARS160116 | Weighted diameter based on the atomic number (maximum eccentricity) (Karkbara-Knisley, 2016) |

| | | |
|---|---|---|
| | KARS160120 | Weighted minimum eigenvalue based on the atomic numbers (Karkbara-Knisley, 2016) |
| SETD2 | BURA740102 | Normalized frequency of extended structure (Burgess et al., 1974) |
| | CHAM830105 | The number of atoms in the side chain labelled 3+1 (Charton-Charton, 1983) |
| | CHAM830108 | A parameter of charge transfer donor capability (Charton-Charton, 1983) |
| | FAUJ880108 | Localized electrical effect (Fauchere et al., 1988) |
| | FINA910101 | Helix initiation parameter at posision i-1 (Finkelstein et al., 1991) |
| | FINA910103 | Helix termination parameter at posision j-2,j-1,j (Finkelstein et al., 1991) |
| | GEIM800105 | Beta-strand indices (Geisow-Roberts, 1980) |
| | GRAR740101 | Composition (Grantham, 1974) |
| | ISOY800106 | Normalized relative frequency of helix end (Isogai et al., 1980) |
| | JUKT750101 | Amino acid distribution (Jukes et al., 1975) |
| | KARP850103 | Flexibility parameter for two rigid neighbors (Karplus-Schulz, 1985) |
| | MAXF760103 | Normalized frequency of zeta R (Maxfield-Scheraga, 1976) |
| | MAXF760104 | Normalized frequency of left-handed alpha-helix (Maxfield-Scheraga, 1976) |
| | MAXF760105 | Normalized frequency of zeta L (Maxfield-Scheraga, 1976) |
| | MAXF760106 | Normalized frequency of alpha region (Maxfield-Scheraga, 1976) |
| | NAKH900107 | AA composition of mt-proteins from fungi and plant (Nakashima et al., 1990) |
| | NAKH920102 | AA composition of CYT2 of single-spanning proteins (Nakashima-Nishikawa, 1992) |
| | NAKH920105 | AA composition of MEM of single-spanning proteins (Nakashima-Nishikawa, 1992) |
| | NAKH920107 | AA composition of EXT of multi-spanning proteins (Nakashima-Nishikawa, 1992) |
| | OOBM850103 | Optimized transfer energy parameter (Oobatake et al., 1985) |
| | PALJ810104 | Normalized frequency of beta-sheet from CF (Palau et al., 1981) |
| | PALJ810112 | Normalized frequency of beta-sheet in alpha/beta class (Palau et al., 1981) |
| | PONP800101 | Surrounding hydrophobicity in folded form (Ponnuswamy et al., 1980) |
| | PONP800108 | Average number of surrounding residues (Ponnuswamy et al., 1980) |
| | QIAN880117 | Weights for beta-sheet at the window position of -3 (Qian-Sejnowski, 1988) |
| | QIAN880139 | Weights for coil at the window position of 6 (Qian-Sejnowski, 1988) |
| | RACS770103 | Side chain orientational preference (Rackovsky-Scheraga, 1977) |
| | RICJ880101 | Relative preference value at N" (Richardson-Richardson, 1988) |
| | RICJ880102 | Relative preference value at N' (Richardson-Richardson, 1988) |
| | ROBB760110 | Information measure for middle turn (Robson-Suzuki, 1976) |
| | TANS770106 | Normalized frequency of chain reversal D (Tanaka-Scheraga, 1977) |
| | TANS770107 | Normalized frequency of left-handed helix (Tanaka-Scheraga, 1977) |
| | VELV850101 | Electron-ion interaction potential (Veljkovic et al., 1985) |
| | VENT840101 | Bitterness (Venanzi, 1984) |
| | WEBA780101 | RF value in high salt chromatography (Weber-Lacey, 1978) |
| | ZIMJ680101 | Hydrophobicity (Zimmerman et al., 1968) |
| | AURR980101 | Normalized positional residue frequency at helix termini N4'(Aurora-Rose, 1998) |
| | AURR980103 | Normalized positional residue frequency at helix termini N" (Aurora-Rose, 1998) |
| | AURR980106 | Normalized positional residue frequency at helix termini N1 (Aurora-Rose, 1998) |
| | AURR980118 | Normalized positional residue frequency at helix termini C" (Aurora-Rose, 1998) |
| | AURR980120 | Normalized positional residue frequency at helix termini C4' (Aurora-Rose, 1998) |
| | VINM940102 | Normalized flexibility parameters (B-values) for each residue surrounded by none rigid neighbours (Vihinen et al., 1994) |
| | FUKS010101 | Surface composition of amino acids in intracellular proteins of thermophiles (percent) (Fukuchi-Nishikawa, 2001) |
| | FUKS010102 | Surface composition of amino acids in intracellular proteins of mesophiles |

| | | |
|---|---|---|
| | | (percent) (Fukuchi-Nishikawa, 2001) |
| | FUKS010103 | Surface composition of amino acids in extracellular proteins of mesophiles (percent) (Fukuchi-Nishikawa, 2001) |
| | FUKS010109 | Entire chain composition of amino acids in intracellular proteins of thermophiles (percent) (Fukuchi-Nishikawa, 2001) |
| | COSI940101 | Electron-ion interaction potential values (Cosic, 1994) |
| | BASU050103 | Interactivity scale obtained by maximizing the mean of correlation coefficient over pairs of sequences sharing the TIM barrel fold (Bastolla et al., 2005) |
| | GEOR030105 | Linker propensity from small dataset (linker length is less than six residues) (George-Heringa, 2003) |
| | KARS160106 | Radius (minimum eccentricity) (Karkbara-Knisley, 2016) |
| | KARS160108 | Average weighted degree (total degree, divided by the number of vertices) (Karkbara-Knisley, 2016) |
| | KARS160120 | Weighted minimum eigenvalue based on the atomic numbers (Karkbara-Knisley, 2016) |
| SF3B1 | ZASB820101 | Dependence of partition coefficient on ionic strength (Zaslavsky et al., 1982) |
| | ZIMJ680101 | Hydrophobicity (Zimmerman et al., 1968) |
| | ZIMJ680102 | Bulkiness (Zimmerman et al., 1968) |
| | ZIMJ680103 | Polarity (Zimmerman et al., 1968) |
| | ZIMJ680104 | Isoelectric point (Zimmerman et al., 1968) |
| | ZIMJ680105 | RF rank (Zimmerman et al., 1968) |
| | AURR980101 | Normalized positional residue frequency at helix termini N4'(Aurora-Rose, 1998) |
| | AURR980102 | Normalized positional residue frequency at helix termini N"' (Aurora-Rose, 1998) |
| | AURR980103 | Normalized positional residue frequency at helix termini N" (Aurora-Rose, 1998) |
| | AURR980104 | Normalized positional residue frequency at helix termini N'(Aurora-Rose, 1998) |
| | AURR980105 | Normalized positional residue frequency at helix termini Nc (Aurora-Rose, 1998) |
| | AURR980106 | Normalized positional residue frequency at helix termini N1 (Aurora-Rose, 1998) |
| | AURR980107 | Normalized positional residue frequency at helix termini N2 (Aurora-Rose, 1998) |
| | AURR980108 | Normalized positional residue frequency at helix termini N3 (Aurora-Rose, 1998) |
| | AURR980109 | Normalized positional residue frequency at helix termini N4 (Aurora-Rose, 1998) |
| | AURR980110 | Normalized positional residue frequency at helix termini N5 (Aurora-Rose, 1998) |
| | AURR980111 | Normalized positional residue frequency at helix termini C5 (Aurora-Rose, 1998) |
| | AURR980112 | Normalized positional residue frequency at helix termini C4 (Aurora-Rose, 1998) |
| | AURR980113 | Normalized positional residue frequency at helix termini C3 (Aurora-Rose, 1998) |
| | AURR980114 | Normalized positional residue frequency at helix termini C2 (Aurora-Rose, 1998) |
| | AURR980115 | Normalized positional residue frequency at helix termini C1 (Aurora-Rose, 1998) |
| | AURR980116 | Normalized positional residue frequency at helix termini Cc (Aurora-Rose, 1998) |
| | AURR980117 | Normalized positional residue frequency at helix termini C' (Aurora-Rose, 1998) |
| | AURR980118 | Normalized positional residue frequency at helix termini C" (Aurora-Rose, 1998) |
| | AURR980119 | Normalized positional residue frequency at helix termini C"' (Aurora- |

| | | |
|---|---|---|
| | | Rose, 1998) |
| | AURR980120 | Normalized positional residue frequency at helix termini C4' (Aurora-Rose, 1998) |
| | ONEK900101 | Delta G values for the peptides extrapolated to 0 M urea (O'Neil-DeGrado, 1990) |
| | ONEK900102 | Helix formation parameters (delta delta G) (O'Neil-DeGrado, 1990) |
| | VINM940101 | Normalized flexibility parameters (B-values), average (Vihinen et al., 1994) |
| | VINM940102 | Normalized flexibility parameters (B-values) for each residue surrounded by none rigid neighbours (Vihinen et al., 1994) |
| | VINM940103 | Normalized flexibility parameters (B-values) for each residue surrounded by one rigid neighbours (Vihinen et al., 1994) |
| | VINM940104 | Normalized flexibility parameters (B-values) for each residue surrounded by two rigid neighbours (Vihinen et al., 1994) |
| | MUNV940101 | Free energy in alpha-helical conformation (Munoz-Serrano, 1994) |
| | MUNV940102 | Free energy in alpha-helical region (Munoz-Serrano, 1994) |
| | MUNV940103 | Free energy in beta-strand conformation (Munoz-Serrano, 1994) |
| | MUNV940104 | Free energy in beta-strand region (Munoz-Serrano, 1994) |
| | MUNV940105 | Free energy in beta-strand region (Munoz-Serrano, 1994) |
| | WIMW960101 | Free energies of transfer of AcWl-X-LL peptides from bilayer interface to water (Wimley-White, 1996) |
| | KIMC930101 | Thermodynamic beta sheet propensity (Kim-Berg, 1993) |
| | MONM990101 | Turn propensity scale for transmembrane helices (Monne et al., 1999) |
| | BLAM930101 | Alpha helix propensity of position 44 in T4 lysozyme (Blaber et al., 1993) |
| | PARS000101 | p-Values of mesophilic proteins based on the distributions of B values (Parthasarathy-Murthy, 2000) |
| | PARS000102 | p-Values of thermophilic proteins based on the distributions of B values (Parthasarathy-Murthy, 2000) |
| | KUMS000101 | Distribution of amino acid residues in the 18 non-redundant families of thermophilic proteins (Kumar et al., 2000) |
| | KUMS000102 | Distribution of amino acid residues in the 18 non-redundant families of mesophilic proteins (Kumar et al., 2000) |
| | KUMS000103 | Distribution of amino acid residues in the alpha-helices in thermophilic proteins (Kumar et al., 2000) |
| | KUMS000104 | Distribution of amino acid residues in the alpha-helices in mesophilic proteins (Kumar et al., 2000) |
| | TAKK010101 | Side-chain contribution to protein stability (kJ/mol) (Takano-Yutani, 2001) |
| | FODM020101 | Propensity of amino acids within pi-helices (Fodje-Al-Karadaghi, 2002) |
| | NADH010101 | Hydropathy scale based on self-information values in the two-state model (5% accessibility) (Naderi-Manesh et al., 2001) |
| | NADH010102 | Hydropathy scale based on self-information values in the two-state model (9% accessibility) (Naderi-Manesh et al., 2001) |
| | NADH010103 | Hydropathy scale based on self-information values in the two-state model (16% accessibility) (Naderi-Manesh et al., 2001) |
| | NADH010104 | Hydropathy scale based on self-information values in the two-state model (20% accessibility) (Naderi-Manesh et al., 2001) |
| | NADH010105 | Hydropathy scale based on self-information values in the two-state model (25% accessibility) (Naderi-Manesh et al., 2001) |
| | NADH010106 | Hydropathy scale based on self-information values in the two-state model (36% accessibility) (Naderi-Manesh et al., 2001) |
| | NADH010107 | Hydropathy scale based on self-information values in the two-state model (50% accessibility) (Naderi-Manesh et al., 2001) |
| | MONM990201 | Averaged turn propensities in a transmembrane helix (Monne et al., 1999) |
| | KOEP990101 | Alpha-helix propensity derived from designed sequences (Koehl-Levitt, 1999) |
| | KOEP990102 | Beta-sheet propensity derived from designed sequences (Koehl-Levitt, 1999) |
| | CEDJ970101 | Composition of amino acids in extracellular proteins (percent) (Cedano et al., 1997) |

| | | |
|---|---|---|
| | CEDJ970102 | Composition of amino acids in anchored proteins (percent) (Cedano et al., 1997) |
| | CEDJ970103 | Composition of amino acids in membrane proteins (percent) (Cedano et al., 1997) |
| | CEDJ970104 | Composition of amino acids in intracellular proteins (percent) (Cedano et al., 1997) |
| | CEDJ970105 | Composition of amino acids in nuclear proteins (percent) (Cedano et al., 1997) |
| | FUKS010101 | Surface composition of amino acids in intracellular proteins of thermophiles (percent) (Fukuchi-Nishikawa, 2001) |
| | FUKS010102 | Surface composition of amino acids in intracellular proteins of mesophiles (percent) (Fukuchi-Nishikawa, 2001) |
| | FUKS010103 | Surface composition of amino acids in extracellular proteins of mesophiles (percent) (Fukuchi-Nishikawa, 2001) |
| | FUKS010104 | Surface composition of amino acids in nuclear proteins (percent) (Fukuchi-Nishikawa, 2001) |
| | FUKS010105 | Interior composition of amino acids in intracellular proteins of thermophiles (percent) (Fukuchi-Nishikawa, 2001) |
| | FUKS010106 | Interior composition of amino acids in intracellular proteins of mesophiles (percent) (Fukuchi-Nishikawa, 2001) |
| | FUKS010107 | Interior composition of amino acids in extracellular proteins of mesophiles (percent) (Fukuchi-Nishikawa, 2001) |
| | FUKS010108 | Interior composition of amino acids in nuclear proteins (percent) (Fukuchi-Nishikawa, 2001) |
| | FUKS010109 | Entire chain composition of amino acids in intracellular proteins of thermophiles (percent) (Fukuchi-Nishikawa, 2001) |
| | FUKS010110 | Entire chain composition of amino acids in intracellular proteins of mesophiles (percent) (Fukuchi-Nishikawa, 2001) |
| | FUKS010111 | Entire chain composition of amino acids in extracellular proteins of mesophiles (percent) (Fukuchi-Nishikawa, 2001) |
| | FUKS010112 | Entire chain compositino of amino acids in nuclear proteins (percent) (Fukuchi-Nishikawa, 2001) |
| | MITS020101 | Amphiphilicity index (Mitaku et al., 2002) |
| | TSAJ990101 | Volumes including the crystallographic waters using the ProtOr (Tsai et al., 1999) |
| | TSAJ990102 | Volumes not including the crystallographic waters using the ProtOr (Tsai et al., 1999) |
| | COSI940101 | Electron-ion interaction potential values (Cosic, 1994) |
| | PONP930101 | Hydrophobicity scales (Ponnuswamy, 1993) |
| | WILM950101 | Hydrophobicity coefficient in RP-HPLC, C18 with 0.1%TFA/MeCN/H2O (Wilce et al. 1995) |
| | WILM950102 | Hydrophobicity coefficient in RP-HPLC, C8 with 0.1%TFA/MeCN/H2O (Wilce et al. 1995) |
| | WILM950103 | Hydrophobicity coefficient in RP-HPLC, C4 with 0.1%TFA/MeCN/H2O (Wilce et al. 1995) |
| | WILM950104 | Hydrophobicity coefficient in RP-HPLC, C18 with 0.1%TFA/2-PrOH/MeCN/H2O (Wilce et al. 1995) |
| | KUHL950101 | Hydrophilicity scale (Kuhn et al., 1995) |
| | GUOD860101 | Retention coefficient at pH 2 (Guo et al., 1986) |
| | JURD980101 | Modified Kyte-Doolittle hydrophobicity scale (Juretic et al., 1998) |
| | BASU050101 | Interactivity scale obtained from the contact matrix (Bastolla et al., 2005) |
| | BASU050102 | Interactivity scale obtained by maximizing the mean of correlation coefficient over single-domain globular proteins (Bastolla et al., 2005) |
| | BASU050103 | Interactivity scale obtained by maximizing the mean of correlation coefficient over pairs of sequences sharing the TIM barrel fold (Bastolla et al., 2005) |
| | SUYM030101 | Linker propensity index (Suyama-Ohara, 2003) |
| | PUNT030101 | Knowledge-based membrane-propensity scale from 1D_Helix in MPtopo databases (Punta-Maritan, 2003) |
| | PUNT030102 | Knowledge-based membrane-propensity scale from 3D_Helix in MPtopo |

| | | |
|---|---|---|
| | | databases (Punta-Maritan, 2003) |
| | GEOR030101 | Linker propensity from all dataset (George-Heringa, 2003) |
| | GEOR030102 | Linker propensity from 1-linker dataset (George-Heringa, 2003) |
| | GEOR030103 | Linker propensity from 2-linker dataset (George-Heringa, 2003) |
| | GEOR030104 | Linker propensity from 3-linker dataset (George-Heringa, 2003) |
| | GEOR030105 | Linker propensity from small dataset (linker length is less than six residues) (George-Heringa, 2003) |
| | GEOR030106 | Linker propensity from medium dataset (linker length is between six and 14 residues) (George-Heringa, 2003) |
| | GEOR030107 | Linker propensity from long dataset (linker length is greater than 14 residues) (George-Heringa, 2003) |
| | GEOR030108 | Linker propensity from helical (annotated by DSSP) dataset (George-Heringa, 2003) |
| | GEOR030109 | Linker propensity from non-helical (annotated by DSSP) dataset (George-Heringa, 2003) |
| | ZHOH040101 | The stability scale from the knowledge-based atom-atom potential (Zhou-Zhou, 2004) |
| | ZHOH040102 | The relative stability scale extracted from mutation experiments (Zhou-Zhou, 2004) |
| | ZHOH040103 | Buriability (Zhou-Zhou, 2004) |
| | BAEK050101 | Linker index (Bae et al., 2005) |
| | HARY940101 | Mean volumes of residues buried in protein interiors (Harpaz et al., 1994) |
| | PONJ960101 | Average volumes of residues (Pontius et al., 1996) |
| | DIGM050101 | Hydrostatic pressure asymmetry index, PAI (Di Giulio, 2005) |
| | WOLR790101 | Hydrophobicity index (Wolfenden et al., 1979) |
| | OLSK800101 | Average internal preferences (Olsen, 1980) |
| | KIDA850101 | Hydrophobicity-related index (Kidera et al., 1985) |
| | GUYH850102 | Apparent partition energies calculated from Wertz-Scheraga index (Guy, 1985) |
| | GUYH850104 | Apparent partition energies calculated from Janin index (Guy, 1985) |
| | GUYH850105 | Apparent partition energies calculated from Chothia index (Guy, 1985) |
| | JACR890101 | Weights from the IFH scale (Jacobs-White, 1989) |
| | COWR900101 | Hydrophobicity index, 3.0 pH (Cowan-Whittaker, 1990) |
| | BLAS910101 | Scaled side chain hydrophobicity values (Black-Mould, 1991) |
| | CASG920101 | Hydrophobicity scale from native protein structures (Casari-Sippl, 1992) |
| | CORJ870101 | NNEIG index (Cornette et al., 1987) |
| | CORJ870102 | SWEIG index (Cornette et al., 1987) |
| | CORJ870103 | PRIFT index (Cornette et al., 1987) |
| | CORJ870104 | PRILS index (Cornette et al., 1987) |
| | CORJ870105 | ALTFT index (Cornette et al., 1987) |
| | CORJ870106 | ALTLS index (Cornette et al., 1987) |
| | CORJ870107 | TOTFT index (Cornette et al., 1987) |
| | CORJ870108 | TOTLS index (Cornette et al., 1987) |
| | MIYS990101 | Relative partition energies derived by the Bethe approximation (Miyazawa-Jernigan, 1999) |
| | MIYS990102 | Optimized relative partition energies - method A (Miyazawa-Jernigan, 1999) |
| | MIYS990103 | Optimized relative partition energies - method B (Miyazawa-Jernigan, 1999) |
| | MIYS990104 | Optimized relative partition energies - method C (Miyazawa-Jernigan, 1999) |
| | MIYS990105 | Optimized relative partition energies - method D (Miyazawa-Jernigan, 1999) |
| | ENGD860101 | Hydrophobicity index (Engelman et al., 1986) |
| | FASG890101 | Hydrophobicity index (Fasman, 1989) |
| | KARS160101 | Number of vertices (order of the graph) (Karkbara-Knisley, 2016) |
| | KARS160102 | Number of edges (size of the graph) (Karkbara-Knisley, 2016) |
| | KARS160103 | Total weighted degree of the graph (obtained by adding all the weights of all the vertices) (Karkbara-Knisley, 2016) |
| | KARS160104 | Weighted domination number (Karkbara-Knisley, 2016) |

| | | |
|---|---|---|
| | KARS160105 | Average eccentricity (Karkbara-Knisley, 2016) |
| | KARS160106 | Radius (minimum eccentricity) (Karkbara-Knisley, 2016) |
| | KARS160107 | Diameter (maximum eccentricity) (Karkbara-Knisley, 2016) |
| | KARS160108 | Average weighted degree (total degree, divided by the number of vertices) (Karkbara-Knisley, 2016) |
| | KARS160109 | Maximum eigenvalue of the weighted Laplacian matrix of the graph (Karkbara-Knisley, 2016) |
| | KARS160110 | Minimum eigenvalue of the weighted Laplacian matrix of the graph (Karkbara-Knisley, 2016) |
| | KARS160111 | Average eigenvalue of the Laplacian matrix of the the graph (Karkbara-Knisley, 2016) |
| | KARS160112 | Second smallest eigenvalue of the Laplacian matrix of the graph (Karkbara-Knisley, 2016) |
| | KARS160113 | Weighted domination number using the atomic number (Karkbara-Knisley, 2016) |
| | KARS160114 | Average weighted eccentricity based on the the atomic number (Karkbara-Knisley, 2016) |
| | KARS160115 | Weighted radius based on the atomic number (minimum eccentricity) (Karkbara-Knisley, 2016) |
| | KARS160116 | Weighted diameter based on the atomic number (maximum eccentricity) (Karkbara-Knisley, 2016) |
| | KARS160117 | Total weighted atomic number of the graph (obtained by summing all the atomic number of each of the vertices in the graph) (Karkbara-Knisley, 2016) |
| | KARS160118 | Average weighted atomic number or degree based on atomic number in the graph (Karkbara-Knisley, 2016) |
| | KARS160119 | Weighted maximum eigenvalue based on the atomic numbers (Karkbara-Knisley, 2016) |
| | KARS160120 | Weighted minimum eigenvalue based on the atomic numbers (Karkbara-Knisley, 2016) |
| | KARS160121 | Weighted average eigenvalue based on the atomic numbers (Karkbara-Knisley, 2016) |
| | KARS160122 | Weighted second smallest eigenvalue of the weighted Laplacian matrix (Karkbara-Knisley, 2016) |
| SPEN | CHAM830101 | The Chou-Fasman parameter of the coil conformation (Charton-Charton, 1983) |
| | CHAM830102 | A parameter defined from the residuals obtained from the best correlation of the Chou-Fasman parameter of beta-sheet (Charton-Charton, 1983) |
| | CHOP780101 | Normalized frequency of beta-turn (Chou-Fasman, 1978a) |
| | CHOP780203 | Normalized frequency of beta-turn (Chou-Fasman, 1978b) |
| | CHOP780210 | Normalized frequency of N-terminal non beta region (Chou-Fasman, 1978b) |
| | CHOP780211 | Normalized frequency of C-terminal non beta region (Chou-Fasman, 1978b) |
| | CHOP780216 | Normalized frequency of the 2nd and 3rd residues in turn (Chou-Fasman, 1978b) |
| | GARJ730101 | Partition coefficient (Garel et al., 1973) |
| | GEIM800108 | Aperiodic indices (Geisow-Roberts, 1980) |
| | LEVM760107 | van der Waals parameter epsilon (Levitt, 1976) |
| | LEVM780103 | Normalized frequency of reverse turn, with weights (Levitt, 1978) |
| | MAXF760105 | Normalized frequency of zeta L (Maxfield-Scheraga, 1976) |
| | MCMT640101 | Refractivity (McMeekin et al., 1964), Cited by Jones (1975) |
| | NAGK730103 | Normalized frequency of coil (Nagano, 1973) |
| | PALJ810110 | Normalized frequency of beta-sheet in all-beta class (Palau et al., 1981) |
| | PONP800106 | Surrounding hydrophobicity in turn (Ponnuswamy et al., 1980) |
| | PRAM900104 | Relative frequency in reverse-turn (Prabhakaran, 1990) |
| | QIAN880115 | Weights for beta-sheet at the window position of -5 (Qian-Sejnowski, 1988) |
| | QIAN880116 | Weights for beta-sheet at the window position of -4 (Qian-Sejnowski, 1988) |

| | | |
|---|---|---|
| | ROBB760105 | Information measure for extended (Robson-Suzuki, 1976) |
| | ROBB760111 | Information measure for C-terminal turn (Robson-Suzuki, 1976) |
| | SNEP660104 | Principal component IV (Sneath, 1966) |
| | TANS770106 | Normalized frequency of chain reversal D (Tanaka-Scheraga, 1977) |
| | VASM830101 | Relative population of conformational state A (Vasquez et al., 1983) |
| | VELV850101 | Electron-ion interaction potential (Veljkovic et al., 1985) |
| | AURR980101 | Normalized positional residue frequency at helix termini N4'(Aurora-Rose, 1998) |
| | MITS020101 | Amphiphilicity index (Mitaku et al., 2002) |
| | COSI940101 | Electron-ion interaction potential values (Cosic, 1994) |
| | WILM950103 | Hydrophobicity coefficient in RP-HPLC, C4 with 0.1%TFA/MeCN/H2O (Wilce et al. 1995) |
| | GEOR030105 | Linker propensity from small dataset (linker length is less than six residues) (George-Heringa, 2003) |
| | CORJ870104 | PRILS index (Cornette et al., 1987) |
| | KARS160118 | Average weighted atomic number or degree based on atomic number in the graph (Karkbara-Knisley, 2016) |
| TET2 | BUNA790101 | alpha-NH chemical shifts (Bundi-Wuthrich, 1979) |
| | FAUJ880105 | STERIMOL minimum width of the side chain (Fauchere et al., 1988) |
| | GEIM800105 | Beta-strand indices (Geisow-Roberts, 1980) |
| | HOPA770101 | Hydration number (Hopfinger, 1971), Cited by Charton-Charton (1982) |
| | ISOY800102 | Normalized relative frequency of extended structure (Isogai et al., 1980) |
| | LEVM780102 | Normalized frequency of beta-sheet, with weights (Levitt, 1978) |
| | LEVM780105 | Normalized frequency of beta-sheet, unweighted (Levitt, 1978) |
| | LIFS790103 | Conformational preference for antiparallel beta-strands (Lifson-Sander, 1979) |
| | MANP780101 | Average surrounding hydrophobicity (Manavalan-Ponnuswamy, 1978) |
| | MAXF760102 | Normalized frequency of extended structure (Maxfield-Scheraga, 1976) |
| | MEEJ810102 | Retention coefficient in NaH2PO4 (Meek-Rossetti, 1981) |
| | NAKH900113 | Ratio of average and computed composition (Nakashima et al., 1990) |
| | PALJ810105 | Normalized frequency of turn from LG (Palau et al., 1981) |
| | PALJ810108 | Normalized frequency of alpha-helix in alpha+beta class (Palau et al., 1981) |
| | PALJ810110 | Normalized frequency of beta-sheet in all-beta class (Palau et al., 1981) |
| | PALJ810113 | Normalized frequency of turn in all-alpha class (Palau et al., 1981) |
| | PONP800103 | Average gain ratio in surrounding hydrophobicity (Ponnuswamy et al., 1980) |
| | PRAM900103 | Relative frequency in beta-sheet (Prabhakaran, 1990) |
| | PTIO830102 | Beta-coil equilibrium constant (Ptitsyn-Finkelstein, 1983) |
| | QIAN880102 | Weights for alpha-helix at the window position of -5 (Qian-Sejnowski, 1988) |
| | QIAN880119 | Weights for beta-sheet at the window position of -1 (Qian-Sejnowski, 1988) |
| | ROBB760109 | Information measure for N-terminal turn (Robson-Suzuki, 1976) |
| | TANS770103 | Normalized frequency of extended structure (Tanaka-Scheraga, 1977) |
| | WERD780102 | Free energy change of epsilon(i) to epsilon(ex) (Wertz-Scheraga, 1978) |
| | YUTK870103 | Activation Gibbs energy of unfolding, pH7.0 (Yutani et al., 1987) |
| | YUTK870104 | Activation Gibbs energy of unfolding, pH9.0 (Yutani et al., 1987) |
| | AURR980101 | Normalized positional residue frequency at helix termini N4'(Aurora-Rose, 1998) |
| | AURR980106 | Normalized positional residue frequency at helix termini N1 (Aurora-Rose, 1998) |
| | AURR980109 | Normalized positional residue frequency at helix termini N4 (Aurora-Rose, 1998) |
| | AURR980120 | Normalized positional residue frequency at helix termini C4' (Aurora-Rose, 1998) |
| | NADH010104 | Hydropathy scale based on self-information values in the two-state model (20% accessibility) (Naderi-Manesh et al., 2001) |
| | KOEP990101 | Alpha-helix propensity derived from designed sequences (Koehl-Levitt, 1999) |

| | | |
|---|---|---|
| | FUKS010111 | Entire chain composition of amino acids in extracellular proteins of mesophiles (percent) (Fukuchi-Nishikawa, 2001) |
| | BASU050103 | Interactivity scale obtained by maximizing the mean of correlation coefficient over pairs of sequences sharing the TIM barrel fold (Bastolla et al., 2005) |
| | KARS160120 | Weighted minimum eigenvalue based on the atomic numbers (Karkbara-Knisley, 2016) |
| TP53 | BUNA790101 | alpha-NH chemical shifts (Bundi-Wuthrich, 1979) |
| | CHAM830108 | A parameter of charge transfer donor capability (Charton-Charton, 1983) |
| | DAWD720101 | Size (Dawson, 1972) |
| | FASG760104 | pK-N (Fasman, 1976) |
| | FASG760105 | pK-C (Fasman, 1976) |
| | FAUJ880104 | STERIMOL length of the side chain (Fauchere et al., 1988) |
| | FINA910102 | Helix initiation parameter at posision i,i+1,i+2 (Finkelstein et al., 1991) |
| | FINA910103 | Helix termination parameter at posision j-2,j-1,j (Finkelstein et al., 1991) |
| | FINA910104 | Helix termination parameter at posision j+1 (Finkelstein et al., 1991) |
| | GARJ730101 | Partition coefficient (Garel et al., 1973) |
| | GEIM800107 | Beta-strand indices for alpha/beta-proteins (Geisow-Roberts, 1980) |
| | ISOY800107 | Normalized relative frequency of double bend (Isogai et al., 1980) |
| | JANJ780101 | Average accessible surface area (Janin et al., 1978) |
| | KHAG800101 | The Kerr-constant increments (Khanarian-Moore, 1980) |
| | LEVM760107 | van der Waals parameter epsilon (Levitt, 1976) |
| | NAGK730102 | Normalized frequency of bata-structure (Nagano, 1973) |
| | NAKH900104 | Normalized composition of mt-proteins (Nakashima et al., 1990) |
| | NAKH900106 | Normalized composition from animal (Nakashima et al., 1990) |
| | NAKH900108 | Normalized composition from fungi and plant (Nakashima et al., 1990) |
| | NAKH900112 | Transmembrane regions of mt-proteins (Nakashima et al., 1990) |
| | OOBM770103 | Long range non-bonded energy per atom (Oobatake-Ooi, 1977) |
| | PALJ810109 | Normalized frequency of alpha-helix in alpha/beta class (Palau et al., 1981) |
| | PTIO830101 | Helix-coil equilibrium constant (Ptitsyn-Finkelstein, 1983) |
| | QIAN880108 | Weights for alpha-helix at the window position of 1 (Qian-Sejnowski, 1988) |
| | QIAN880136 | Weights for coil at the window position of 3 (Qian-Sejnowski, 1988) |
| | RACS770103 | Side chain orientational preference (Rackovsky-Scheraga, 1977) |
| | RACS820103 | Average relative fractional occurrence in AL(i) (Rackovsky-Scheraga, 1982) |
| | RACS820114 | Value of theta(i-1) (Rackovsky-Scheraga, 1982) |
| | ROBB760104 | Information measure for C-terminal helix (Robson-Suzuki, 1976) |
| | TANS770105 | Normalized frequency of chain reversal S (Tanaka-Scheraga, 1977) |
| | TANS770108 | Normalized frequency of zeta R (Tanaka-Scheraga, 1977) |
| | TANS770109 | Normalized frequency of coil (Tanaka-Scheraga, 1977) |
| | WERD780103 | Free energy change of alpha(Ri) to alpha(Rh) (Wertz-Scheraga, 1978) |
| | ZASB820101 | Dependence of partition coefficient on ionic strength (Zaslavsky et al., 1982) |
| | AURR980106 | Normalized positional residue frequency at helix termini N1 (Aurora-Rose, 1998) |
| | AURR980107 | Normalized positional residue frequency at helix termini N2 (Aurora-Rose, 1998) |
| | ONEK900101 | Delta G values for the peptides extrapolated to 0 M urea (O'Neil-DeGrado, 1990) |
| | ONEK900102 | Helix formation parameters (delta delta G) (O'Neil-DeGrado, 1990) |
| | BLAM930101 | Alpha helix propensity of position 44 in T4 lysozyme (Blaber et al., 1993) |
| | TSAJ990101 | Volumes including the crystallographic waters using the ProtOr (Tsai et al., 1999) |
| | TSAJ990102 | Volumes not including the crystallographic waters using the ProtOr (Tsai et al., 1999) |
| | PUNT030101 | Knowledge-based membrane-propensity scale from 1D_Helix in MPtopo databases (Punta-Maritan, 2003) |
| | GEOR030105 | Linker propensity from small dataset (linker length is less than six |

| | | |
|---|---|---|
| | | residues) (George-Heringa, 2003) |
| | GEOR030109 | Linker propensity from non-helical (annotated by DSSP) dataset (George-Heringa, 2003) |
| | HARY940101 | Mean volumes of residues buried in protein interiors (Harpaz et al., 1994) |
| | GUYH850105 | Apparent partition energies calculated from Chothia index (Guy, 1985) |
| | CORJ870104 | PRILS index (Cornette et al., 1987) |
| | KARS160106 | Radius (minimum eccentricity) (Karkbara-Knisley, 2016) |
| | KARS160112 | Second smallest eigenvalue of the Laplacian matrix of the graph (Karkbara-Knisley, 2016) |
